# Integrating behavioural experimental findings into dynamical models to inform social change interventions

**DOI:** 10.1038/s41562-026-02417-4

**Published:** 2026-03-16

**Authors:** Radu Tănase, René Algesheimer, Manuel S. Mariani

**Affiliations:** Department of Business Administration, University of Zurich, Zurich, Switzerland

**Keywords:** Complex networks, Complex networks, Interdisciplinary studies

## Abstract

Addressing global challenges often involves stimulating the large-scale adoption of new products or behaviours. Research traditions that focus on individual decision-making suggest that achieving this objective requires identifying the drivers of individual discrete adoption choices. However, computational approaches rooted in complexity science focus on maximizing the propagation of a given product or behaviour throughout social networks of interconnected adopters. Here, by integrating discrete-choice modelling into the complex contagion theory, we propose a method to estimate individual-level thresholds to adoption. We validate the predictive power of this approach in two choice experiments. By integrating the estimated thresholds into computational simulations, we show that state-of-the-art seeding policies for initiating large-scale behavioural change might be suboptimal if they neglect individual-level behavioural drivers, which can be corrected through the proposed experimental method.

## Abstract (web summary)

Tănase et al. show how integrating discrete-choice modelling into the complex contagion theory reveals individual differences in adoption of innovations and informs social network interventions.

## Main

Preventing, mitigating and solving some of the most pressing challenges faced by mankind often requires the design of effective technological and behavioural solutions, but even the most effective solutions would fail to make an impact without their large-scale adoption by the world population. For some global challenges, such as the coronavirus disease 2019 pandemic, policymakers around the world have often enforced the required behaviours to counteract the pandemic’s most severe effects in a top-down fashion, for example, by restricting travel, requiring mandatory mask wearing in public spaces and isolating positive cases. For other global challenges, even when technological and behavioural solutions to mitigate the challenge are available, policymakers are unwilling to or cannot fully oblige the citizens to adopt them^1^. This is the case, for example, for shifting individuals’ transportation, nutrition and building design choices towards sustainable alternatives, which might substantially reduce greenhouse gas emissions^2^. Many sustainable technologies and behaviours are well known, but triggering their large-scale adoption remains elusive^1^.

To generate scientific insights that could inform societal change policies, a tension between two approaches exists. A well-established research tradition—for example, in microeconomics, consumer behaviour and psychology—focuses on modelling the individual-level decision-making process, seeking to capture the drivers of individual-level choices and potentially nudge new behaviours^3–6^. These approaches typically capture the complexity of individual decision-making processes, which leads (in the best case) to a precise image of individual processes. However, because of the feedback mechanisms and consequent non-linearities that characterize collective behaviours, their conclusions may not be directly extrapolated to the collective level^7–13^.

Conversely, computational approaches to understanding collective behaviour in complex systems typically formulate parsimonious models on individual-level behaviours under simplifying assumptions. These approaches—often rooted in statistical physics and agent-based modelling—have found applications ranging from materials composed of simple particles to systems composed of more sophisticated units such as flocks^14^, swarms^15^, crowds^10^ and societies^11^. In the context of social change, computational approaches leverage agent-based simulations to determine the system-level implications of interconnected individual-level choices^7–13,16,17^. These models enable a comprehensive understanding of the possible macro-level behaviours of a system, but when not calibrated with empirical individual processes, they may fail to identify the best intervention for a given scenario^18^. Individual-level and computational approaches are mainly developed in parallel. Scholars from various disciplines have argued that the disconnect between our understanding of individual decision-making and social network dynamics limits policymakers’ ability to stimulate effective collective behavioural responses to increasingly complex global challenges^19^, and therefore have called for data-driven efforts to integrate the two perspectives^13,16,19–22^.

Here, we demonstrate how the two approaches can be integrated in the context of new product or behaviour adoption, and how such an integration can inform network interventions for social change. The leading framework to model social change is provided by the complex contagion theory^7,8,23–27^, which posits that, when an adoption choice involves a substantial level of personal investment (for example, effort, monetary cost and personal or reputational risk), it requires a sufficient level of social reinforcement from the decision-maker’s social contacts who already adopted the new product or behaviour^1,23–25^. This idea is incorporated in the threshold model of new product diffusion^8,23^, where individual *n* adopts a given product (or behaviour) *i* if and only if at least a threshold fraction *τ*_*ni*_ of their social contacts have already adopted the product. According to the theory, threshold-based diffusion models describe accurately how new ideas and behaviours spread in many policy-relevant contexts, including the adoption of healthy behaviours^28–31^, online social platforms^32–34^, new technologies^35,36^, views on political and controversial topics^37–40^, social movements and political protests^7,41,42^ and the abandonment of harmful cultural traditions^17^ (see ref. ^24^ for a comprehensive review). The relevance of the threshold distribution for diffusion outcomes^7,8,43,44^ seems to directly call for measuring individual-level thresholds from empirical data to calibrate threshold-based simulations. Yet surprisingly, despite early empirical works on historical diffusion data^43,45^, there is currently limited evidence on the empirical individual-level threshold levels, and a lack of empirically validated methods to estimate individual thresholds and integrate them into social network interventions^20,24,46^.

Here, we develop a model-based approach to estimate individual-level thresholds without requiring historical adoption or social network data, we validate it across two distinct empirical domains, and we demonstrate its usefulness to inform network interventions for social change. Pioneering empirical efforts^43,45,47^ calculated individual thresholds from historical adoption data by relying on simple heuristics such as the percentage of adopters in one’s personal network at adoption time. While intuitive and practical, these heuristics are difficult to refute in practice, as observed in ref. ^48^. Considerable challenges occur when one seeks to measure a given individual’s threshold for a given product from historical adoption data: if a given individual never adopted the product, we cannot determine at which higher level of social signal, if any, they would have adopted it; if they did adopt, we cannot infer for which lower level of social signal they would have still adopted, as there is typically a time lag between when an individual’s threshold is reached and their actual adoption^43,47^. Besides, if the product is new, no historical adoption data exist.

**Fig. 1:**
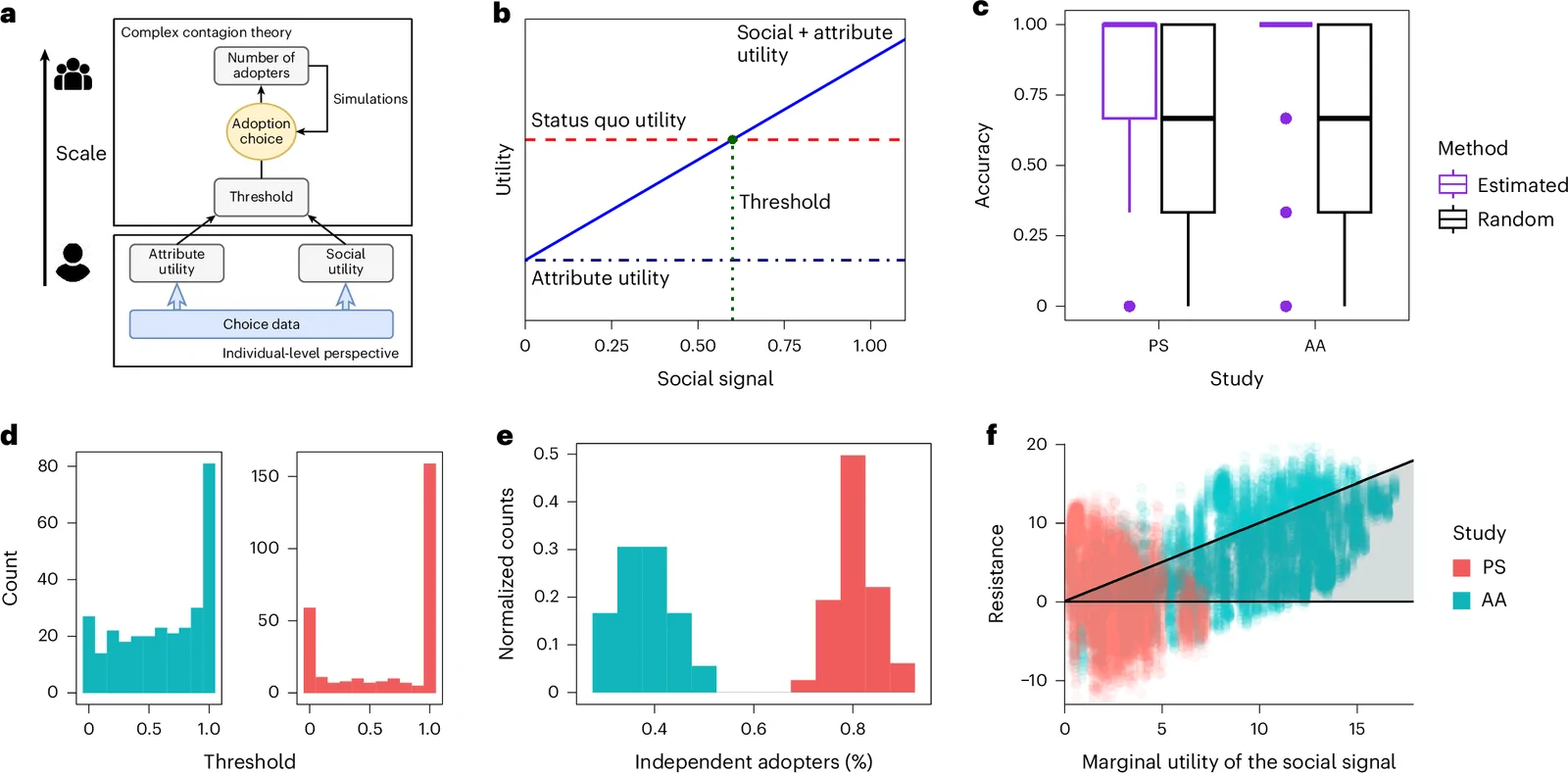
Estimating individual-level thresholds. **a**, Threshold-based diffusion models assume that individual-level adoption choices are determined by the decision-makers threshold. Conversely, individual-level perspectives focus on estimating individuals utilities of adopting from choice data. Utility-based approaches to behavioural change reconcile these two perspectives by reinterpreting the individual-level thresholds in terms of individuals attribute and social utilities^54,55^. **b**, For a susceptible adopter, the status quo utility (dashed horizontal red line) is initially larger than the utility from adopting, which is equal to the attribute utility (dashed horizontal blue line) when the social signal is zero. As the social signal increases, so does their utility from adopting (straight blue line), equal to the sum of the social and attribute utilities. The threshold (dashed vertical green line) is defined as the minimal level of social signal at which the utility from adopting exceeds the status-quo utility from not adopting. **c**, For both experiments, the individual-level thresholds estimated from experimental data hold out-of-sample predictive power, as illustrated by their superior accuracy compared with a random threshold baseline. In these box plots, the central line indicates the median, the box limits represent the first and third quartiles (interquartile range) and the whiskers represent data points that are within 1.5 × the interquartile range. Any points beyond that limit are shown as individual outliers. The number of observations used to calculate the accuracy of each method was 4,363 and 4,183 for the PS and AA study, respectively. **d**, In general, different products exhibit different threshold distributions, as illustrated by the two examples provided here (AA in blue and PS in orange). **e**, The distribution of the proportion of independent adopters (as opposed to susceptible adopters) is significantly lower for the AA experiment (blue) than for the PS experiment (orange), which highlights the importance of context for the distribution of individual thresholds. **f**, An individual is susceptible for adopting a given product when their resistance is positive and lower than the marginal utility of social signal. This condition corresponds to the grey area in the *γ*–*R* diagram where each individual-product pair is represented by a dot in the plane spanned by the marginal utility of social signal *γ* and resistance *R*. There are significantly more observations that fall within the grey area in the AA experiment than in the PS experiment, which explains the higher percentage of susceptible adopters in the AA experiment. Data in this panel is based on a sample of products, as described in Supplementary Note S2B. Source data.

To address these challenges, we draw on utility-based theories of behavioural change^49^, historically rooted in economics^50^ and consumer behaviour^51,52^. These theories assume that by adopting a certain new behaviour, individuals can gain or lose utility depending on whether the behaviour conforms with others’ behaviour or expectations^49^. Importantly, they provide analytical expressions linking individual thresholds to underlying utility parameters in various scenarios^53–55^. Motivated by these works, we adopt here a simple utility-based formulation that enables convenient estimation from choice data^54^. This perspective connects the complex contagion literature with the individual-level perspective (Fig. 1a).

We leverage a simple formula that links individuals’ thresholds to the drivers of individual-level adoption choices, which enables the measurement of individual-level thresholds from discrete-choice experiments without requiring detailed social network data. Results from two choice experiments demonstrate that individual-level thresholds can be estimated from choice data and used to make accurate out-of-sample choice predictions at the individual level. We show how the estimated thresholds can be integrated into policies to promote large-scale behavioural change. To this end, we compare the performance of policies aware of the estimated thresholds against traditional ones on the basis of social network centrality, and we determine under which conditions the threshold-aware policies outperform existing ones. For example, our findings reveal that the recently introduced complex centrality^46^ consistently outperforms state-of-the-art policies only when its calculation takes into account the estimated thresholds.

## Results

### From individual-level preferences to thresholds

We assume that, when evaluating a certain product (or behaviour) *i*, an individual is influenced by both the product’s attributes and the received social signal about the product. We assume that individual *n*’s utility from adopting product (or behaviour) *i* consists of two terms: the utility of the product attributes for $n\,({U}_{ni}^{({\rm{A}})})$ and the ‘social utility’^50^ derived from the social signal received by *n* about *i* (${U}_{ni}^{({\rm{S}})}$): ${U}_{ni}={U}_{ni}^{({\rm{A}})}+{U}_{ni}^{({\rm{S}})}$. Under this formulation, we define the adoption threshold as the minimal level of the social signal for which the utility of *n* from adopting *i* exceeds *n*’s status quo utility, that is, the utility from not adopting (${U}_{n}^{(0)}$) (Fig. 1b). In the following, it is convenient to define *n*’s resistance to adopting *i* as the gap between their status quo utility and the attribute utility from adopting *i*, that is, ${R}_{ni}={U}_{n}^{(0)}-{U}_{ni}^{({\rm{A}})}$. Assuming that *R*_*ni*_ > 0 and that the social utility is linear in the number of adopters (${U}_{ni}^{({\rm{S}})}={\gamma }_{n}{s}_{ni}$, where *s*_*ni*_ denotes the percentage of adopters of *i* within *n*’s social neighbourhood, and *γ*_*n*_ > 0 represents *n*’s marginal utility of the social signal), the adoption threshold can be expressed as^54^

1 $${\tau }_{ni}=\frac{{U}_{n}^{\,(0)}-{U}_{ni}^{\,({\rm{A}})}}{{\gamma }_{n}}=\frac{{R}_{ni}}{{\gamma }_{n}}.$$

This result is intuitive: the higher the individual’s resistance, or the lower the weight of the social signal on their choices, the higher their threshold. We assume—as is typical in discrete-choice modelling^3^—that the attribute utility is linear and separable in the attributes ${U}_{ni}^{(A)}={\sum }_{k=1}^{K}{\beta }_{nk}\,{x}_{ki}$, where *x*_*ki*_ encodes the extent to which alternative *i* exhibits attribute *k*, and *β*_*nk*_ is referred to as the partworth utility of *n* for attribute *k*.

An implication of equation (1) is that, if one is able to estimate individuals’ *R* and *γ* from choice data, one can directly calculate their threshold as well. Before providing the results of this procedure, we anticipate that, given estimates ${\widehat{R}}_{ni}$ and ${\widehat{\gamma }}_{n}$ of individual resistance and marginal utility from social signal, equation (1) can lead to multiple scenarios. We refer to individuals with $0 < {\widehat{R}}_{ni} < {\widehat{\gamma }}_{n}$ as susceptible adopters: for these individuals, equation (1) delivers ${\widehat{\tau }}_{ni}\in (0,1)$, which implies that their adoption choices for product *i* are influenced by different levels of social signal *s*_*ni*_ ∈ (0, 1). For individuals with ${\widehat{R}}_{ni} < 0$, equation (1) delivers ${\widehat{\tau }}_{ni} < 0$. Using the terminology of the innovation diffusion theory^56–59^, these individuals act as innovators with respect to their personal network^43^, as they adopt as soon as they become aware of product *i* regardless of the social signal’s strength; for practical purposes, their estimated threshold can be set to zero. Note that they may not necessarily be innovators with respect to the whole social system, as they may still adopt late if they are not exposed early on to the product^43^. For individuals with ${\widehat{R}}_{ni} > {\widehat{\gamma }}_{n}$, equation (1) delivers ${\widehat{\tau }}_{ni} > 1$; these individuals will never adopt product *i* regardless of the social signal’s strength; for practical purposes, their estimated threshold can be set to one. We refer to individuals with ${\widehat{R}}_{ni} < 0$ or ${\widehat{R}}_{ni} > {\widehat{\gamma }}_{n}$ as independent adopters, as their adoption choices are independent of the level of social signal.

This interpretation of the threshold has two important implications. First, we can unlock the connection between individual-level utilities and threshold-based diffusion processes, thereby connecting macro-level patterns of adoption with their micro-level drivers (Fig. 1a,b). Second, given empirical choice data, we can estimate the individual-level thresholds from choice data through well-established utility estimation algorithms^60^, which we demonstrate below.

### Experimental validation

To demonstrate the detectability of individual-level thresholds from choice data, we rely on choice-based conjoint experiments^61^ (see Supplementary Note S1A for an overview). In this class of experiments, widespread in consumer behaviour research, participants are exposed to a set of choices among hypothetical products (represented by combinations of attributes) and a status quo option (selecting none of the presented choices). In the designed experiments, detailed below, participants are exposed to product alternatives characterized additionally by varying levels of social signal. By analysing the resulting choice data through a well-established hierarchical Bayes (HB) algorithm^60^, we recover the relative weight of each attribute in determining each participant’s choices (that is, the parameters *β* above), the marginal utility of the social signal (*γ*), and the status quo utility (*U*^(0)^), which enables the calculation of individual thresholds through equation (1) (see Methods for details).

We ran two choice-based conjoint experiments, which cover two choice contexts where the complex contagion theory is expected to apply^24^: political views (energy policy support experiment, hereafter ‘PS’ experiment) and the adoption of a new technology under network externalities (instant messaging app adoption, hereafter ‘AA’ experiment). In the PS experiment, participants are presented with a set of energy policies regarding carbon-capturing technologies, and in each choice task, they are asked to select which policy they would support (if any). The policies differ in terms of five attributes (policy instrument, costs, beginning of policy implementation, required distance to residential areas and policy endorsers)^62^ and in their level of social signal (presented as the percentage of peers supporting the policy). The policies’ attributes—except for the social signal—are identical to those in ref. ^62^. In the AA experiment, participants are presented with a set of messaging apps and are asked to select which app they would consider installing (if any). The apps differ in terms of four attributes (accessibility, authentication method, customization level and support for video calls) and their level of social signal (percentage of friends already using the app). For both experiments, we collect choice data from participants recruited through Prolific, an online platform commonly used for sourcing high-quality respondents for research studies^63^. We recruited *N*_*R*_ = 296 participants in the PS experiment and *N*_*R*_ = 300 in the AA experiment, and we estimated the individual-level thresholds through the HB algorithm and equation (1). Refer to Methods and Supplementary Note S1 for details about the two studies and the parameter estimation.

Before attempting to understand the properties of the estimated thresholds, it is essential to validate the estimation method. If the estimated thresholds are accurate, we expect that they hold out-of-sample predictive power for individual choices in hold-out tasks that have not been used for the estimation. In particular, for an accurate set of estimated thresholds, we expect that the alternatives selected by the participants in hold-out tasks are predicted by the gap between the alternative’s social signal and the individual’s threshold for that alternative (Methods). By translating this remark into an accuracy function (see equation (4) in Methods), we can compare the accuracy of choice predictions on the basis of the estimated thresholds against those based on randomly extracted thresholds from a uniform distribution. See Methods for details of the validation procedure.

We found that, for both the PS and AA experiments, the accuracy of the estimated thresholds on hold-out tasks is significantly larger than that of randomly extracted thresholds (Fig. 1c; one-tailed Wilcoxon signed-rank tests showed both *P* values <0.001, with effect sizes of *r* = 0.535 and *r* = 0.494 for the PS and AA experiments, respectively). This result indicates that, despite the parsimonious assumptions needed to obtain the simple expression in equation (1), the estimated thresholds exhibit nontrivial out-of-sample predictive power, which points to their ability to predict individual choices and motivates a detailed analysis of their properties. Future studies could use the utility-based framework to develop and validate more sophisticated threshold estimation methods.

### Detection of independent and susceptible individuals

The debate around the shape of the threshold distribution has long stagnated, partly because of the inaccessibility of individual thresholds. The estimated threshold can, therefore, shed light on both the distribution of individual thresholds and its implications for social influence maximization policies. Visual inspections of the threshold distributions for different products reveal that the threshold distribution is strongly product-dependent (Fig. 1d), which is often neglected in simulation-based studies of social influence maximization. For example, in Fig. 1d, the illustrative energy policy exhibits a polarized, bimodal distribution, with most participants being either supportive of the policy (peak at 0) or unsupportive (peak at 1), regardless of the social signal. By contrast, the instant messaging app represented in the same panel shows a more uniform threshold distribution, with a sizeable number of individuals lying in (0, 1) and, therefore, susceptible to different levels of social signal.

Moving from these case studies to the general properties of the threshold distributions across the two contexts, we quantify the proportion of independent adopters in the two experiments, that is, the proportion of estimated thresholds equal to 0 or 1, which denotes a lack of susceptibility to social influence. We find that the proportion of independent adopters was significantly larger in the PS experiment than in the AA experiment (Fig. 1e). This result can be explained in terms of the estimated parameters (Fig. 1f). Assuming that ${\widehat{\gamma }}_{n} > 0$, an adopter is susceptible if and only if $0 < {\widehat{R}}_{ni} < {\widehat{\gamma }}_{n}$. This condition corresponds to a well-defined ‘susceptibility stripe’ in the two-dimensional *γ–R* diagram (Fig. 1f, grey area) where each individual product pair is represented by a dot in the plane spanned by the marginal utility of social signal and resistance. Outside of the susceptibility stripe, the adopter’s choice for the product is independent of the social signal. Significantly more individual product pairs lie within the susceptibility stripe in the AA experiment (61%) than in the PS experiment (20%; Fig. 1f, grey area; two-sample proportion *Z*-test, *P* < 0.001), which explains the lower proportion of independent adopters in the AA experiment (Fig. 1e).

In sum, the estimated thresholds reveal that, in different choice contexts, the number of individuals who choose independently of social signals may differ substantially, which is often neglected in extant social infuence maximization studies. The high proportion of independent adopters in the PS experiments qualitatively agrees with previous research^64^ that found that social norm messages communicating the policies’ support level may be ineffective to stimulate carbon-capturing PS. The two analysed contexts suggest that the number of independent adopters is smaller in contexts where network externalities influence the adoption more strongly. As the main objective of the two experiments is to showcase the feasibility of the threshold estimation method and validate its out-of-sample predictive power, we leave future research to determine whether and how the proportion of susceptible adopters is affected by the framing of the social signal (for example, using a different description of the reference group^65^) and other contextual factors.

### Implications for seeding policies

Do the estimated thresholds challenge what we know about social influence maximization? Here, we consider the seeding problem where a social change practitioner^1^ (for example, a government or an organization) seeks to determine which nodes in a social network to incentivize first to promote the large-scale adoption of a new product or behaviour (hereafter, product)^44,66,67^. This problem represents a specific type of network intervention, which refers to purposeful actions taken by a change practitioner to leverage the structure of social networks in achieving a predefined objective^68,69^. Structure-based seeding policies are agnostic to a potential target’s behavioural characteristics, and they select a set of seeds according to the nodes’ centrality in the relevant social network^66,67^ or to structural properties of the set^70–74^. Conversely, behaviour-based policies (that is, policies that take into account individual behavioural characteristics such as the individual thresholds) assume that some individuals are more amenable to change and, therefore, willing to initiate the adoption process for a new product or behaviour^17,43,45,75^. The underlying hypothesis is that, as low-threshold individuals are willing to adopt a new idea earlier than their peers, they should be engaged early on to create sufficient early momentum and form a critical mass for the desired behavioural change^43,68^. A different way to leverage individual thresholds to initiate change is the recently introduced complex centrality^46^, which is a hybrid method that factors in both network structure and individual thresholds. However, the input thresholds for its calculation are either assumed to be known or extracted from arbitrary distributions, which can lead to suboptimal decisions as shown below. In sum, the estimated thresholds allow us to address the open questions: under which theoretical conditions can behaviour-based (or hybrid) policies outperform structure-based ones?

To address this question, we rely on agent-based spreading simulations based on the threshold model of new product diffusion^8^. In the simulations, given a network composed of *N* nodes, we assign at random *N* agents to the network’s nodes. We analysed the largest components of a subset of the Add Health networks considered by Guilbeault and Centola^46^, which include several independent communities that exhibit high variation in network topological properties (Supplementary Note S2A). We studied the independent diffusion of the 36 products analysed in the AA experiment and a sample of 36 products from those analysed in the PS experiment (see Supplementary Note S2B for the sampling details). For each product, we assume that the agents make adoption choices on the basis of their ground-truth thresholds, which we set to be equal to those estimated from the survey’s participants (Methods). While the ground-truth thresholds used for agents’ adoption decisions are known to the agents, they are not known to the social change practitioner. We assume that the practitioner has been able to survey the nodes of the network with a choice-based conjoint survey and use the collected choice data to estimate the individual thresholds. For our theoretical analysis, as detailed in Supplementary Note S3, we simulate the agents’ answers to the survey on the basis of the parameters estimated from the two experiments, and we use the generated choice data to estimate the individual thresholds (‘estimated thresholds’, hereafter) with the same method used for the experimental data (Methods and Supplementary Note S3). In Supplementary Note S5C, we discuss the robustness of the results in scenarios where only a fraction of the agents can be surveyed.

Under these assumptions, the optimal seeds of behaviour-based policies are determined by the agents’ estimated thresholds. The threshold estimation enables the design of various behaviour-based policies: low-threshold policy (where the focal seed is the lowest threshold node), high neighbourhood susceptibility policy (where the focal seed is the node with the largest number of low-threshold neighbours) and high calibrated complex centrality policy (where the focal seed is the node with the largest complex centrality^46^, calculated using the estimated thresholds as inputs). We used various structure-based policies as baselines, which we labelled according to the metric used to select the focal seed: degree^76^, collective influence^71^, betweenness^77^, closeness^78^ and uncalibrated complex centrality (that is, the nodes’ average complex centrality score over an ensemble of homogeneous threshold values^46^ not estimated from actual choice data; see Supplementary Note S4). We also consider a policy where the focal node is selected at random^17^. We refer to Supplementary Note S4 for all the policies’ implementation. We measured each policy’s performance as number of adopters discounted for the intervention cost, rank them within each configuration (defined by the empirical network being analysed, the product and the realization of the simulated conjoint survey and node assignment) and average these ranks across configurations to yield the mean policy rank (Methods).

**Fig. 2:**
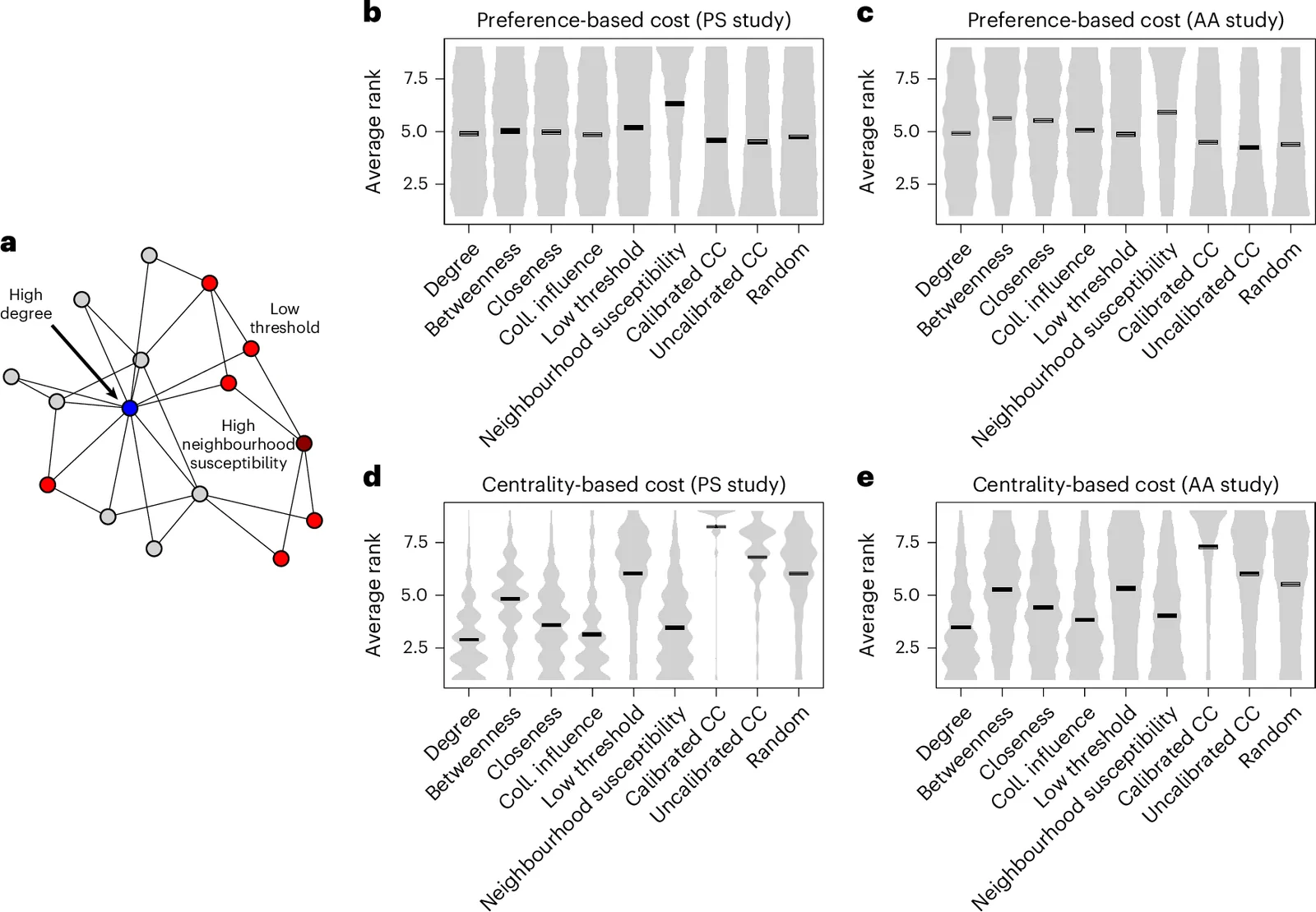
Relative performance of seeding policies. **a**, The nodes selected by different policies in an illustrative network structure: the highest-degree node (blue) has the largest number of connections, but the highest-neighbourhood susceptibility node (dark red) has the largest number of connections to low-threshold nodes (red). **b**,**c**, The relative performance of seeding policies under a preference-based cost structure, measured through the average rank defined in the main text (higher values indicate better performance), for the PS experiment (**b**) and the AA (**c**) experiment. The neighbourhood susceptibility policy, based on the estimated thresholds, significantly outperforms the other policies. **d**,**e**, The relative performance of seeding policies under a centrality-based cost structure for the PS experiment (**d**) and the AA experiment (**e**). The complex centrality policy, based on the estimated thresholds, significantly outperforms the other policies. In **b**–**e**, Coll. influence, collective influence; CC, complex centrality; crossbars represent the means and the 95% confidence intervals over 3,240 runs (Methods). Source data.

We found that, while there is no universally best-performing policy, the estimated thresholds are key to identifying the best-performing seeds. The relative performance of the policies critically depends on the structure of the cost function, but in all the analysed scenarios, the best-performing policy is one that takes into account the estimated thresholds. Specifically, we first consider a cost function such that the cost to successfully initiate the spreading only depends on the seed nodes’ preferences, but it is independent of the nodes’ centrality (preference-based cost; Methods). This scenario is especially relevant to contexts where the seeds mostly adopt for personal motivations, and there is no reason why it would require more effort or monetary incentive to persuade central individuals to early adopt^20^. In these contexts, high-resistance individuals might require stronger incentives to be persuaded to initiate the spreading (for example, more intervention cycles in a field intervention, or higher monetary incentives in marketing contexts; Methods). We find that, in the preference-based cost scenario, the neighbourhood susceptibility policy significantly outperforms all the others (Fig. 2b,c). Differences in performance between the neighbourhood susceptibility and all other policies are statistically significant (*P* < 0.001 for all one-tailed Wilcoxon signed-rank tests, the smallest effect size being *r* = 0.07 in the AA study in the comparison against the betweenness centrality). Through the estimated thresholds, neighbourhood susceptibility allows the identification of social neighbourhoods that are more amenable to change and require a lower cost to successfully initiate the diffusion. Among the remaining policies, two structure-based policies (betweenness and closeness) performed relatively well, yet they underperform with respect to neighbourhood susceptibility. In this cost scenario, both the calibrated and the uncalibrated complex centrality generally underperform the other policies.

The relative performance of the policies changes dramatically as we consider a different cost function, such that the cost to target a node grows with the node’s centrality, independently of the node’s preferences (Methods). This scenario is highly relevant to all those applications where there is a strategic advantage of initiating the diffusion from high-centrality nodes, which is often the case in online influencer marketing and other interventions where central nodes are highly sought-after because of their large follower base and, therefore, are less responsive^79^ and charge higher prices for endorsing new products^80–82^. We find that, in the centrality-dependent cost scenario, the calibrated complex centrality significantly outperforms all the other policies (Fig. 2d,e; one-tailed Wilcoxon signed-rank tests comparing the performance of calibrated complex centrality to each alternative are statistically significant in all cases (*P* < 0.001); the smallest effect size was *r* = 0.37 in the AA study in the comparison with the uncalibrated complex centrality). This is because of its capability to identify low-degree nodes with high spreading capacity for threshold-based diffusion^46^. At the same time, the uncalibrated complex centrality—which neglects the estimated thresholds—fails to reach a similar performance and, for a smaller seed set size, even fails to outperform random seeding (Supplementary Figs. S13 and S14). Therefore, the complex centrality is consistently effective at identifying optimal seeds, but only if estimates of the individual-level thresholds are available to the social change practitioner. Structure-based policies, other than the uncalibrated complex centrality, typically exhibit a lower performance than the random seeding policy. This confirms the argument that, when the higher connectedness of high-degree nodes affects the seeding cost, the social hubs’ spreading ability might not be large enough to justify the extra efforts required to detect and target them^80^. In this scenario, only the calibrated complex centrality leads to consistently higher seeding performance.

## Discussion

To effectively steward collective behaviours in social systems, scholars from diverse disciplines^13,19–22,83^ have suggested the need for integrating individual-level behavioural models with simulations of collective behaviour, but data-driven efforts in this direction remain rare. Our work contributes to such integration by (1) demonstrating that, building on discrete-choice theory, one can estimate individual-level thresholds for behavioural change, which we validate across two different contexts of varying degrees of social influence, and (2) leveraging the estimated thresholds to inform seeding policies aimed at initiating social change.

At the individual level, our experimental results indicate that, once the thresholds are estimated, individual choices in out-of-sample tasks exhibit nontrivial predictability. This finding not only provides a validation of the estimated thresholds but might also inspire future studies aimed at improving the predictive power. Further, the estimated thresholds allowed us to disentangle an experimental choice context where most individuals choose independently of social signals from one where most individuals are susceptible to social influence.

Future empirical studies could leverage the proposed method for more refined tests of social contagion theories. In our work, all participants were exposed to the percentage of friends who selected each alternative, which necessarily leaves out the variety of social cues encountered in field applications^84^. This suggests two natural directions for generalizing our findings, related to the characteristics of the decision-maker and the received social cues, respectively. At the decision-maker level, future studies could examine how the size of an individual’s personal network and their individual role within a community affect their thresholds. As for the social cues, future studies could examine whether different types of social cues—for example, coming from more similar individuals or individuals with different roles in the community—affect individual responses. Addressing these factors in the field might require more sophisticated conjoint designs, potentially using vignettes^65^. We envision that these efforts could systematically identify which influence mechanisms and contexts cause social signals to play (or not play) a critical role in shaping individual choices, and what the best policy response could be.

In this work, to illustrate how the thresholds could be estimated from empirical data, we collected data using a conjoint design owing to its simplicity for eliciting stated choices that mimic real-world decisions. For real-world interventions informed by our work, practitioners may face two potential barriers. It is beyond the scope of this work to fully address them, but we offer a few comments on how future research could overcome them.

The first concern is whether hypothetical decisions in the conjoint survey can predict responses to social signals in a real-world intervention. Prior evidence shows that, at least in some contexts, insights from hypothetical conjoint studies can match remarkably well those from behavioural data^85^. In general, potential biases induced by the hypothetical nature of conjoint experiments can be alleviated by various means—for example, by using vignettes with fictitious characters to reduce social desirability bias^65^, or through incentive-aligned designs^52^ where responses are consequential and, therefore, participants have a direct incentive to reveal their true preferences. Broader concerns about the external validity of laboratory experiments can be mitigated by using observational data—if available—instead of or as a complement to experimental data. In this respect, the individual-level choice framework adopted here is not limited to data collected from conjoint experiments, as it can in principle be adapted to any setting where data on stated choices is available.

The second concern is feasibility, as conjoint experiments might be too costly to conduct at scale. Of course, the feasibility of collecting the necessary data depends on the size of the target population of the social change intervention. In many cases (for example, rural communities^67,86^ and school classes^87–89^), the target population is small enough that it is feasible to survey it entirely (or to survey a sizeable portion of it). When the population is too large to be surveyed, one can survey small samples to estimate the thresholds at the group level, or rely on observational data which are already available in some contexts, for example, for promoting sustainable behaviours^90^ or informing public health interventions^91^. In scenarios where rich observational data are available at the individual level, one could adapt techniques to estimate peer effects from observational data^92,93^ to the threshold measurement. Adapting the utility-based framework to the specifics of different data sources is a promising avenue for further research.

Moving from the individual to the collective level, our findings reveal the circumstances under which seeding policies that incorporate behavioural models can outperform traditional, structure-based policies. For example, the recently introduced complex centrality^46^ identifies effective seeds only when the change practitioner has been able to collect individual threshold data, and the cost of successfully targeting a node grows with their network centrality. The simpler neighbourhood susceptibility policy—relying on the estimated number of susceptible neighbours a given node has—only outperforms the other methods when the cost of successfully targeting a node depends on their intrinsic preferences. Estimating the threshold distribution through the proposed methodology and validating the resulting predictions in real-world network diffusion processes is an important direction for future research.

The boundaries to the effectiveness of the utility-based framework adopted here also deserve to be further investigated in future research. While we assumed a threshold-based diffusion model, aligned with the main paradigm in the social contagion literature^23,24,43^, we show in Supplementary Note S6 that a utility-based framework under different assumptions than those adopted above still recovers interpretable social spreading models—for example, the traditional Bass diffusion^94^ and logistic diffusion models^95^—which could be investigated in future empirical research. Further, we assumed that the timescale that governs the diffusion of the new product or behaviour is considerably shorter than the timescale that governs changes in individual-level preferences (that is, thresholds) and network structure. This assumption is in line with state-of-the-art social influence maximization works^17,18,23,24^. In real complex social systems, however, stronger feedback loops may exist: collective diffusion outcomes, top-down interventions, and interactions between multiple diffusion processes can alter individual-level preferences. In some cases, preference changes could occur at similar timescales as diffusion processes^96^. Adapting the utility-based framework to these more complex scenarios and determining the theoretical limits to the proposed approach remain fascinating challenges for future studies.

## Methods

### Threshold estimation

We describe here the threshold estimation method from equation (1). Consider an individual *n* who needs to select a product from *I* alternatives. Each alternative *i* ∈ {1, …, *I*} brings individual *n* a certain utility *U*_*ni*_. In line with discrete-choice theory^3^, we assume that individual *n* chooses alternative *i* if and only if *U*_*ni*_ > *U*_*nj*_, ∀ *j* ≠ *i*. The utility can be decomposed into an observable, systematic component (${U}_{ni}^{* }$) and a random, unobserved component (*ϵ*_*ni*_) such that ${U}_{ni}={U}_{ni}^{* }+{\epsilon }_{ni}$. The probability that individual *n* chooses alternative *i* over *j* is $\,{\rm{Prob}}({U}_{ni} > {U}_{nj})={\rm{Prob}}\,({\epsilon }_{nj}-{\epsilon }_{ni} < {U}_{ni}^{* }-{U}_{nj}^{* })$. As in the main text, we assume—as is typical in discrete-choice modelling^3^—that the attribute utility is linear and separable in the attributes. Following the notation in the main text, the systematic utility of individual *n* from adopting product *i* is expressed as

2 $${U}_{ni}^{* }=\mathop{\sum }\limits_{k=1}^{K}{\beta }_{nk}{x}_{ki}+{\gamma }_{n}{s}_{ni}.$$

Under the assumption that *ϵ*_*ni*_ is independent and identically Gumbel distributed, the model parameters (*β*_*nk*_, *γ*_*n*_) can be estimated from observed choices (for example, in a choice-based conjoint experiment^61^) using a HB algorithm^60^. In this model, the probability of individual *n* to select an item *i* from a choice set is expressed as a multinomial logit whose parameters are estimated for each individual using Markov chain Monte Carlo methods. In this work, we used the R package ChoiceModelR^97^ for the HB estimation. See Supplementary Note S1 for more details on the estimation method used in the two experiments. The estimation yields individual-level estimates, and thus allows the calculation of the adoption threshold for every individual and every product.

Inevitably, the estimation results are to some extent model-dependent, as they reflect the assumptions embedded in the choice model specification and the HB estimation. An additive and separable model rules out interaction effects, which can be important in some real-world applications. Although interaction models can still yield a simple analytic threshold (Supplementary Note S6B), estimating models with a large number of interaction terms can pose substantial challenges owing to model complexity^98^. In applications where interactions are essential, this concern may be partly alleviated by restricting interactions to a small set of theory-motivated effects^61^, and by adopting conjoint designs and estimation techniques tailored to identify them^61,99^. More generally, utility-based contagion models can be extended in several directions, for example, by including negative externalities^100^ or anticonformist agents^101^ to allow for downward thresholds (an agent abandons a given alternative when a threshold fraction of their social contacts have already adopted it) and even multi-threshold responses.

For the HB estimation, the modelling assumptions are embedded in the Bayesian priors, whose specification can influence the resulting estimates. This dependence diminishes as the number of choice tasks per participant increases, since a broad range of priors will result in similar inferences in large datasets^102^. However, when the number of choice tasks per individual is small relative to the number of parameters to be estimated, the choice of priors might have an impact. Supplementary Note S3C,D shows that our main results are robust across four model specifications that differ in the assumptions on the prior distributions made in the HB model. With the exception of the extreme case of an uninformative prior, the choice of the prior distribution has a marginal impact on the accuracy of the partworth utility estimates (Supplementary Fig. S9A,D). Further, except for the uninformative prior, the accuracy of adoption threshold estimates and their correlation with the true thresholds remain high and consistent across prior specifications (Supplementary Fig. S9B,C,E,F). As a result, our conclusions on the relative performance of behaviour-based seeding strategies are unaffected by prior choice (Supplementary Figs. S22 and S23). Therefore, while the estimated adoption thresholds should not be interpreted as model-free estimates, our sensitivity analysis shows that they remain robust across specifications and can reliably inform the design of social change interventions.

### Validation of estimated thresholds

We aim to assess the predictive power of the individual thresholds estimated from choice-based conjoint data. The core idea is to estimate the thresholds on the basis of a training set, and quantify how many times, in the validation set, the participants make predictable choices, that is, choices that do not contradict their threshold values. For each task in the survey, we fit the HB model (see previous section) on a training set consisting of all tasks except one hold-out task, and use the fitted model to predict the choices in the validation set consisting of the hold-out task. We then calculate the average number of predictable choices over all the training–validation splits. Specifically, for a given choice task, we quantify the estimated appeal of product alternative *i* to participant *n* as ${\widehat{{\Delta }}}_{ni}:={s}_{i}-{\widehat{\tau }}_{ni}$, where *s*_*i*_ denotes the social signal displayed for alternative *i*, and ${\widehat{\tau }}_{ni}$ denotes the estimated threshold (based on the training set) of participant *n* for alternative *i*. The appeal of the default alternative (nonadoption) is zero, as by definition, any product alternative would be more appealing than the default if the respective social signal would exceed the corresponding estimated threshold.

Formally, given that participant *n* selected alternative i^*^ in task ${{\mathcal{Z}}}_{z}$ (*z* ∈ {1, …, *Z*}, with *Z* being the number of choice tasks), we say that, compared with a different alternative $i\in {{\mathcal{Z}}}_{z}$, i^*^ is a predictable choice on the basis of *n*’s estimated thresholds if ${\widehat{\varDelta }}_{n{i}^{* }} > {\widehat{\varDelta }}_{ni}$, where ${\widehat{\varDelta }}_{ni}$ denotes the estimated appeal of alternative *i* to participant *n*. As each task ${{\mathcal{Z}}}_{z}$ has *I* = 4 alternatives (three products and one default option), we can measure the degree of predictability of *n*’s choice for task ${{\mathcal{Z}}}_{z}$ as

3 $${a}_{nz}=\frac{1}{I-1}\mathop{\sum }\limits_{i\in {{\mathcal{Z}}}_{z}\backslash \{{i}^{* }\}}\Theta ({\widehat{{\Delta }}}_{n{i}^{* }}-{\widehat{{\Delta }}}_{ni}),$$

where the sum runs over the *I* − 1 alternatives in task ${{\mathcal{Z}}}_{z}$ that were not selected by *n*; *Θ*(*x*) = 1 if *x* > 0, *Θ*(*x*) = 0 otherwise. Basically, an observed choice is perfectly predicted by the *n*’s estimated thresholds if none of its alternatives were more appealing according to the estimated thresholds. Pooling the predictability levels over all participants and over all training–validation splits (and considering that there is only one hold-out task in each validation set), we define the accuracy of the estimated thresholds as

4 $$A=\frac{1}{NZ}\mathop{\sum }\limits_{n=1}^{N}\mathop{\sum }\limits_{z=1}^{Z}{a}_{nz},$$

where *N* denotes the number of participants and *Z* denotes the number of choice tasks in the experiment (here equal to the number of training–validation splits). Note that the accuracy function can be connected with the choice probabilities of a logit choice model in the limit of a strong marginal utility of a social signal: under the logit model (Supplementary Note S6B), in the limit ∣*γ*∣ ≫ 1, we see that ${P}_{n{i}^{* }}/{P}_{ni}\simeq \varTheta ({\widehat{\varDelta }}_{n{i}^{* }}-{\widehat{\varDelta }}_{ni})$.

### Data-driven spreading simulations

We consider threshold-based spreading simulations where the agents’ thresholds are set on the basis of the experimental results. For the simulation, we set the the ground-truth threshold of a given agent for a given product to the corresponding value estimated in the choice experiment for a randomly selected participant (sampled with or without replacement conditional on whether the number of nodes occupied by the agents, *N*, is larger or smaller, respectively, than the number of participants, *N*_R_). We additionally assume that the hypothetical decision-maker has been able to survey the nodes of the network with a choice-based conjoint experiment. We simulate the answers of the agents in the conjoint experiment on the basis of the individual-level utilities estimated in the choice experiments. We then proceed with the proposed threshold estimation procedure to estimate the thresholds (Supplementary Note S3). We denote by *τ*_*ni*_ and ${\widehat{\tau }}_{ni}$ the ground-truth and estimated threshold, respectively, of node *n* for product *i*. We simulate diffusion processes that unfold according to the same fractional threshold model studied in ref. ^46^ using the ground-truth thresholds {*τ*_*ni*_}.

We run the diffusion processes throughout the largest connected components of 18 empirical Add Health networks sampled from those analysed by Guilbeault and Centola^46^ (see Supplementary Note S2A for the sampling procedure). For each network structure, we measure the nodes’ centralities according to the degree, betweenness, closeness and collective influence (Supplementary Note S4A). Further, for each network structure, product, realization of the simulated conjoint survey and agent assignment, we measure the behaviour-based metrics defined in Supplementary Note S4 using the estimated thresholds $\{{\widehat{\tau }}_{ni}\}$ as input. Motivated by the effectiveness of clustered seeding for effective threshold-based diffusion^46^, for each policy, we adopt a clustered seeding protocol where we target one focal seed selected by the metric and a subset of their closest neighbours such that ⌊*zN*⌋ of the nodes are initially active (if the focal seed node has not enough neighbours to reach this proportion of seeds, a recursive search on the higher-order neighbours is performed^46^; we also set a minimum seed set size of four nodes). In the main text, we set *z* = 0.025; we show in Supplementary Note S5B that our conclusions are mostly robust with respect to other small values of *z*.

In the preference-based cost scenario, the cost of successfully seeding product *i* with the selected seed set, ${\mathcal{S}}$, is defined as

5 $${C}_{i}({\mathcal{S}})={c}_{0}+\mathop{\sum }\limits_{n\in {\mathcal{S}}:{R}_{ni} > 0}{R}_{ni},$$

where ${R}_{ni}={U}_{n}^{(0)}-{U}_{ni}^{({\rm{A}})}$ and *c*_0_ = 1 is a fixed implementation cost per intervention. This cost function embodies the idea that beyond a fixed implementation cost, *c*_0_, it is more costly to successfully target individuals with higher resistance to the product, which can be motivated through a simple choice-theory argument (Supplementary Note S6B). In the centrality-based cost scenario, the seeding cost is defined as

6 $${C}_{i}({\mathcal{S}})={c}_{0}+\mathop{\sum }\limits_{n\in {\mathcal{S}}}{d}_{n},$$

that is, the cost of successfully seeding a node *n* grows with their degree *d*_*n*_(refs. ^79,81,82^). The performance of the seeding policy that targets seed set ${\mathcal{S}}$, ${P}_{i}({\mathcal{S}})$, is assumed to be the final number of adopters, ${A}_{i}({\mathcal{S}})$, discounted for the intervention’s cost: ${P}_{i}({\mathcal{S}})={A}_{i}({\mathcal{S}})/{C}_{i}({\mathcal{S}})$.

To assess the relative performance of the examined seeding policies, for each analysed configuration ${\mathcal{C}}$, we rank the nine policies by their simulated performance. Here a configuration ${\mathcal{C}}$ is identified by the network being analysed, the product that spreads through the network, the particular realization of the simulated conjoint survey performed by the change practitioner to estimate the thresholds and the agents’ random assignment to the network’s nodes. For a given seeding policy, considering 18 empirical networks, 36 products and 5 realizations of the simulated conjoint survey and random node assignment for the simulated participants (Supplementary Notes S2 and S3A), we ran 3,240 independent simulations. Therefore, for each configuration ${\mathcal{C}}$, each method *m* is assigned to a rank variable ${r}_{m}({\mathcal{C}})$, with higher values indicating better performance (in case of ties, we assigned the average of the ranks that would have been assigned without ties). We compared the methods’ average rank across all configurations

7 $${\bar{r}}_{m}=\frac{1}{| {\mathcal{C}}| }\mathop{\sum }\limits_{{\mathcal{C}}}{r}_{m}({\mathcal{C}}),$$

as done in Friedman’s statistical test^103^. We also considered an alternative metric, where for each configuration ${\mathcal{C}}$, each method *m* is assigned to a performance variable that quantifies the method’s performance compared with the best-performing method^104^, ${s}_{m}({\mathcal{C}})={P}_{m}({\mathcal{C}})/{\max }_{{m}^{{\prime} }}\{{P}_{{m}^{{\prime} }}({\mathcal{C}})\}$. Again, we averaged this variable over all configurations. For the best-performing policies in the preference-based cost scenario (that is, neighbourhood susceptibility) and the centrality-based cost scenario (that is, calibrated complex centrality), we performed pairwise one-tailed Wilcoxon tests^46^ between the ranks of the best-performing policy and those of all the other policies, to ensure that the differences were statistically significant (*P* < 0.05).

### Institutional review board authorization

The research complies with all relevant ethical regulations and was approved by the University of Zurich Review Board (OEC IRB 2020-081 from 3 November 2020). Before the experiments, all participants provided electronic informed consent. Participants in the PS and AA studies (respectively: *N* = 296 and *N* = 300, 49% and 54% female and modal age groups: 55–64 and 35–44 years) were compensated €1.80 and €1.65, respectively.

### Reporting summary

Further information on research design is available in the Nature Portfolio Reporting Summary linked to this article.

## Supplementary information

Supplementary Information Supplementary Notes S1–S6, Figs. S1–S23 and Tables S1–S5.

Reporting Summary

Peer Review File

## Source data

Source Data Fig. 1c Accuracy of the two methods (estimated and random thresholds) in predicting the out-of-sample choices of every participant, for both studies. Source Data Fig. 1d Participants’ estimated threshold values for the two selected products. Source Data Fig. 1e Number of independent products for each analysed product, for both studies. Source Data Fig. 1f Resistance and marginal utility of social signal for all participants and products, for both studies. Source Data Fig. 2b Results of simulations calibrated with the PS study results in the preference-based cost scenario. The file reports the rank score of each seeding policy for each configuration, defined as the empirical graph being analysed, the realization of the conjoint survey (‘setup’ column) and the product that is spreading. Source Data Fig. 2c Results of simulations calibrated with the AA study results in the preference-based cost scenario. The file reports the rank score of each seeding policy for each configuration, defined as the empirical graph being analysed, the realization of the conjoint survey (‘setup’ column) and the product that is spreading. Source Data Fig. 2d Results of simulations calibrated with the PS study results in the centrality-based cost scenario. The file reports the rank score of each seeding policy for each configuration, defined as the empirical graph being analysed, the realization of the conjoint survey (‘setup’ column) and the product that is spreading. Source Data Fig. 2e Results of simulations calibrated with the AA study results in the centrality-based cost scenario. The file reports the rank score of each seeding policy for each configuration, defined as the empirical graph being analysed, the realization of the conjoint survey (‘setup’ column) and the product that is spreading.

## Data Availability

The data collected in this study are available via Zenodo at https://doi.org/10.5281/zenodo.17841193. Source data are provided with this paper.

## Code availability

The code used to obtain the findings of this study is available via Zenodo at https://doi.org/10.5281/zenodo.17841193.
