# Supplementary material for: Integrating behavioural experimental findings into dynamical models to inform social change interventions
Source: Nat Hum Behav. 2026 Mar 16;10(5):873–83. doi: 10.1038/s41562-026-02417-4 (PMC13661244; doi:10.1038/s41562-026-02417-4)
Supplement: Supplementary file 1 [file 41562_2026_2417_MOESM1_ESM.pdf]

nature human behaviour

Supplementary information https://doi.org/10.1038/s41562-026-02417-4

Integrating behavioural experimental findings into dynamical models to inform social change interventions

In the format provided by the authors and unedited

CONTENTS

S1. Choice-based conjoint experiments 2
A. Brief introduction to choice-based conjoint experiments 2
B. The policy support study (PS) 2
C. The app adoption study (AA) 7

S2. Data for the data-driven simulations 11
A. Social network data 11
B. Product sampling 11

S3. Simulated conjoint surveys 11
A. Survey simulation 11
B. Parameter recovery 12
C. Sensitivity to prior specification 14
D. Understanding the effect of partworth modality on estimation accuracy 15

S4. Seeding policies 19
A. Structure-based seeding policies 19
B. Behavior-based seeding policies 19

S5. Additional results for seeding policies 21
A. Results for the ratio-to-best metric 21
B. Results for different seed set sizes 22
C. Results in scenarios with partial threshold data 26
D. Effect of prior specification on the identification of effective seeds 31

S6. Recovering social spreading models from choice theory 33
A. General framework 33
B. High clarity regime: Threshold model 34
C. Low clarity regime: Bass model 36

S1. CHOICE-BASED CONJOINT EXPERIMENTS

A. Brief introduction to choice-based conjoint experiments

Conjoint experiments are a widely-used technique [1] to measure how the total evaluation of a product or behaviour (further referred as product) is related to the aspects describing the product (further referred as product attributes). Choice-based conjoint experiments (CBC) are a specific type of conjoint experiments where the product evaluation is done in terms of a discrete choice. Specifically, participants in CBC experiments are faced with a number of choice tasks. In each choice task, participants are presented a number of products (i.e, choice alternatives) which differ along several dimensions (i.e., product attributes) and are asked which one they would choose (if any) in a given (often hypothetical) situation. From observing such choices, it is then possible to infer which product attributes individuals value most in making the decision. This builds on the premise that when making the choice, participants trade-off different aspects of the choice alternatives.

The theoretical foundation for CBC lies in the random utility theory [2]. In such models, an individual is faced with a number of choice alternatives in a choice set, each bringing her a certain utility. The individual will choose the alternative with the highest utility. While the utility of each alternative is known to the individual, it is not known to the researcher. Therefore, from the researcher's point of view, an individual's (random) utility can be decomposed into a deterministic part and a random component [3]. The deterministic part is a function (often a linear combination) of the utility corresponding to each product attribute (i.e., partworth utilities or simply attribute utilities). The random component is unknown and requires some assumptions about its distribution. These assumptions determine which model will be used to describe the probability that a product is chosen. The most used model is the multinomial logit, which assumes the random component follows an extreme value distribution [1].

The Hierarchical Bayes (HB) model [4] (used in this article) is a variation of the multinomial logit that yields individual-level estimates of the attribute utilities (i.e., one estimate for each individual and each level of any product attribute). Based on the individual level estimates, it is then possible to calculate the total utility of a product for an individual. The HB model assumes that the individual partworth utilities follow the same distribution for all the individuals in the sample, with the distribution's parameters drawn from a population distribution. To estimate the partworth utilities, it first assumes an initial value of the parameters describing the distribution (i.e., prior belief) and then updates it based on data in a Bayesian manner. As the resulting equations cannot be solved analytically, the estimation is done using Markov Chain Monte Carlo (MCMC) methods [1, 4]. We refer the interested reader to ref. [1] for an applied introduction into conjoint analysis and to ref. [3] for a comprehensive read which includes extensive details into how various models are formulated and estimated.

B. The policy support study (PS)

Study design and data. We recruited 296 US participants (300 participants in total, but 4 participants have been excluded for not completing the survey) from a demographically (i.e., gender and ethnicity) representative US sample on Prolific to take part in a choice-based conjoint survey on support for carbon capturing policies. No statistical methods were used to pre-determine the sample size but our sample size is similar to those reported in previous publications (e.g., ref. [5]). Participants were told to imagine a carbon capture and storage policy might be implemented in their state and they need to select which alternative from a choice set they would endorse. Each choice set contained three policies and a none option. The policies presented were described by five attributes (policy type, policy costs, beginning of the policy implementation, required distance to residential areas and organisation endorsing the policy) with four levels each, closely following Pianta et al. [6]. Additionally, we included a sixth attribute representing the (hypothetical) percentage of one's friends who endorse the policy. Participants were presented with a total of 15 choice tasks, each participant receiving a different set of choice tasks (generated by the Sawtooth software using the balanced overlap method [7]). An overview of the attributes and levels used in the conjoint design can be seen in Table S1. A sample of the conjoint survey administered to the participants is attached to this Supplementary Material.

Estimation. We estimated the individual partworth coefficients using the Hierarchical Bayes [4] algorithm. We used the function `choicemodelr` from the R package `ChoiceModelR` [8] with 30'000 MCMC iterations (10'000 for burn in and the remaining 20'000 for parameter estimation, from each every tenth draw was retained). We considered the attribute *Percentage of friends who endorse the policy* as numeric and the remaining attributes (including the none option) as categorical, for which we used effects coding. Note that in effects coding all levels of a variable represent deviations from the overall mean (as opposed to a specific reference level when using dummy coding) with the constraint that all levels sum to zero. For the none option, the effects coding implies we have one level for choosing the status quo (`none=True`) and one level for choosing any of the alternatives in the choice set (`none=False`). We note

Attributes	Levels				
Policy type	Ban	Subsidies	Tax		
Policy costs	\$4	\$9	\$14	\$19	
Begining of policy implementation	2025	2035	2045	2055	
Required distance to residential areas	2 miles	5 miles	10 miles	50 miles	
Policy endorsement by	Carbon Capture Coalition	Greenpeace	Democratic Party	Republican Party	
% of your friends who endorse the policy scenario	1%	23%	45%	76%	98%

TABLE S1. **Attributes and levels used in the Carbon Capturing Policy Study.**

that in computing the utility of an alternative in the choice set, the value of `none=False` needs to be added to (the sum of) the utilities of the attributes describing the alternative. Thus individual n's utility from adopting product (or behavior) i is $U_{ni} = U_{ni}^{(A)} + U_{ni}^{(S)} + U_n^{(\bar{0})}$, where $U_n^{(\bar{0})}$ is the utility corresponding to `none=False`. As this is just an artifact of the coding system used in estimation, we referred everywhere in the text to U_{ni} as $U_{ni} = U_{ni}^{(A)} + U_{ni}^{(S)}$, which correspond to a dummy coding of the none option. The HB algorithm does not directly result in point estimates (single values of a parameter) but rather in a distribution of values for each parameter (one value being generated in each MCMC draw used for parameter estimation). Therefore, we calculated the parameter estimates as the sample mean of the distribution of the MCMC draws. We input the resulting estimated parameters $(\hat{\beta}_n, \hat{\gamma_n}, \hat{U}_n^{(0)})$ into Eq 1 in main text to obtain the estimated threshold for product i.

Model quality. First, we measured the reliability of the conjoint data by including two fixed choice tasks (on positions 5 and 10) that were identical within respondents, and measured how many respondents gave the exact same answer to both tasks [5]. We found the answers to the two questions were the same for 77% of the respondents, which is substantially larger than the expected fraction (25%) in a baseline model where individuals make decisions at random.

Second, we evaluated how well the model predicts choices out of sample. To this end, we split the data into a held-out test set consisting of one choice task and a training set consisting of the remaining choice tasks[1]. We fitted the model only on the training set, predicted the choices in the test set, and compared the predictions against the observed choices. We repeated this process for all choice tasks. We found an average out of sample accuracy rate of 68%, which is significantly larger than the accuracy rate of a baseline model making choices at random (25%, as each tasks consists of 3 policy alternatives + a none option).

Results. We asses the impact of each attribute on the respondents' choices by measuring the attributes' relative importance. To measure the attribute importance for an individual, we measure for each attribute a, the difference between the maximum and minimum marginal utility across all levels of a. We normalize the obtained attribute importance values so that they sum to one for each individual. To obtain the results in Table S2, we subsequently average the obtained individual attribute importance values over all individuals [9]. We found the attribute with the highest relative importance is the beginning of policy implementation, followed by policy costs, required distance to residential areas, organisation endorsing the policy, percentage of friends endorsing the policy, and policy type (see Table S2). On average, cheaper, stringent (ban) policies that start soon and are further away from the residential area are more appealing than expensive, lax policies that start late and close to the residential area (see Fig. S1). The ranking of the attribute level utilities within the same attribute are largely consistent with the study we draw from [6], the only difference being in the ranking of organisations endorsing the policy. We observe that 291 out of the 296 valid respondents have a positive partworth utility of the social signal (with the remaining 5 having a negative value). We note that an interpretation of the individual-level threshold is only compatible with the social contagion literature if the partworth utility of the social signal is not negative, which is true for almost all respondents. We remove from our analysis the 5 respondents with a negative partworth utility of the social signal, and leave the study of these special cases to further research. We measure the individual-level thresholds using the methodology described in the Methods section of the article for the 36 sampled products (see Supplementary Note S2 B) and calculate the average threshold per product. The results can be seen in Figure S2.

Linearity of the social signal. To test the plausibility of assuming a linear coefficient of the social signal, we re-estimate the model treating the *percentage of friends who endorse the policy scenario* as categorical. Fig. S3 shows that the average utility of the social signal increases with the social signal for all levels but the last one (98%), which exhibits a similar utility to the previous value (76%) (Pearson's $r(1478) = 0.77$, $p < 0.001$, 95% CI=$[0.75, 0.79]$).

---

[1] To avoid data leakage (i.e., information from the test set leaking into the training set), when the held-out test set included one of the two fixed choice tasks, we removed the other from the training data. As there are two identical fixed tasks, removing one of them is essential to avoid that the same task is present in both the training and test sets.

Variable	Average Importances	Standard Deviation
Beginning of policy implementation	0.2084293	0.14176090
Policy costs	0.1743773	0.11072475
Required distance to residential areas	0.1620187	0.09583969
Policy endorsement by	0.1606038	0.08421775
% of your friends who endorse the policy scenario	0.1495264	0.08918514
Policy type	0.1450446	0.09645266

TABLE S2. **Average variable importance in the Policy Support Study**. The most important is beginning of policy implementation, followed by policy costs, required distance to residential areas, organisations who endorses the policy scenario, percentage of friends who endorse the policy scenario and policy type.

Give the high positive correlation and that the effect is largely monotonic, for simplicity, we decided to use a linear coefficient in main text. Note that in principle, as long as the effect of social signal on the adoption probability is monotonously increasing, the threshold estimation is still feasible even in scenarios where the linearity assumption is not supported by the data – e.g., by adapting Eq. (1) in main text to a general nonlinear functional form for the social utility[2] or by treating the social signal as categorical and adapting the interpolation procedure in ref. [5] to accommodate a nonparametric representation of the social utility.

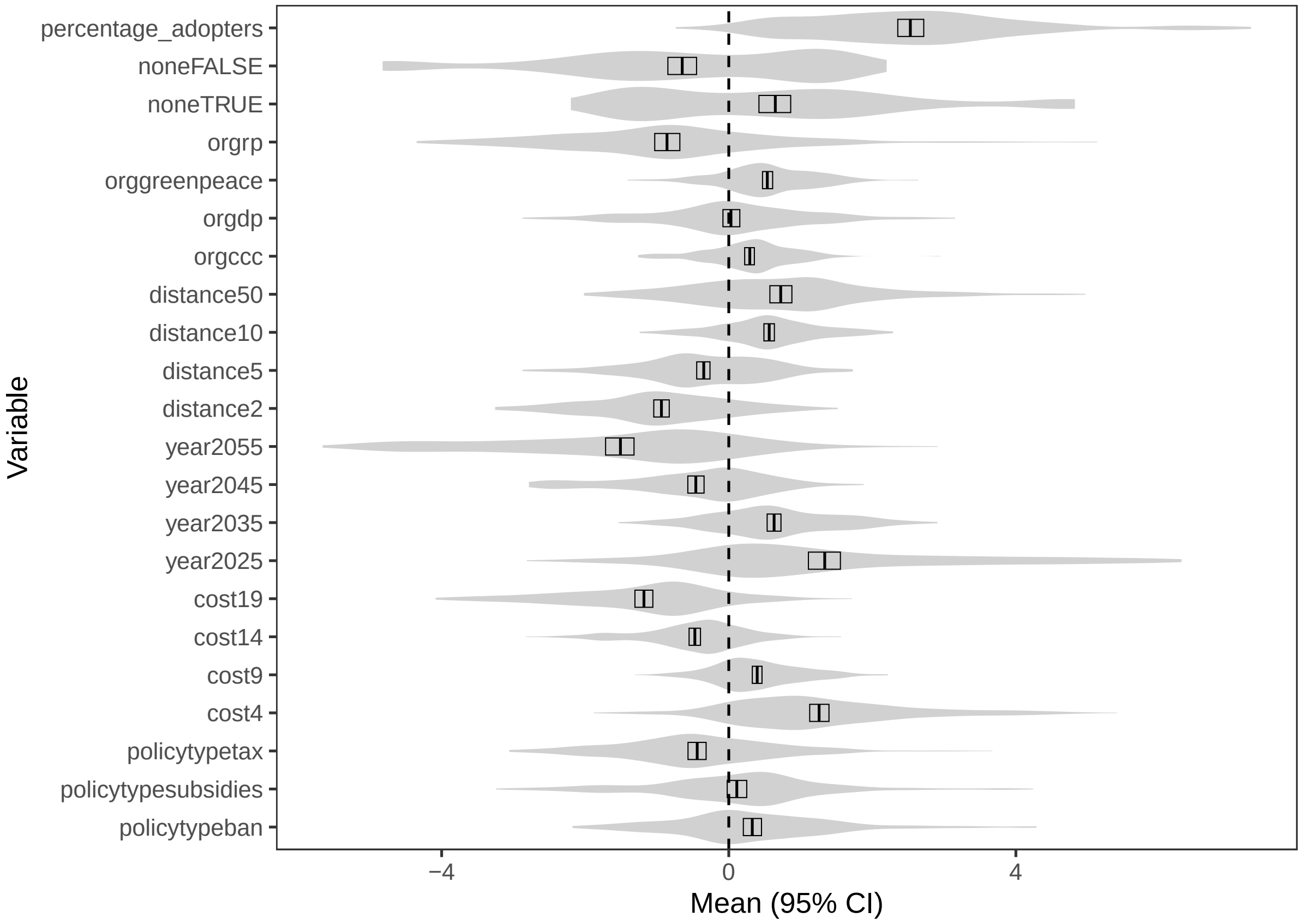

Fig. S1. **Average utilities across respondents in the PS Study.** The y axis contains all attributes and levels. The x axis shows the distribution of the marginal utility of each attribute level together with the mean and 95 % confidence interval (crossbars), based on $N = 296$ observations. On average, the percentage of friends endorsing the policy (labeled as *percentage_adopters*) has a positive partworth utility.

[2] For example, if $U_{ni}^{(S)} = \phi_{\gamma_n}(s_{ni})$, where $\phi_{\gamma_n}(x)$ is a monotonically increasing function in $[0, 1]$ characterized by parameter γ_n, one readily obtains $\tau_{ni} = \phi_{\gamma_n}^{-1}(R_{ni})$.

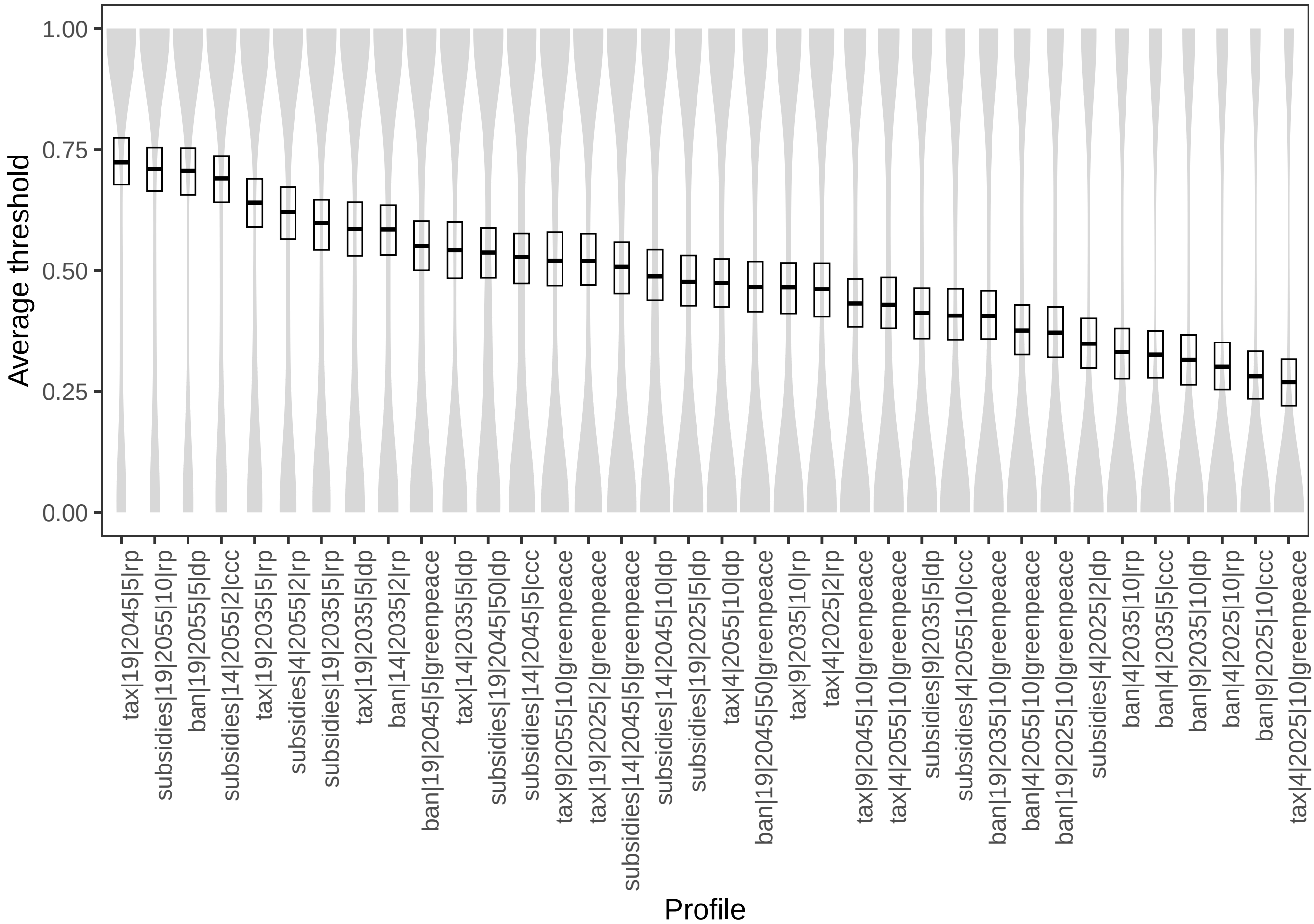

Fig. S2. **Threshold distribution across individuals (y axis) for each policy (x axis) in the PS Study**. Crossbars represent the mean together with the 95% confidence interval based on $N = 291$ observations. The policies are sorted by the mean threshold. We observe that some policies have a lower mean threshold, which implies that they are more attractive to the respondents.

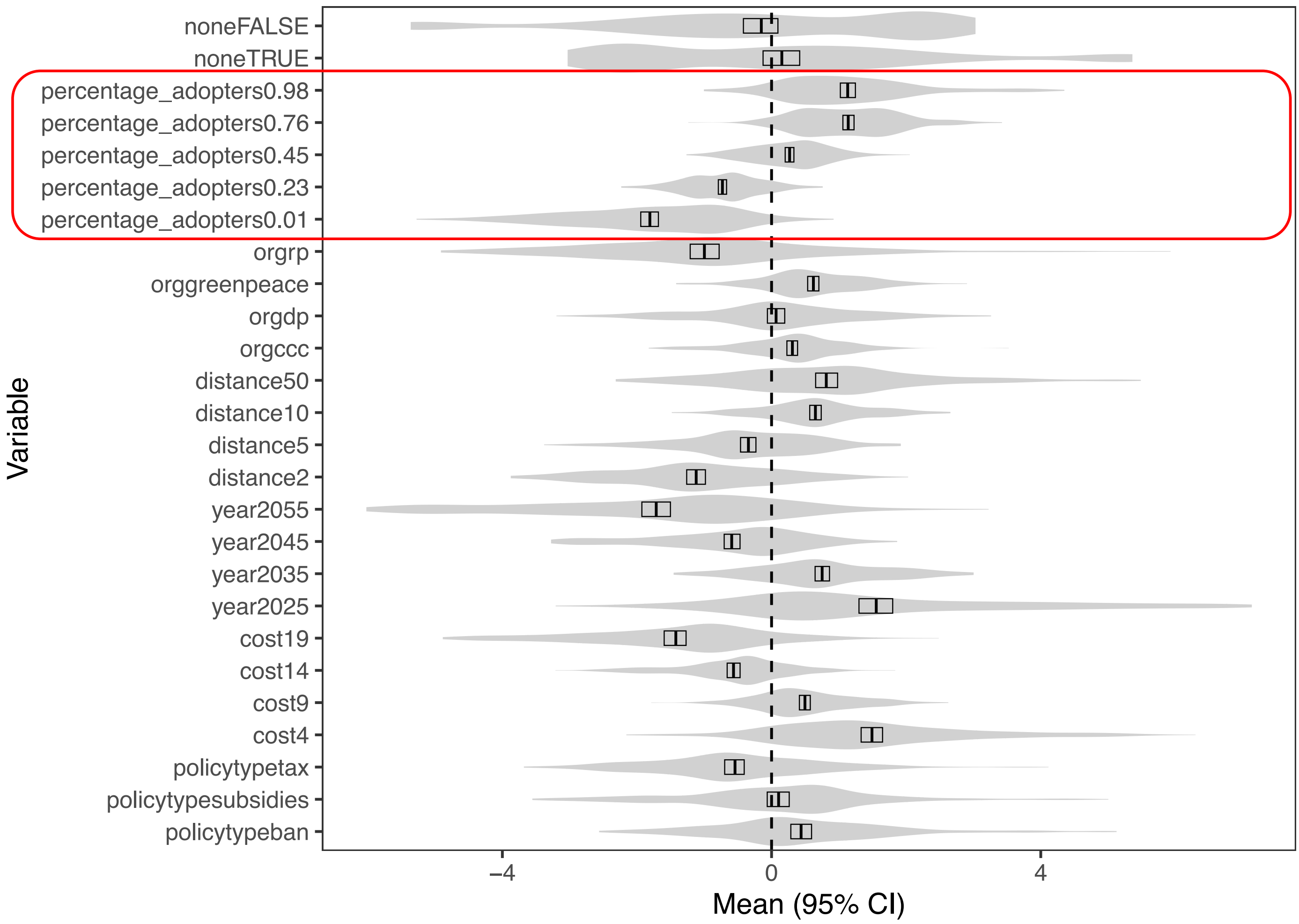

Fig. S3. **Average utilities across respondents treating the percentage of adopters as categorical variable (PS Study).** The y axis contains all attributes and levels. The x axis shows the distribution of the marginal utility of each attribute level together with the mean and 95 % confidence interval (crossbars) based on $N = 296$ observations. On average, the utility of the percentage of adopters increases across the first three levels and plateaus at the highest level, where utilities for the last two levels are comparable (red rectangle).

Attribute	Level				
Accessibility	Mobile	Web			
Authentication	Simple	Two-factor	Multi-factor		
Customisation level	Low	Medium	High		
Video calls	Multi-person	One-to-one			
% of your friends already using the app	1%	23%	45%	76%	98%

TABLE S3. **Attributes and levels used in the App Adoption Study.**

C. The app adoption study (AA)

Study design and data. In this study, we surveyed respondents about their use of a new instant messaging applications. The setup was identical to the PS study. We recruited 300 US participants from Prolific (54% female). The (fictive) messaging apps were described by four attributes (accessibility, authentication, customisation level and video call support) with two to three levels each. Similarly to the PS Study, we included a fifth attribute representing the (hypothetical) percentage of one's friends who are already using the app. Participants were presented with a total of 14 choice tasks, each participant receiving a different set of choice tasks (generated by the Sawtooth software using the balanced overlap method [7]). An overview of the attributes and levels used in the conjoint design can be seen in Table S1. A sample of the conjoint survey administered to the participants is attached to this Supplementary Material.

Estimation. We used again Hierarchical Bayes with 30'000 MCMC iterations, the first 10'000 for burn in and the remaining 20'000 for parameter estimation (from each every tenth draw was retained). We considered the attribute *Percentage of friends already using the app* as numeric and the remaining attributes as categorical, for which we used effects coding. We calculated the parameter estimates as the sample mean of the distribution generated by the MCMC draws.

Model quality. To evaluate the model quality we performed the out of sample prediction validity test described in the PS Study. We found an average prediction accuracy of 77% which is substantially larger than that of a baseline model making choices at random (25%, as before).

Results. Overall, compared to the PS study, we find higher variation in average importance between the attributes. The highest ranked attribute is the percentage of friends already using the app, followed by accessibility, authentication, customisation level, and video calls (see Table S4). On average, messaging apps that are also web accessible, have two or multi-factor authentication and have a medium-high level of customisation are more appealing than mobile only apps with simple authentication and low customisation level (see Fig. S4). We observe that 299 out of the 300 respondents have a positive partworth utility of the social signal, with the remaining one having a negative utility. For the reasons outlined in describing the PS study, we did not consider further in the analysis the participant with a negative partworth utility of the social signal. We measure the individual-level threshold using the methodology described in the Methods section of main text, and calculate the average threshold per product. The results can be seen in Figure S5.

Variable	Average Importance	Standard Deviation
% of friends already using the app	0.56692052	0.17081138
Accessibility	0.13926414	0.12564616
Authentication	0.12267676	0.08752978
Customisation level	0.10365652	0.06592403
Video calls	0.06748205	0.05570236

TABLE S4. **Average variable importance in the App Adoption Study**. The variables are ranked by importance.

Linearity of the social signal. To test the plausibility of assuming a linear coefficient of the social signal, we re-estimate the model treating the *percentage of friends already using the app* as a categorical variable. Figure S6 shows a close to linear increase of the utility of the social signal as a function of the number of adopters (Pearson's $r(1498) = 0.92$, $p < 0.001$, 95% CI=$[0.92, 0.93]$), supporting the use of a linear coefficient.

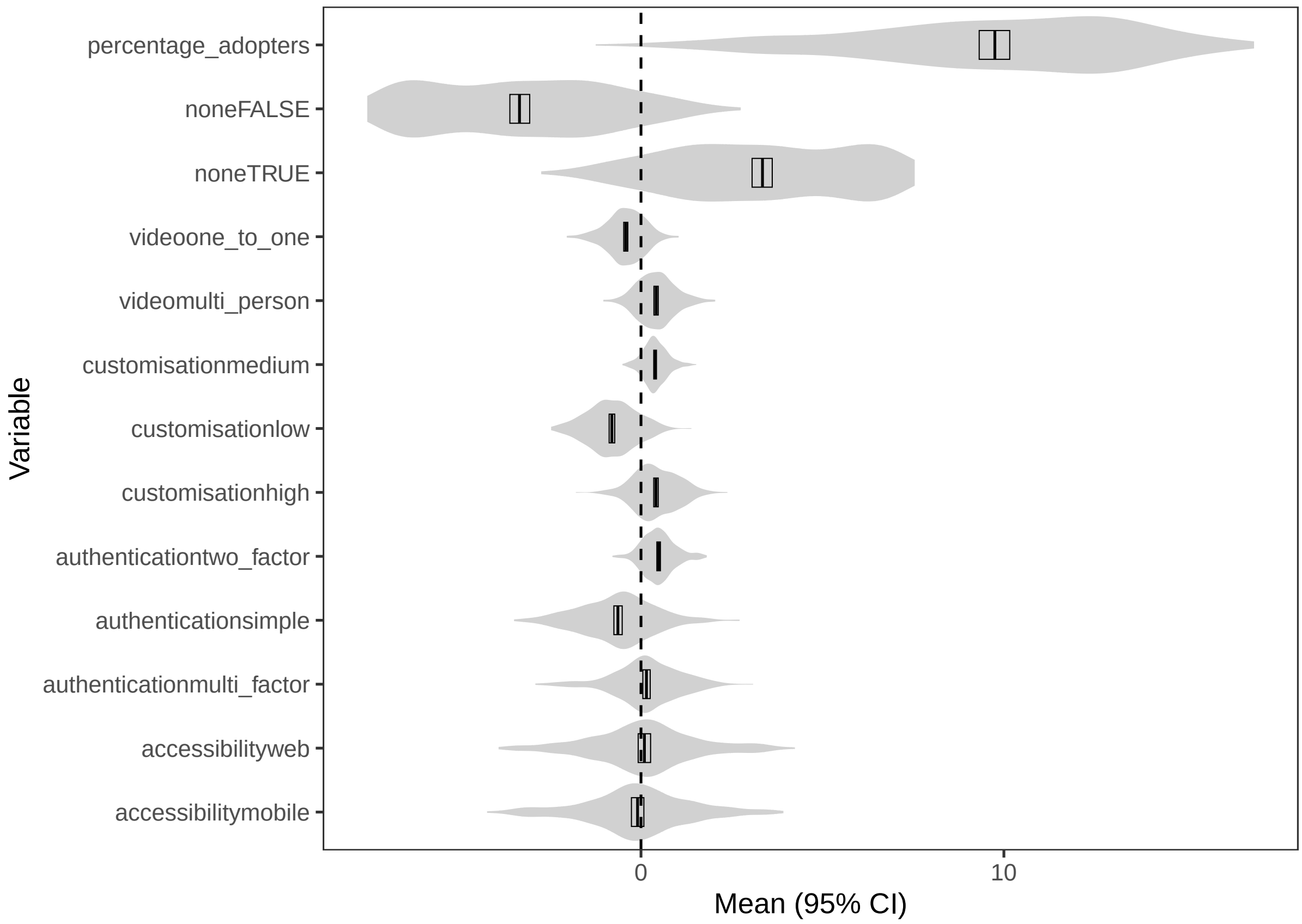

Fig. S4. **Average utilities across respondents in the AA Study.** The y axis contains all attributes and levels. The x axis shows the distribution of the marginal utility of each attribute level together with the mean and 95 % confidence interval (crossbars) based on $N = 300$ observations. On average, the percentage of adopters has a large, positive utility.

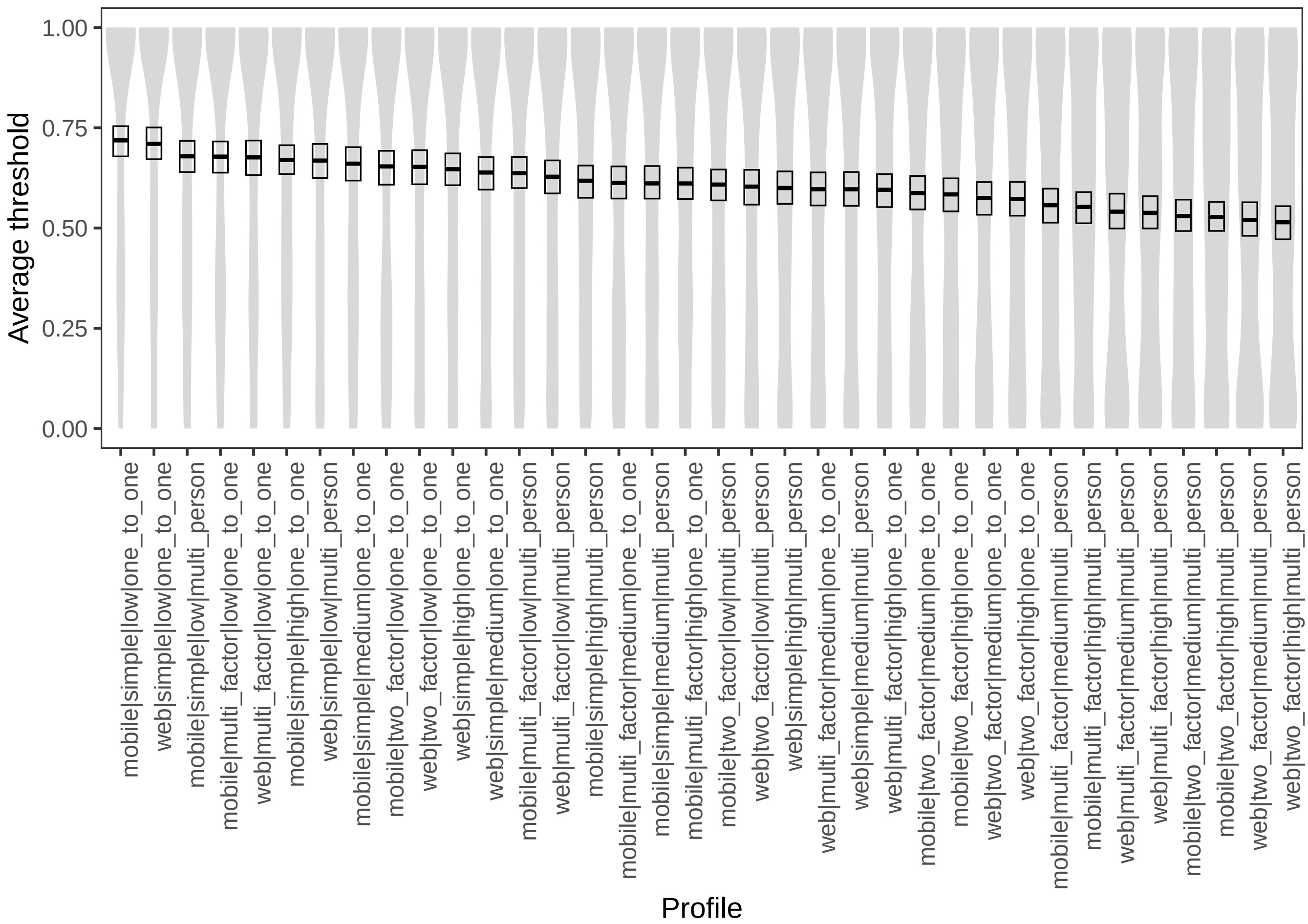

Fig. S5. **Threshold distribution across individuals (y axis) for each product (x axis) in the AA Study**. Crossbars represent the mean together with the 95% confidence interval based on $N = 299$ observations. The products are sorted by the mean threshold. We can observe some products have a lower mean threshold, implying they are more attractive. However, compared to the PS Study, the difference between the most and the least attractive product is considerably smaller.

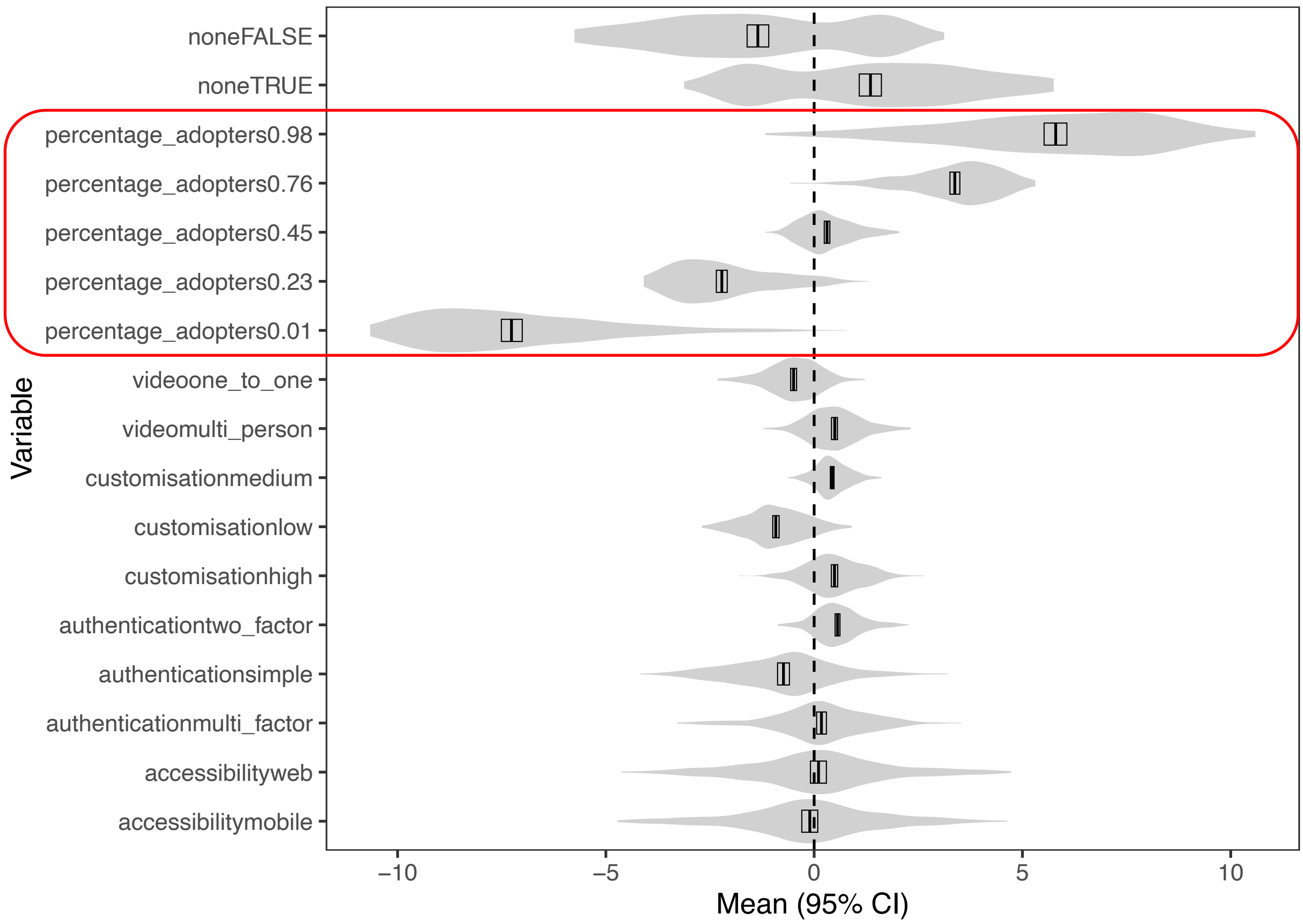

Fig. S6. **Average utilities across respondents treating the percentage of adopters as categorical variable (AA Study).** The y axis contains all the attributes and their levels. The x axis shows the distribution of the utility of an attribute level together with the mean and 95 % confidence interval (crossbars) based on $N = 300$ observations. On average, the utility of the percentage of adopters increases with the level (red rectangle).

S2. DATA FOR THE DATA-DRIVEN SIMULATIONS

A. Social network data

The original Add Health data [10] contains 85 networks. To save computational time, we restrict the analysis to a sample of 18 networks spanning diverse network properties. We constructed the sample as follows. For every network, we computed the number of nodes and the global transitivity index of the graph (i.e., the number of observed transitive triads divided by the number of potential transitive triads [11]). For each of the two metrics, we computed the 33% and 67% quantiles and labelled each network with respect to the metric as: low (metric < 33% quantile), medium (metric lies between the 33% and the 67% quantiles) or high (metric > 67% quantile). In this way, each network received two labels, one for the number of nodes and the other for transitivity. We created all possible combinations of the two labels (9 in total) and sampled two networks from each combination.

B. Product sampling

The total number of products that can be generated from a conjoint design is equal to all distinct combinations of attribute levels (not considering the social signal). Thus, there are 768 and 36 possible products in the PS and AA study, respectively. To save computational time, we decided to restrict our analysis to: (1) all 36 products in the AA study and (2) a subset of 36 products from the PS study. We selected the 36 products from the PS study such that the average threshold over respondents per product spans a broad range of observed values. Specifically, we calculated the threshold of every respondent for every product as described in the Methods section in the main text. Then we calculated the average threshold per product over respondents. We divided the range of the average threshold values in 6 equally-sized intervals (based on the 16.7%, 33%, 50%, 67% and 83% quantiles) and sampled 6 products from each interval.

S3. SIMULATED CONJOINT SURVEYS

The main objective of this Supplementary Note is to describe the procedure to generate simulated conjoint surveys (Section S3 A) and the obtained results, which concern both the accuracy and robustness of the threshold estimation procedure (Sections S3 B–S3 D). The simulated surveys are generated as described in Section S3 A are also used to inform the seeding simulations (see main text).

A. Survey simulation

We consider a scenario where a change practictioner needs to estimate individual thresholds to inform behavior-based seeding policies. As explained in the main text, we initially assume that the change practitioner has been able to survey the nodes of the network. For our simulation study, we simulate this survey. To simulate the survey, we follow a three step procedure, similar to the one described in Hein et al. [12]: (1) We calibrate the agents' partworth utilities on the empirical data from the two conjoint experiments (PS and AA); (2) We generate choice tasks; (3) We simulate the agents' choices in the choice tasks based on their calibrated partworth utilities. To ensure that our results do not depend on a specific realization of the data, for each of the two empirical conjoint studies (PS and AA), we generate 5 independent realizations of the choice simulation following the procedure detailed below.

1. Calibration of partworth utilities. Each agent is characterized by an n_{par}-dimensional vector whose elements constitute the partworth utilities. Thus $n_{par} = 24$ in the PS study and $n_{par} = 13$ in the AA study (one parameter for each level of each attribute, one parameter for the social signal, and two parameters for the none option). To calibrate the choice simulations with the empirical data from the conjoint experiments, we endow each agent with a ground-truth partworth utility vector equal to the estimated partworth utilities of a participant selected at random from the pool of real-world participants[3] without replacement. The resulting distributions of partworth utilities across agents are shown in Figs. S7−S8.

---

[3] Excluding the few participants with $\hat{\gamma} < 0$, see Section S1.

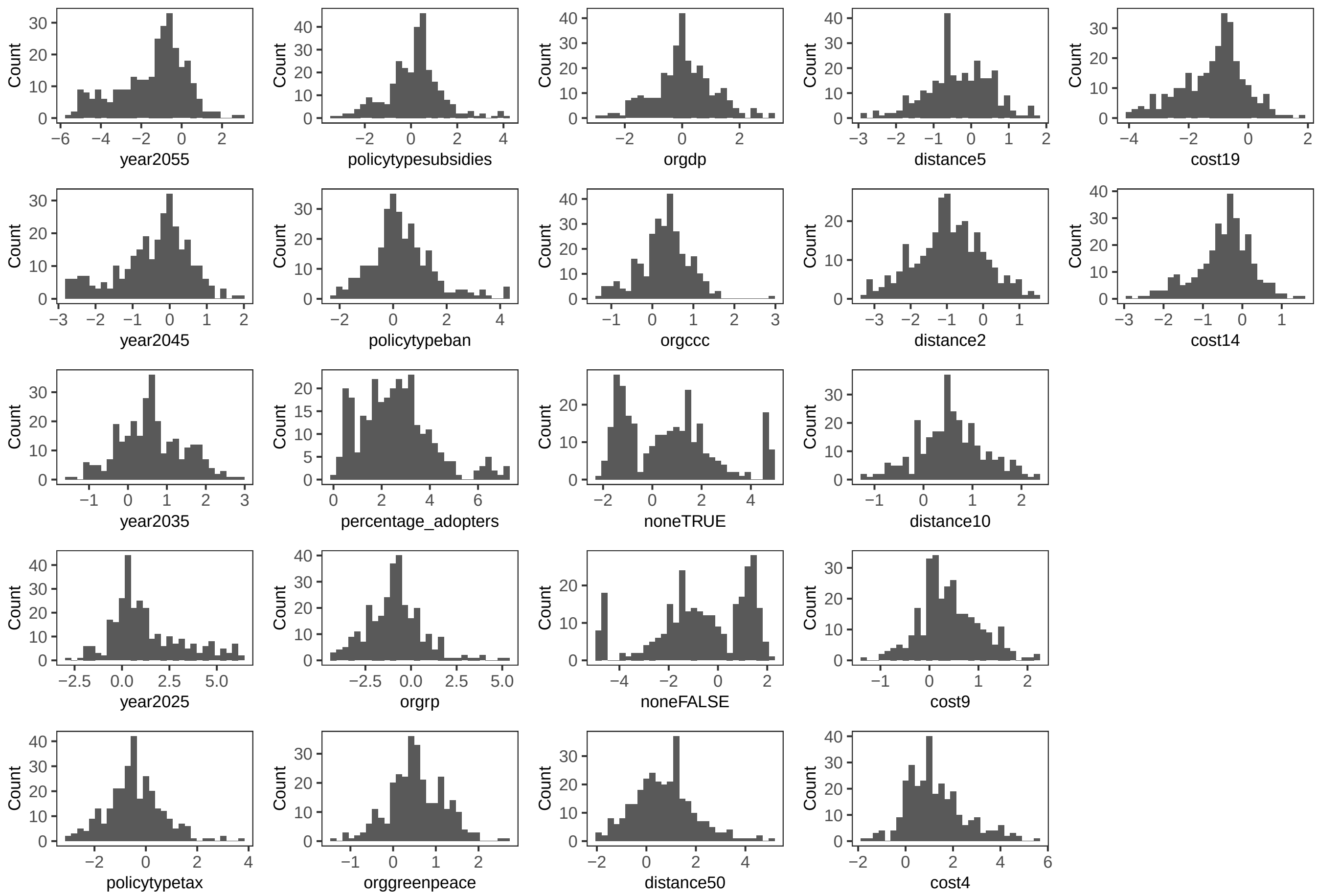

Fig. S7. **Distribution of ground-truth partworth utilities for the simulation calibrated with the results of the PS Study ($N = 291$ observations).**

2. Generation of choice tasks. We generate $Z = 15$ choice tasks per agent, each with a choice set consisting of two product alternatives and a none option. Each product alternative in the choice set is described as a unique combination of the product attributes from the focal conjoint study, and one out of three levels of the social signal: 0.1, 0.5 or 0.9. For every agent and every choice set, we randomly sample the two alternatives from the set of all possible product configurations.

3. Choice simulation. We simulate the conjoint survey by simulating the choices made by the agents. To simulate which alternative was selected in each choice set, we compute the utilities corresponding to each alternative, and the utility of the none option. For each agent, we compute the utility of a given alternative by summing up the attribute utilities for that alternative and the utility of the social signal corresponding to the given social signal level. We assume the utility of the none option is constant within each agent across choice tasks. We further assume there is some noise in the process[4], and add to the utility of each alternative (either product or none) a random component drawn from a Gumbel distribution with scale parameter $\theta = 1$ [12]. Lastly, we assume that the agent selects the alternative with the highest total utility.

B. Parameter recovery

For each simulated choice dataset, we recover the individual partworth utilities using the Hierarchical Bayes algorithm (similarly to what we did in the two real conjoint studies, see Supplementary Note S1). We used 30'000 MCMC

[4] In this context, the presence of noise can reflect scenarios where the attributes included in the conjoint are not sufficient to fully capture the total utility of a product.

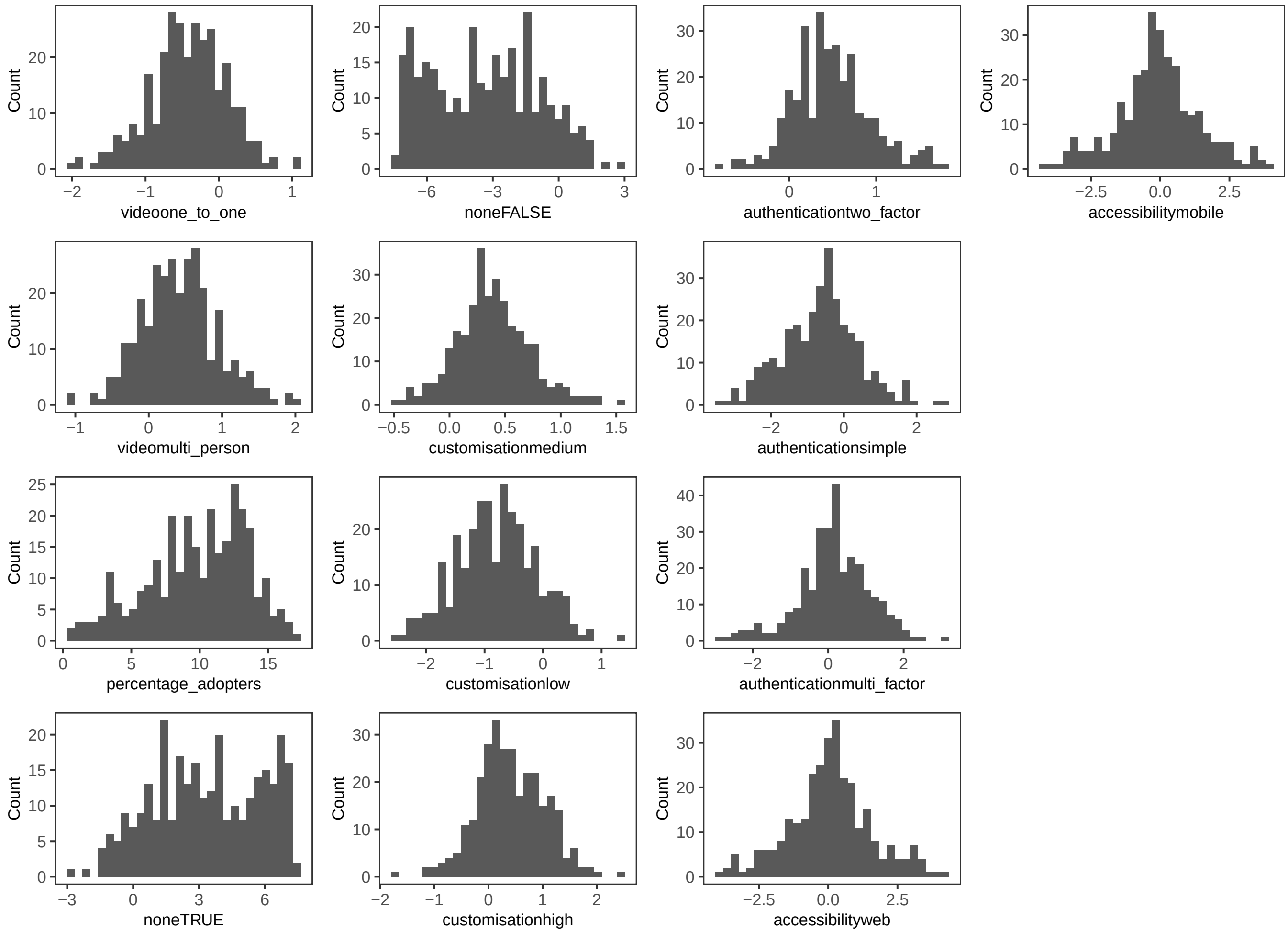

Fig. S8. **Distribution of ground-truth partworth utilities for the simulation calibrated with the results of the AA Study ($N = 299$ observations).**

iterations, the first 10'000 for burn in and the remaining 20'000 for parameter estimation, from which every tenth draw was retained. We considered the social signal attribute as numeric and the remaining attributes as categorical, for which we used effects coding. We calculated the parameter estimates as the sample mean of the distribution generated by the MCMC draws. We used the estimated parameters to calculate the estimated thresholds through Eq. (1) in the main text.

We tested the quality of the parameter recovery in two ways, similarly to Hein et al. [12]. First, we calculated the mean Pearson correlation between the ground-truth and the estimated partworth utilities over all parameters. As the average of Pearson correlations does not converge to the true correlation, following Hein et al. [12], we first rescaled the correlation coefficients using Fisher's z-transformation $[f(x) = 0.5 \log((1 + x)/(1 - x))]$, averaged the rescaled correlations, and converted the resulting average back to the original scale using the inverse of Fisher's z-transform. We found the mean correlation ranges over simulated datasets from 0.74 to 0.76 in the PS study and from 0.72 to 0.74 in the AA study.

Second, we measured how often the true coefficients lie within the 95% credible intervals of the estimated coefficients. We found the average value over coefficients and agents ranges across simulated datasets from 95.02% to 96.34% in the PS study and from 94.78% to 96.32% in the AA study. As the average correlation coefficients are large and the true coefficients lie almost always within the 95% credible intervals of the estimated coefficients, we conclude the parameter recovery is accurate.

C. Sensitivity to prior specification

We also use the simulated conjoint to test the robustness of individual-level estimates with respect to the specification of the prior distributions.

Motivation. Because of the hierarchical structure, each individual's partworth utilities are influenced not only by their own choices but also by information from the rest of the sample. Specifically, each individual's vector of partworth utilities is assumed to come from a population-level distribution (first-stage prior) whose parameters are themselves estimated from the data conditional on assumptions about their distribution (second-stage priors). As a result, each posterior estimate reflects a combination of the respondent's own data and the group-level information. This effect shrinks individual estimates toward the estimated group mean. The extent of shrinkage depends both on the amount of data available for each individual and on the prior distributions specified for the group-level parameters. Formally, following the notation in main text, the utility of individual n for alternative i is given by $U_{ni} = \sum_{k=1}^{K} \beta_{nk}\, x_{ki} + \gamma_n s_{ni} + \epsilon_{ni}$. Let β'_n denote the vector of all parameters (β_n and γ_n) for individual n. Under the frequently-used normal prior model, the vector of parameters follows a multivariate normal distribution $\beta'_n \sim \mathcal{N}(b, \Sigma)$ (first-stage prior) with $b \sim \mathcal{N}(\bar{b}, A)$ as (second-stage) prior on the population mean and $\Sigma \sim \Psi(\nu, V)$ as (second-stage) prior on the population covariance, where $\Psi(\nu, V)$ denotes the inverted Wishart (IW) distribution with ν degrees of freedom and scale matrix V [13]. It is typical to reflect the researcher's uncertainty by controlling the prior on the covariance matrix and assume a very diffuse prior on the population mean (e.g., mean of b at 0 and very large variance) [3]. Tighter priors on Σ result in a stronger shrinkage toward the group mean, whereas looser priors allow for more individual-specific variation [14]. As the amount of data at the individual level increases, the effect of the prior specification diminishes [13].

A potential concern is that different specifications of the priors on Σ (different degrees of freedom and/or scale matrices in $\Psi(\nu, V)$) might lead to different estimated partworth utilities and consequently to different adoption thresholds. The effect of prior specification of the covariance matrix on the accuracy of recovering individual-level parameters has been addressed in an extensive simulation study by Ref. [14]. Covering a wide range of conditions, the study finds that while the performance is influenced by the specifications of the priors, it generally remains high – except in specific cases, such as when there is a substantial mismatch between the prior variance and the true heterogeneity in the data [14]. Using a similar but simplified setup, our objective here is to determine whether the main results in main text are robust with respect to the tightness of the prior and consequently to the degree of shrinkage it induces.

Method. To address this concern, we repeat the simulation setup, generating datasets with varying numbers of choice tasks per individual $Z \in [15, 30, 100]$ consisting of the original dataset ($Z = 15$ tasks) and two additional simulated datasets (corresponding to $Z = 30$ and $Z = 100$ tasks, respectively). From each dataset, we estimate the partworth utilities using HB with four prior specifications. Following a standard approach, for all HB models, we use a diffuse prior on the population mean [3, 13], as implemented in the default option in the software package: $\bar{b} = \vec{0}$ (vector of zeroes) and $A = 100 \cdot \Sigma$ [8]. We vary the prior on the covariance matrix Σ as follows. Following the notation in the documentation of the software package [8], $\nu = df + K'$ and $V = V_0 \cdot \nu \cdot v$, where K' is the number of parameters to be estimated per individual (16 in the PS Study and 8 in the AA Study), V_0 is an $K' \times K'$ block diagonal matrix (see [8] for its specification), $v > 0$ controls the prior variance and $df \geq 2$ controls the degrees of freedom of the IW distribution [8]. Therefore, in the current software implementation, to control the tightness of the prior on the covariance matrix, the researcher can adjust the degrees of freedom (through df) and the prior variance (through v and indirectly through df). Lower values of df and higher values of v lead to looser priors, reducing the influence of the group average [14]. We consider four specifications: default ($df = 5$, $v = 2$), tight ($df = 10$, $v = 2$), loose ($df = 2$, $v = 2$) and uninformative ($df = 2$, $v = 100$). For illustration, in Table S5 we show the mean of the IW distribution with the above specifications, from which the covariance matrices are drawn in the AA Study. The tightness of the prior can be understood by comparing the variance (diagonal) entries to the range of the partworth coefficients (see Fig. S4 for the AA Study). Standard deviations (i.e., the square roots of variances in the diagonal entries) that are large relative to the typical magnitude of the partworths correspond to loose priors, while smaller standard deviations correspond to tight priors.

For the MCMC estimation we use $30'000$ iterations ($10'000$ for burn in and $20'000$ for estimation) for all except the uninformative prior, where we use $50'000$ ($30'000$ for burn in and $20'000$ for estimation) due to slower convergence. In all cases, we retained every 10th draw for parameter estimation. We use three measures to calculate the estimation accuracy:

- Mean absolute error (MAE) in recovering the ground-truth partworth utilities, defined as the average absolute value of the difference between the true and the estimated partworth utilities: MAE $= 1/(HNK') \sum_{h=1}^{H} \sum_{n=1}^{N} \sum_{k=1}^{K'} |\hat{\beta}'_{hnk} - \beta'_{hnk}|$, where $H = 5$ is the number of realizations of the simulated choice data, N is the number of simulated individuals, K' is the number of parameters (attribute utilities and utility

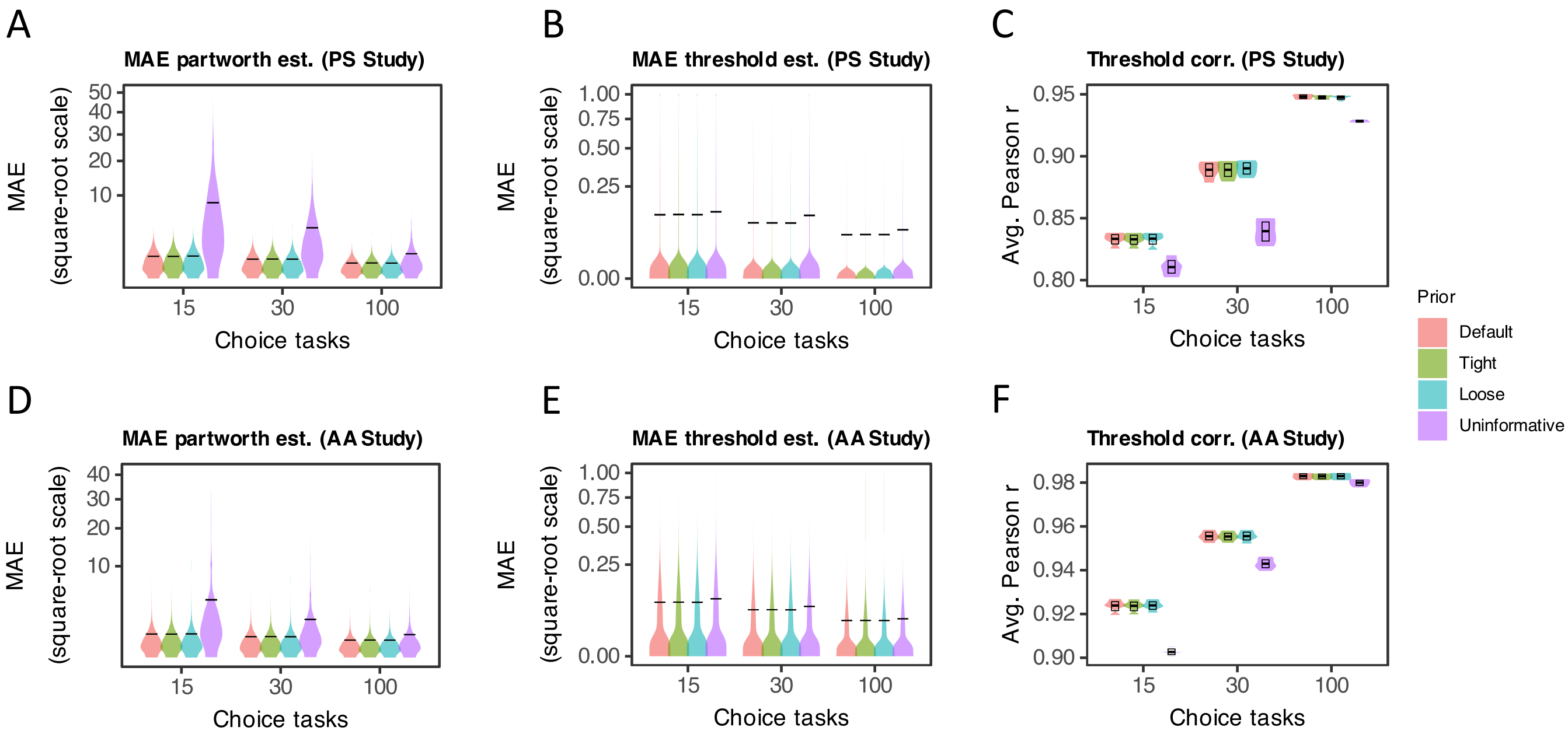

Fig. S9. **Estimation accuracy under different prior specification for the PS Study (top row) and the AA Study (bottom row).** All but the uninformative prior result in similar accuracy of recovering the partworth utilities **(A, D)** and adoption thresholds **(B, E)** and similar correlations between the true and the estimated thresholds **(C, F)**. Crossbars represent the mean together with the 95% confidence intervals based on the following number of observations: 32,010 (A), 46,548 − 52,236 (B); 19,435 (D); 53,424 − 53,712 (E); 5 (C, F).

of the social signal), β'_{hnk} is the true value in simulated choice data h of parameter k for simulated individual n, $\hat{\beta}'_{hnk}$ is the corresponding estimated value.

- Mean absolute error of recovering the individual thresholds. Similarly as above, it is defined as the average absolute value of the difference between the true and the estimated thresholds over all simulated individuals, products and realizations of the simulated choice data[5].
- Average Pearson's correlation between the true and the estimated thresholds. The correlation is computed based on data pooled from all individuals and all products. The correlations are then rescaled using Fisher's z-transformation, averaged over the five realizations of the simulated choice data and scaled back using the inverse Fisher's z-transformation, as described in Supplementary Note S3 B.

Results. We find that all but the uninformative prior produce similarly accurate partworth estimation (consistent with [14]), and all methods' mean absolute error decreases as the data size increases (Figs. S9A,D). The differences between the models' accuracy is even smaller for the threshold estimation task (Figs. S9B,E). Importantly, the thresholds estimated through the different models are highly correlated with the ground-truth thresholds (Figs. S9C,F). For all priors and number of choice tasks, the average Pearson correlations are larger than 0.81 and 0.90 in the PS and AA Study, respectively. Taken together, these findings indicate that as long as the choice of priors is sensible, the estimated thresholds are robust with respect to prior specification.

D. Understanding the effect of partworth modality on estimation accuracy

Motivation. To derive the results of the sensitivity analysis in Section S3 C, we calibrated the choice simulations by setting the ground-truth partworth utilities equal to the individual-level estimates from the two conjoint studies.

[5] For all priors, the estimated partworth utility of the social signal is negative for a small subset of simulated individuals. As previously noted, in these situations, thresholds are not defined under the adopted choice framework. These cases are therefore removed from the analysis. As the number of removals differs slightly across priors, the sample size used for computing the threshold-estimation MAE also differs. This leads to the small variation in observation counts reported in Figs. S9B,E and S11B,E.

TABLE S5. **Mean of IW distributions under different priors in the AA study.** Larger variances (diagonal entries) correspond to looser priors. The reason why there are less variables compared to Fig. S4 is that for categorical variables the utility of the reference level is uniquely determined by the estimated utilities of the other levels.

	acc. mobile	auth. simple	auth. two_factor	cust. low	cust. medium	video multi_person	% adopters	none
acc. mobile	2.00	0.00	0.00	0.00	0.00	0.00	0.00	0.00
auth. simple	0.00	2.67	-1.33	0.00	0.00	0.00	0.00	0.00
auth. two_factor	0.00	-1.33	2.67	0.00	0.00	0.00	0.00	0.00
cust. low	0.00	0.00	0.00	2.67	-1.33	0.00	0.00	0.00
cust. medium	0.00	0.00	0.00	-1.33	2.67	0.00	0.00	0.00
video multi_person	0.00	0.00	0.00	0.00	0.00	2.00	0.00	0.00
% adopters	0.00	0.00	0.00	0.00	0.00	0.00	4.00	0.00
none	0.00	0.00	0.00	0.00	0.00	0.00	0.00	2.00

(a) Tight prior (v=2, df=10)

	acc. mobile	auth. simple	auth. two_factor	cust. low	cust. medium	video multi_person	% adopters	none
acc. mobile	3.25	0.00	0.00	0.00	0.00	0.00	0.00	0.00
auth. simple	0.00	4.33	-2.17	0.00	0.00	0.00	0.00	0.00
auth. two_factor	0.00	-2.17	4.33	0.00	0.00	0.00	0.00	0.00
cust. low	0.00	0.00	0.00	4.33	-2.17	0.00	0.00	0.00
cust. medium	0.00	0.00	0.00	-2.17	4.33	0.00	0.00	0.00
video multi_person	0.00	0.00	0.00	0.00	0.00	3.25	0.00	0.00
% adopters	0.00	0.00	0.00	0.00	0.00	0.00	6.50	0.00
none	0.00	0.00	0.00	0.00	0.00	0.00	0.00	3.25

(b) Default prior (v=2, df=5)

	acc. mobile	auth. simple	auth. two_factor	cust. low	cust. medium	video multi_person	% adopters	none
acc. mobile	10.00	0.00	0.00	0.00	0.00	0.00	0.00	0.00
auth. simple	0.00	13.33	-6.67	0.00	0.00	0.00	0.00	0.00
auth. two_factor	0.00	-6.67	13.33	0.00	0.00	0.00	0.00	0.00
cust. low	0.00	0.00	0.00	13.33	-6.67	0.00	0.00	0.00
cust. medium	0.00	0.00	0.00	-6.67	13.33	0.00	0.00	0.00
video multi_person	0.00	0.00	0.00	0.00	0.00	10.00	0.00	0.00
% adopters	0.00	0.00	0.00	0.00	0.00	0.00	20.00	0.00
none	0.00	0.00	0.00	0.00	0.00	0.00	0.00	10.00

(c) Loose prior (v=2, df=2)

	acc. mobile	auth. simple	auth. two_factor	cust. low	cust. medium	video multi_person	% adopters	none
acc. mobile	500.00	0.00	0.00	0.00	0.00	0.00	0.00	0.00
auth. simple	0.00	666.67	-333.33	0.00	0.00	0.00	0.00	0.00
auth. two_factor	0.00	-333.33	666.67	0.00	0.00	0.00	0.00	0.00
cust. low	0.00	0.00	0.00	666.67	-333.33	0.00	0.00	0.00
cust. medium	0.00	0.00	0.00	-333.33	666.67	0.00	0.00	0.00
video multi_person	0.00	0.00	0.00	0.00	0.00	500.00	0.00	0.00
% adopters	0.00	0.00	0.00	0.00	0.00	0.00	1000.00	0.00
none	0.00	0.00	0.00	0.00	0.00	0.00	0.00	500.00

(d) Uninormative prior (v=100, df=2)

From the so-calibrated ground-truth utilities, we generated the simulated choice datasets, which we subsequently used to test the recovery of the partworth utilities via HB estimation under different specifications of the normal prior. One concern is that in those simulations, the input parameters (see Figs. S7−S8 for their distribution) were themselves estimated through the same HB method whose sensitivity we evaluate. As a result, the analysis may not reflect the method's sensitivity in scenarios where the underlying preference distribution is more complex or irregular. To address this concern, we extend the sensitivity analysis to settings in which the underlying distribution of ground-truth partworth utilities is not entirely derived from the estimation method. Specifically, we consider the more complex case of multimodal distributions, which would occur in scenarios where the population consists of multiple latent segments

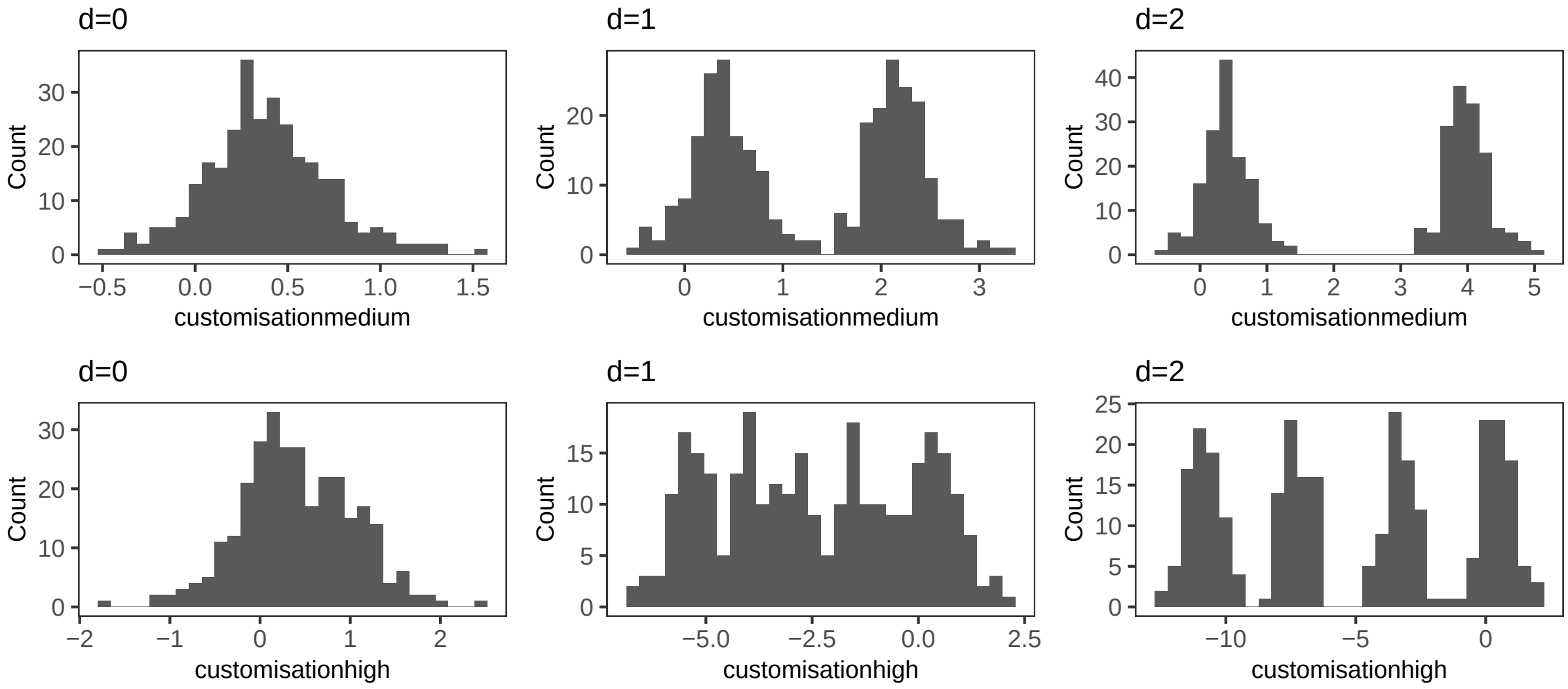

Fig. S10. **Distribution of two ground-truth partworth utilities for different values of the shifting factor ($d \in \{0, 1, 2\}$).** The top row shows the distribution of the partworth utility for the *medium* level of the *customisation* attribute in the AA study. Here $d = 0$ corresponds to the calibrated distribution used as ground-truth in Section S3 C. As d increases (from right to left), the distributions becomes increasingly more bimodal; when $d = 2$, the two modes are more widely separated. The bottom row shows the distribution of the reference level (*high*) of the same attribute. Because reference-level utilities under effect coding are computed from the other levels, the resulting distribution can have more than two modes (here four modes, that are clearly separated for $d = 2$). Each panel is based on $N = 299$ observations.

with distinct preferences.

Method. We repeat the analysis in Section S3 C, considering 15 choice tasks per agent. In Section S3 C, the ground-truth partworth utilities corresponded to the individual-level HB estimates. We further refer to them as the *calibrated distributions*. Here, we generate multimodal ground-truth distributions by perturbing the calibrated distributions as follows. For each partworth utility, we randomly select 50% of the agents and shift their partworth utilities by an amount equal to the range of the calibrated distribution (maximum minus minimum) multiplied by a shift factor $d \in \{1, 2\}$. Fig. S10 illustrates the resulting distributions of selected ground-truth partworth utilities for different values of d. Based on the so-obtained ground-truth partworth utility distributions, we conduct five independent choice simulations for each d value, using the same procedure described in Section S3 C. For the HB estimation of the generated choice data, we use the same prior specifications and the same evaluation metrics described in Section S3 C. When evaluating the accuracy of recovering the ground-truth partworth utilities, we normalize the MAE by $d + 1$ to account for the differences in coefficient scales across scenarios.

Results. The results for the informative priors are in qualitative agreement with those in Section S3 C, which are shown for reference in the charts for $d = 0$. All priors except the uninformative one produce similarly accurate estimates of the partworth utilities (Figs. S11A,D), while all priors produce similarly accurate estimates of the adoption thresholds (Figs. S11B,E) and comparable correlations between the true and estimated thresholds (Figs. S11C,F).

In the multimodal scenarios ($d \in \{1, 2\}$), the uninformative prior outperforms the rest in partworth estimation. This arguably occurs because, when the true distribution of partworths is multimodal, a tighter normal prior induces stronger shrinkage of individual estimates toward the global mean, thereby masking the underlying multimodality and increasing bias [13]. Similarly, the disadvantage of the uninformative prior for threshold estimation observed in the calibrated scenarios (Figs. S11B–C; E–F; bar charts at $d = 0$) vanishes in the multimodal scenarios ($d \in \{1, 2\}$).

Overall, these results show that under more challenging, multimodal preference structures, informative normal priors do not necessarily outperform an uninformative prior. As expected, the correlation between estimated and ground-truth thresholds decreases relative to the calibrated simulations[6] (Figs. S11C,F). Nevertheless, the correlations remain high across all scenarios [larger than $r = 0.74$ ($r = 0.79$) in the PS (AA) Study], pointing to a reasonable robustness of

[6] The findings for the MAE of the threshold estimation (Figs. S11B,E) seem in apparent contradiction with this result, as they show a decreasing MAE as the shifting factor increases. This apparent contradiction can be resolved by noting that for larger d, the threshold distribution becomes more degenerate (i.e., it consists of more 0s and 1s). In such cases, minimizing the MAE is inherently simpler than in the original case ($d = 0$), as a naive model that always "guesses" the dominant value can achieve artificially high accuracy. To illustrate this point, we

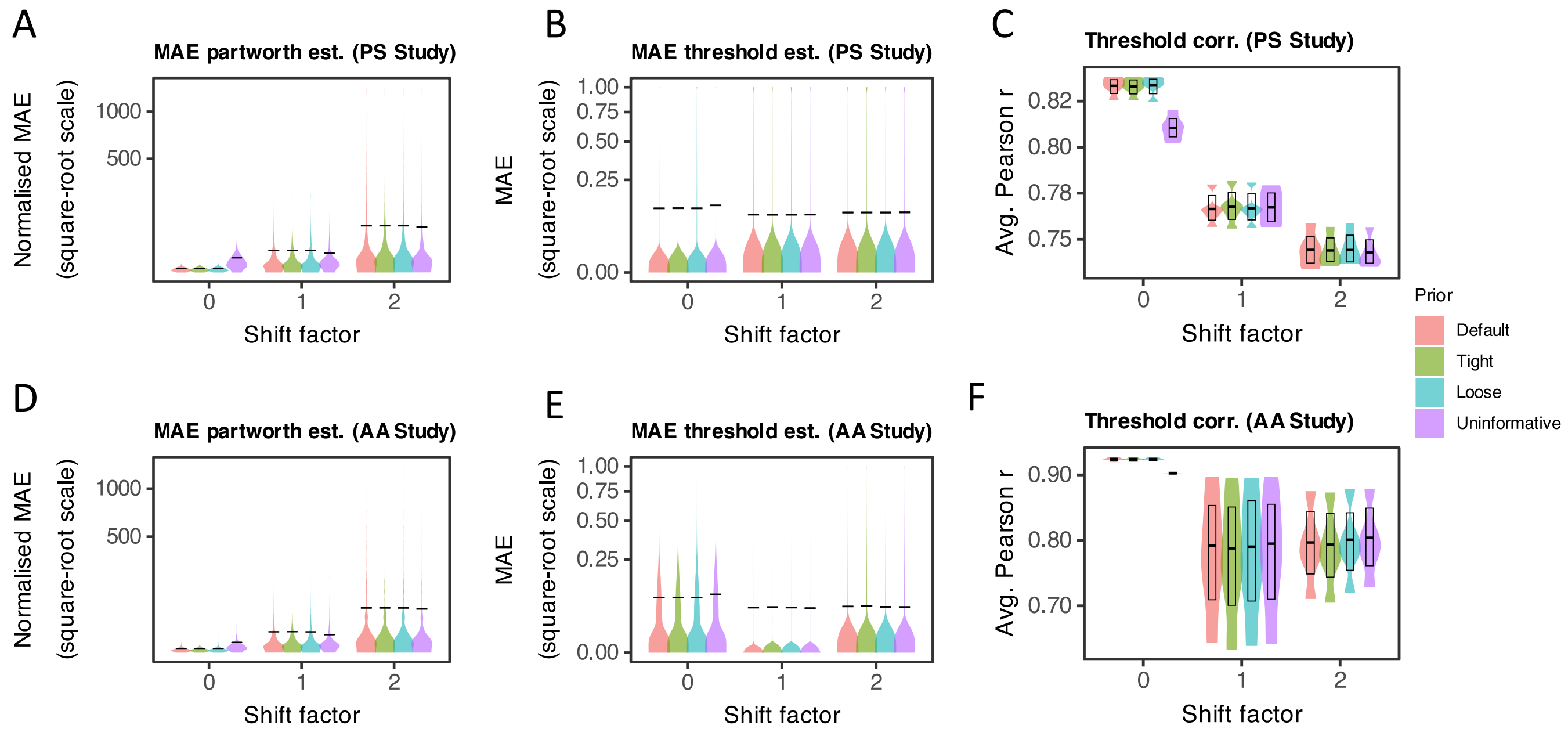

Fig. S11. **Estimation accuracy under different prior specification for the PS Study (top row) and the AA Study (bottom row)**. The x-axis represents the value of the shift factor d used to generate the ground-truth partworth distribution. All but the uninformative prior result in similar accuracy of recovering the partworth utilities **(A, D)** and adoption thresholds **(B, E)** and similar correlations between the true and the estimated thresholds **(C, F)**. represent the mean together with the 95% confidence intervals based on the following number of observations: $32,010$ (A), $50,508 - 52,344$ (B); $19,435$ (D); $52,992 - 53,748$ (E); 5(C, F).

the estimated thresholds against different preference scenarios. We note that in scenarios where underlying multimodal distributions are to be expected, extensions of the HB model (such as those using mixtures of normals [13]) might improve estimation accuracy.

---

additionally evaluate the performance of a naive baseline model where we estimate the HB model, predict the thresholds of all the individuals for a given product, and then assign to all the individuals the most frequently predicted value among 0 or 1. The performance of this baseline model improves substantially as the shift factor increases: by 42%(30%) for $d = 1$ compared to $d = 0$, and by 44%(32%) for $d = 2$ compared to $d = 0$ in the PS (AA) Study. Hence, while the MAE decreases as d increases, the MAE reduction compared to the baseline's MAE actually shrinks, in qualitative agreement with Figs. S11C,F.

S4. SEEDING POLICIES

A. Structure-based seeding policies

Degree policy. The degree assumes that a node is central if she has many connections. A node's degree is simply defined as its number of connections [15]. Despite the simplicity of this metric, some works have found high-degree nodes to be effective at predicting and triggering large-scale cascades of adoptions [16–18], although some works found counterexamples in model-based and empirical data, e.g., [19–22]. We compute it via the `igraph` package [23].

Betweenness policy. The betweenness centrality assumes that a node is central if she is traversed by many of the network's shortest paths. For a focal node i, it averages over all pairs of nodes (s, t) the fraction of shortest paths between s and t that pass through i [15]. Similarly to high-degree nodes, some works have found high-betweenness nodes to be effective at triggering large-scale cascades of adoptions [17], although some works found counterexamples in model-based and empirical data, e.g., [24, 25]. We compute it via the `igraph` package [23].

Closeness policy. The closeness centrality assumes that a node is central if it is close to many other nodes in the network. It is defined as the inverse of the sum of shortest-path distances from the focal node to all the other nodes in the network [26]. Some works have found high-closeness nodes to be effective at triggering large-scale cascades of adoptions, especially for simple contagions above their critical point [27]. We compute it via the `igraph` package [23].

Collective Influence policy. The collective influence metric is derived analytically as an approximate solution to a network dismantling problem. In this problem, one seeks to find the minimal set of nodes whose removal from the network causes the collapse of the network's largest component [28]. We compute it via our own function.

B. Behavior-based seeding policies

For each configuration $\mathcal{C}$ (identified by the network being analyzed, the product that spreads through the network, the agents' random assignment to the network's nodes, and the particular realization of the simulated conjoint survey performed by the change practitioner to estimate the thresholds), we measure various node-level behavior-based metrics that take into account the estimated thresholds (see main text and Supplementary Note S3). The underlying assumption is that the social change practitioner has been able to survey the nodes of the network, estimate their thresholds for different products, and use the estimated thresholds to implement the seeding policies defined below.

Low threshold policy. The low-threshold policy simply selects as the focal seed the node with the lowest estimated threshold in a given configuration; In case of ties, the focal node is selected at random among those with the lowest estimated threshold.

Neighborhood susceptibility policy. The neighborhood susceptibility (NS) of a given node i is defined as her number of low-threshold (highly-susceptible) social contacts. Following Watts [29], we define a given node j is a low-threshold node if $\hat{\tau}_j \leq d_j^{-1}$, where $\hat{\tau}_j$ is the threshold estimated by the social change practitioner and d_j is the degree of node j; if this is the case, a single adoption event in j's neighborhood is sufficient to trigger their adoption. Therefore, high-NS nodes are not necessarily the most central ones in the network according to the degree, but they are surrounded by a local "mass" of highly-susceptible individuals. Differently than the degree and other standard centrality metrics, the NS metrics takes into account individual-level behavioral information, namely, individuals' thresholds.

Calibrated and uncalibrated complex centrality (CC) policies. The complex centrality metric has been introduced by Guilbeault and Centola [10] to identify the optimal network locations where to initiate a complex contagion process under a clustered seeding strategy. In Guilbeault and Centola [10]'s code, the complex centrality metric is measured by running the simulation of the complex contagion process under the ground-truth thresholds, and then measuring the average length of the shortest paths that connect a given node to the reachable nodes in the "activated network" composed of the nodes who adopted. We adapt their code to our study by measuring the complex centrality based not on the ground-truth thresholds (which are typically unknown to the decision maker), but on different assumptions on which individual-level behavioral data is available to the social change practitioner. For simplicity, we consider here two scenarios.

- *Calibrated complex centrality (CCC).* We assume that the change social practitioner has been able to survey the network's nodes, and uses the estimated thresholds to run the simulations behind the complex centrality calculation.

- *Uncalibrated complex centrality (UCC).* We assume that the change practitioner has no behavioral information about the network's nodes. Following the same procedure used by Guilbeault and Centola [10] in their analysis of Indian villages, we average each node's complex centrality over an ensemble composed of five homogeneous absolute threshold scenarios ($\tau_i\, d_i = \theta$ where $\theta \in \{2, 3, 4, 5, 6\}$). As the uncalibrated CC assumes no knowledge

of the estimated thresholds, and it averages the CC over a range of arbitrarily-selected threshold values, we classify it as a structure-based policy in the main text.

We implemented our own function to generate synthetic choice data and estimate the thresholds from the simulated data (see Supplementary Note S3); we implemented the two types of CC-based policies by simply passing the corresponding threshold vectors into a function adapted from the code by Guilbeault and Centola [10].

S5. ADDITIONAL RESULTS FOR SEEDING POLICIES

A. Results for the ratio-to-best metric

We show here the results for the ratio-to-best metric. Specifically, to assess the relative performance of the examined seeding policies, for each analyzed configuration $\mathcal{C}$, we measure the simulated performance P of the nine policies. For each configuration $\mathcal{C}$, each method m is assigned to a performance variable that quantifies the method's performance compared to the best-performing method [27], $s_m(\mathcal{C}) = P_m(\mathcal{C})/\max_{m'}\{P_{m'}(\mathcal{C})\}$. We average this variable across all configurations. The results, shown in Fig. S12, are largely consistent with those shown in Fig. 2 of main text.

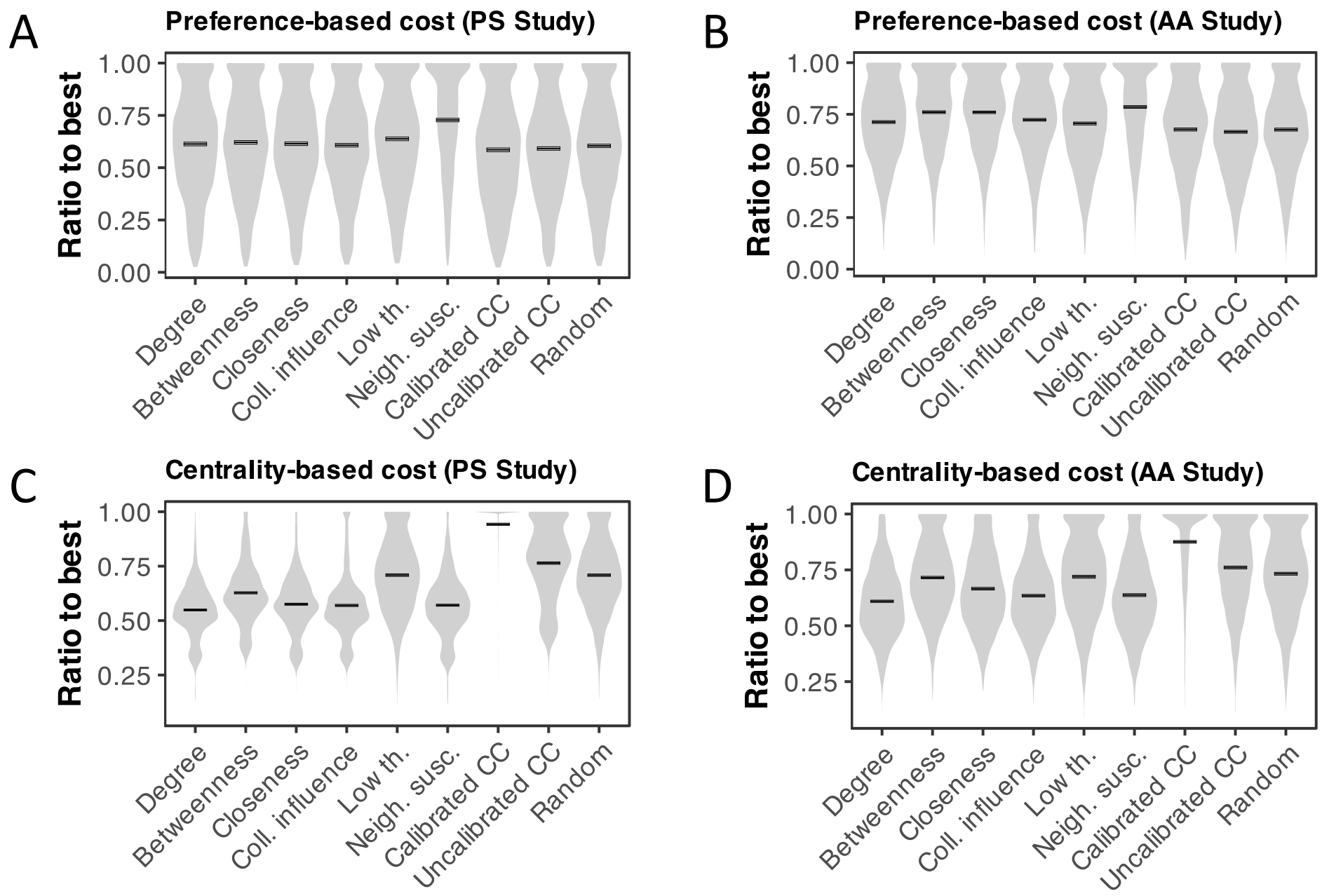

Fig. S12. **Relative performance of seeding policies according to the ratio-to-best metric. (A, B)** Relative performance of seeding policies under a preference–based cost structure, measured through the ratio-to-best metric defined above, for the policy support experiment and the app adoption experiment, respectively. The neighborhood susceptibility policy based on the estimated thresholds significantly outperforms the other policies (one-tailed Wilcoxon signed-rank test, all p-values are smaller than $p = 0.001$, the smallest effect size is $r = 0.09$ in the AA Study in the comparison against the betweenness centrality). **(C, D)** Relative performance of seeding policies under a centrality–based cost structure for the policy support experiment and the app adoption experiment, respectively. The complex centrality policy based on the estimated thresholds significantly outperforms the other policies (one-tailed Wilcoxon signed-rank test, all p-values are smaller than $p = 0.001$, the smallest effect size is $r = 0.42$ in the AA Study in the comparison against the uncalibrated complex centrality). Overall the results are in qualitative agreement with those obtained for the mean rank metric (Fig. 2 in the main text). In all the panels, crossbars represent the mean together with the 95% confidence intervals over $3,240$ runs.

B. Results for different seed set sizes

We show here the results for different seed set sizes. Compared to the seed set size used in main text ($z = 0.025$), we consider here both a smaller seed set size ($z = 0.0125$) and a larger one ($z = 0.05$). The results for the smaller seed set size are in qualitative agreement with those in the main text (see Figs. S13–S14). The only exception is the lack of significant differences in the performance of the uncalibrated complex centrality and random seeding in all setups except the preference-based cost in the PS Study (one-tailed Wilcoxon signed-rank test, all p-values are larger than $p = 0.23$; the largest effect size is $r = 0.04$ for the PS Study, ratio-to-best metric, centrality-based cost), which supports the conclusion that the complex centrality can only realize its full potential when calibrated with the estimated thresholds.

The results for the larger seed set size are in qualitative agreement with those in the main text (see Figs. S15–S16), except for the relative performance of the methods in the preference-based cost scenario for the AA Study. In this scenario, we find that while the neighborhood susceptibility still outperforms the degree and collective influence (one-tailed Wilcoxon signed-rank test, all p-values smaller than $p = 0.001$; the smallest effect size is $r = 0.21$ in the comparison against the collective influence for the average rank metric), it no longer significantly outperforms the betweenness and closeness centralities (one-tailed Wilcoxon signed-rank test, all p-values are larger than $p = 0.12$; the largest effect size is $r = 0.05$ in the comparison against the betweeness centrality for the average rank metric). The reason is arguably that with only one focal seed node selected by the neighborhood susceptibility metric, there is no guarantee that the additional seeds around the focal node require low cost for being successfully targeted. This limitation might be attenuated by network homophily, which we will investigate in a follow-up article. On the other hand, the calibrated complex centrality benefits from maximizing spreading size by taking into account clustered seeding in its calculation.

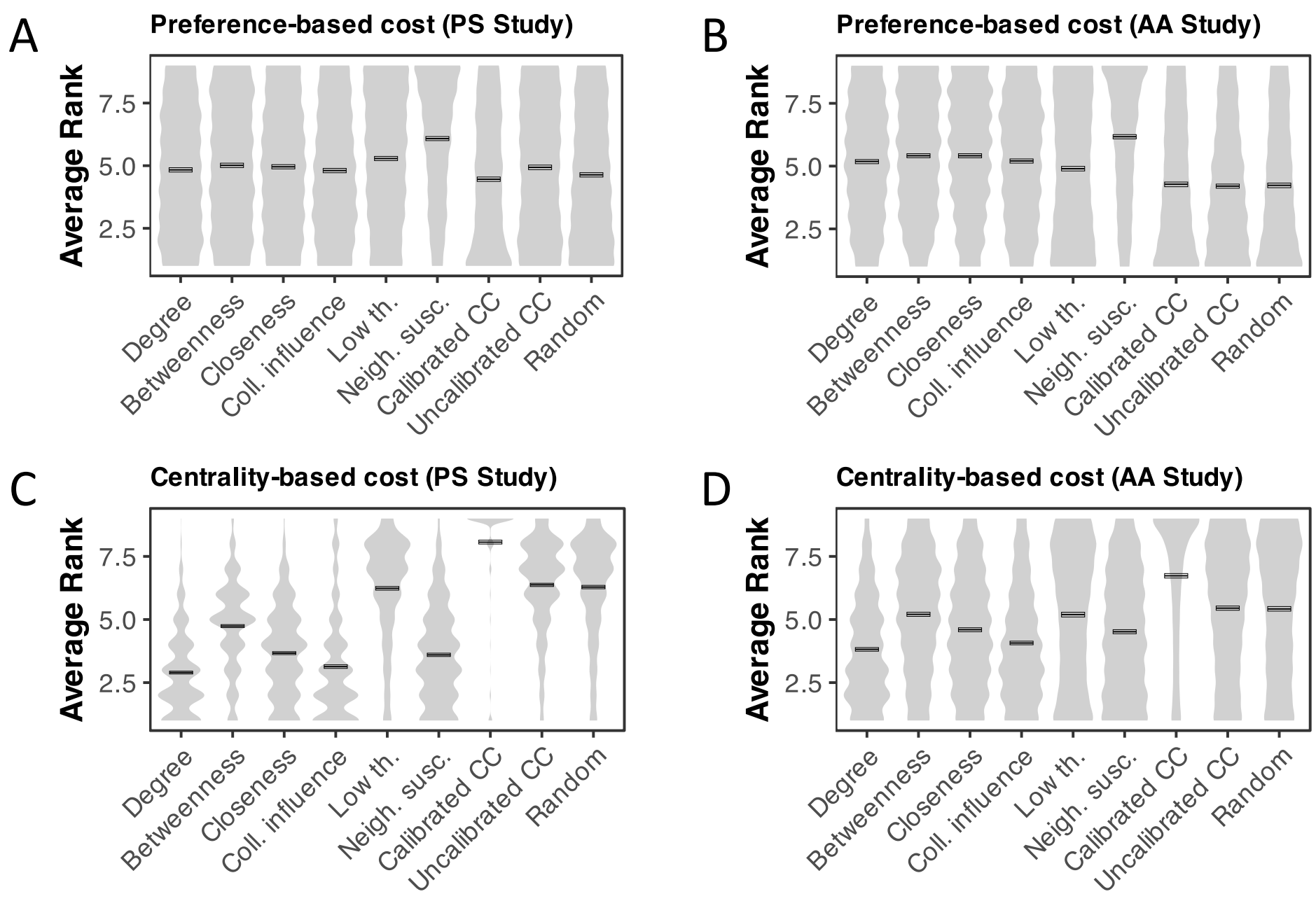

Fig. S13. **Relative performance of seeding policies for a smaller seed set size ($z = 0.0125$), according to the mean rank metric.** **(A, B)** Relative performance of seeding policies under a preference–based cost structure, measured through the mean rank metric used in main text, for the policy support experiment and the app adoption experiment, respectively. The neighborhood susceptibility policy based on the estimated thresholds significantly outperforms the other policies (one-tailed Wilcoxon signed-rank test, all p-values smaller than $p = 0.001$, the smallest effect size is $r = 0.19$ in the AA Study in the comparison against the closeness centrality). **(C, D)** Relative performance of seeding policies under a centrality–based cost structure for the policy support experiment and the app adoption experiment, respectively. The complex centrality policy based on the estimated thresholds significantly outperforms the other policies (one-tailed Wilcoxon signed-rank test, all p-values smaller than $p = 0.001$, the smallest effect size is $r = 0.32$ in the AA Study in the comparison against the uncalibrated complex centrality). Overall the results are in qualitative agreement with those obtained with $z = 0.025$ (Fig. 2 in the main text). In all the panels, crossbars represent the mean together with the 95% confidence intervals over 3,240 runs.

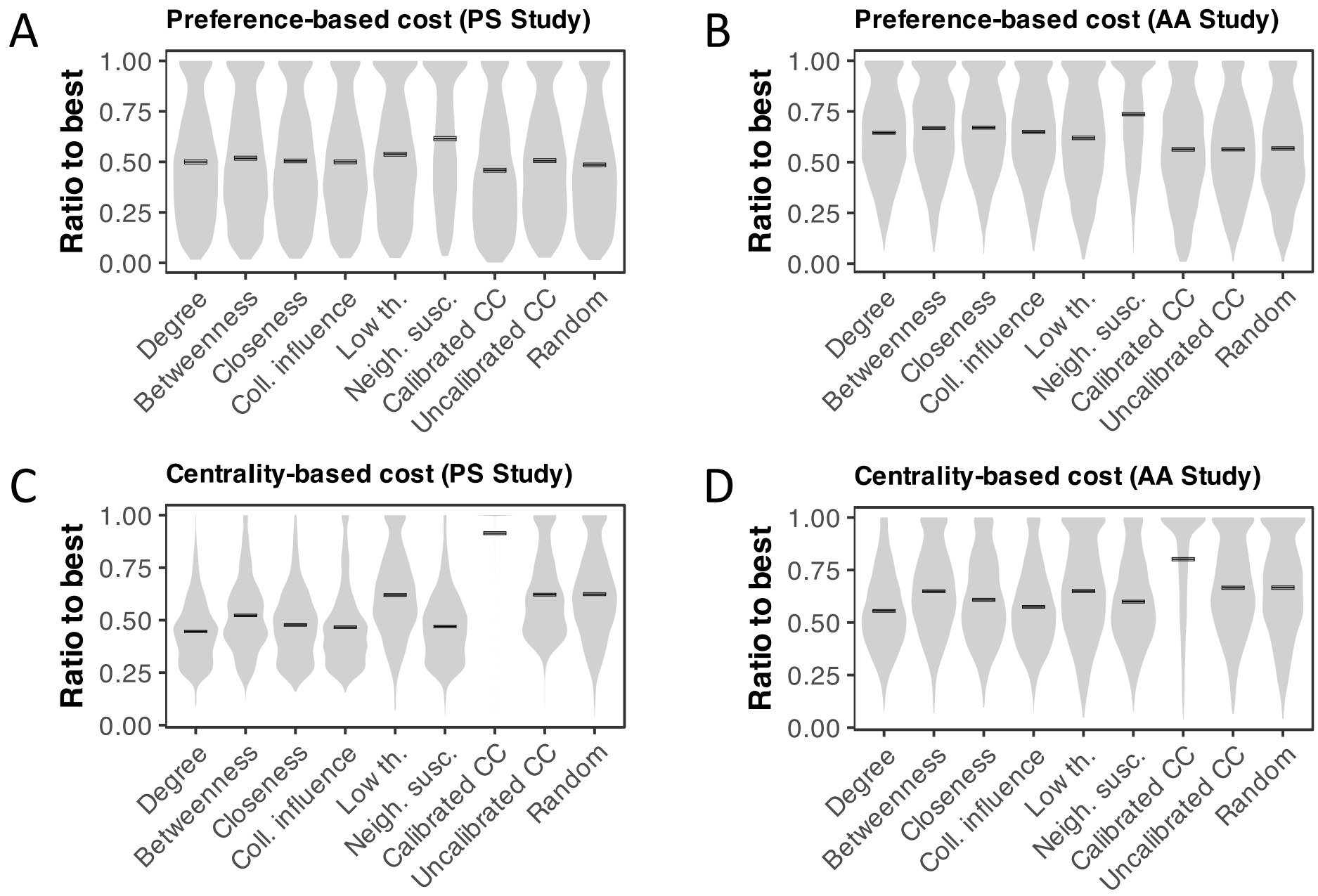

Fig. S14. **Relative performance of seeding policies for a smaller seed set size ($z = 0.0125$), according to the ratio-to-best metric. (A, B)** Relative performance of seeding policies under a preference–based cost structure, measured through the ratio-to-best metric defined above, for the policy support experiment and the app adoption experiment, respectively. The neighborhood susceptibility policy based on the estimated thresholds significantly outperforms the other policies (one-tailed Wilcoxon signed-rank test, all p-values smaller than $p = 0.001$, the smallest effect size is $r = 0.20$ in the comparison against the low-threshold policy in the PS Study). **(C, D)** Relative performance of seeding policies under a centrality–based cost structure for the policy support experiment and the app adoption experiment, respectively. The complex centrality policy based on the estimated thresholds significantly outperforms the other policies (one-tailed Wilcoxon signed-rank test, all p-values are smaller than $p = 0.001$, the smallest effect size is $r = 0.37$ in the AA Study in the comparison against the random policy). Overall the results are in qualitative agreement with those obtained with $z = 0.025$ (Fig. 2 in main the text). In all the panels, crossbars represent the mean together with the 95% confidence intervals over $3,240$ runs.

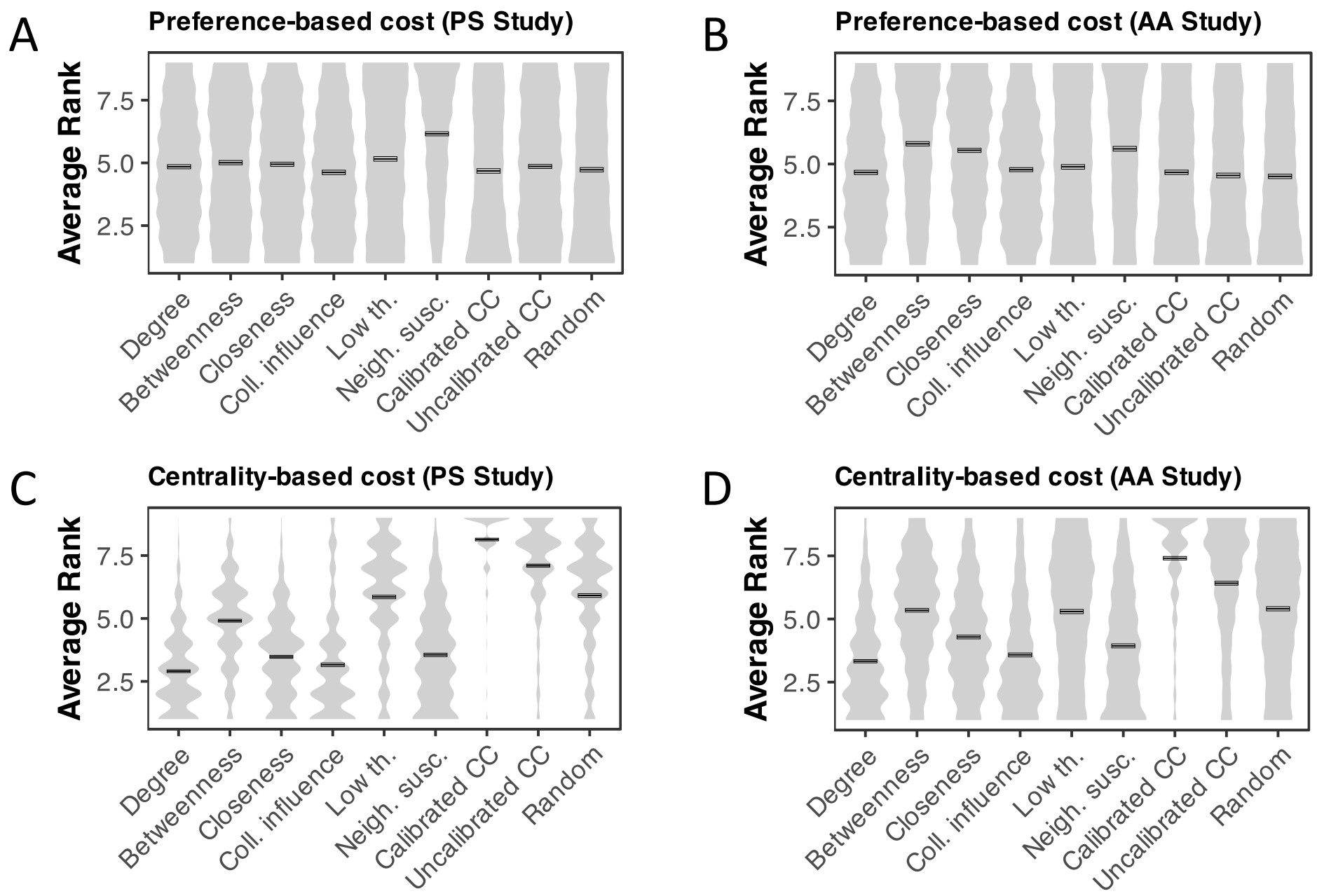

Fig. S15. **Relative performance of seeding policies for a larger seed set size ($z = 0.05$), according to the mean rank metric. (A, B)** Relative performance of seeding policies under a preference–based cost structure, measured through the mean rank metric used in main text, for the PS Study and the AA Study, respectively. In the simulations calibrated with the AA Study results, the neighborhood susceptibility policy outperforms all the other metrics except the betweenness and closeness centralities (one-tailed Wilcoxon signed-rank test, all p-values are smaller than $p = 0.001$, the smallest effect size is $r = 0.18$ in the comparison against the low threshold): For larger seed sets, the estimated thresholds remain informative but no longer fully sufficient to identify more effective focal seeds than the betweenness and closeness centrality. **(C, D)** Relative performance of seeding policies under a centrality–based cost structure for the policy support experiment and the app adoption experiment, respectively. The complex centrality policy based on the estimated thresholds significantly outperforms the other policies (one-tailed Wilcoxon signed-rank test, all p-values are smaller than $p = 0.001$, the smallest effect size is $r = 0.32$ in the AA Study in the comparison against the uncalibrated complex centrality); this result is in qualitative agreement with those obtained with $z = 0.025$ (Fig. 2 in the main text). In all the panels, crossbars represent the mean together with the 95% confidence intervals over $3,240$ runs.

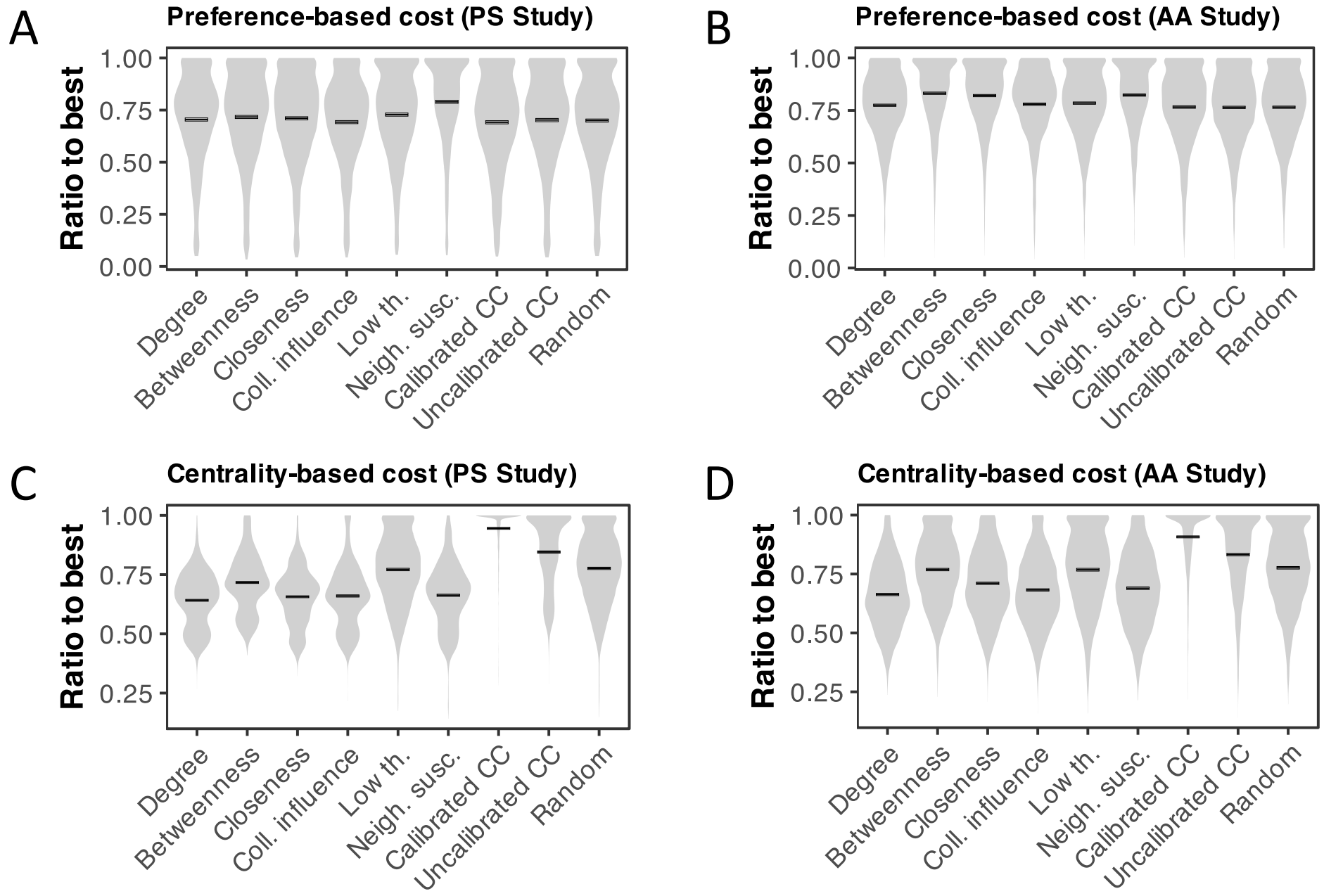

Fig. S16. **Relative performance of seeding policies for a larger seed set size ($z = 0.05$), according to the ratio-to-best metric. (A, B)** Relative performance of seeding policies under a preference–based cost structure, measured through the ratio-to-best metric defined above, for the policy support experiment and the app adoption experiment, respectively. Again, in the simulations calibrated with the AA study results, the neighborhood susceptibility policy outperforms all the other metrics except the betweenness and closeness centralities (one-tailed Wilcoxon signed-rank test, all p-values are smaller than $p = 0.001$, the smallest effect size is $r = 0.19$ in the comparison against the low threshold): For larger seed sets, the estimated thresholds remain informative but no longer fully sufficient to identify more effective focal seeds than the betweenness and closeness. **(C, D)** Relative performance of seeding policies under a centrality–based cost structure for the policy support experiment and the app adoption experiment, respectively. The complex centrality policy based on the estimated thresholds significantly outperforms the other policies (one-tailed Wilcoxon signed-rank test, all p-values are smaller than $p = 0.001$, the smallest effect size is $r = 0.36$ in the AA Study in the comparison against the uncalibrated complex centrality). In all the panels, crossbars represent the mean together with the 95% confidence intervals over $3,240$ runs.

C. Results in scenarios with partial threshold data

We examine the relative performance of the seeding policies in scenarios where only a portion of the nodes have been surveyed, and a fraction of the thresholds is missing. For the behavior-based policies, we narrow our focus to the low-threshold and neighborhood susceptibility policies (and consequently to the preference-based cost where they outperform the other metrics), as the calibrated complex centrality requires all the thresholds as input. For simplicity, we assume that when selecting the seeds according to these two policies, the social change practitioner neglects completely information from the nodes that she has not been able to survey. Therefore in this scenario, the focal seed according to the low-threshold policy is the one with the lowest threshold among the surveyed nodes; the focal seed according to the neighborhood susceptibility policy is the one with the largest number of low-threshold neighbors among the surveyed nodes.

We find that for both experiments, the neighborhood susceptibility policy maintains an advantage over the other policies even where a sizeable fraction of the nodes (e.g., 10% or 25%) have not been surveyed (see Supplementary Figs. S17-S18). When data is highly incomplete (50% or more non-surveyed nodes), the significant advantage of the neighborhood susceptibility policy is still robust in the PS experiment, but not in the AA experiment (see Supplementary Figs. S19-S21). This is arguably due to the larger number of low-threshold nodes in the PS experiment, which makes the identification of low-threshold nodes simpler even with highly incomplete data. Note that, for simplicity, we restricted the set of potential seeds to the surveyed nodes, even in scenarios where they only constitute a small portion of the population. An alternative approach to deal with data incompleteness would rely on threshold measurements at group level. The Hierarchical Bayes algorithm adopted here [4] can indeed deliver group-level parameter distributions (where the groups are determined, e.g., based on gender, ethnicity or any relevant discrete covariates), from which representative individuals can be drawn and their thresholds used as input for the behavior-based seeding algorithms. This possibility, which we leave to future research, might further improve the detection of nodes with high neighborhood susceptibility and potentially enable an estimation of the complex centrality in scenarios with incomplete data.

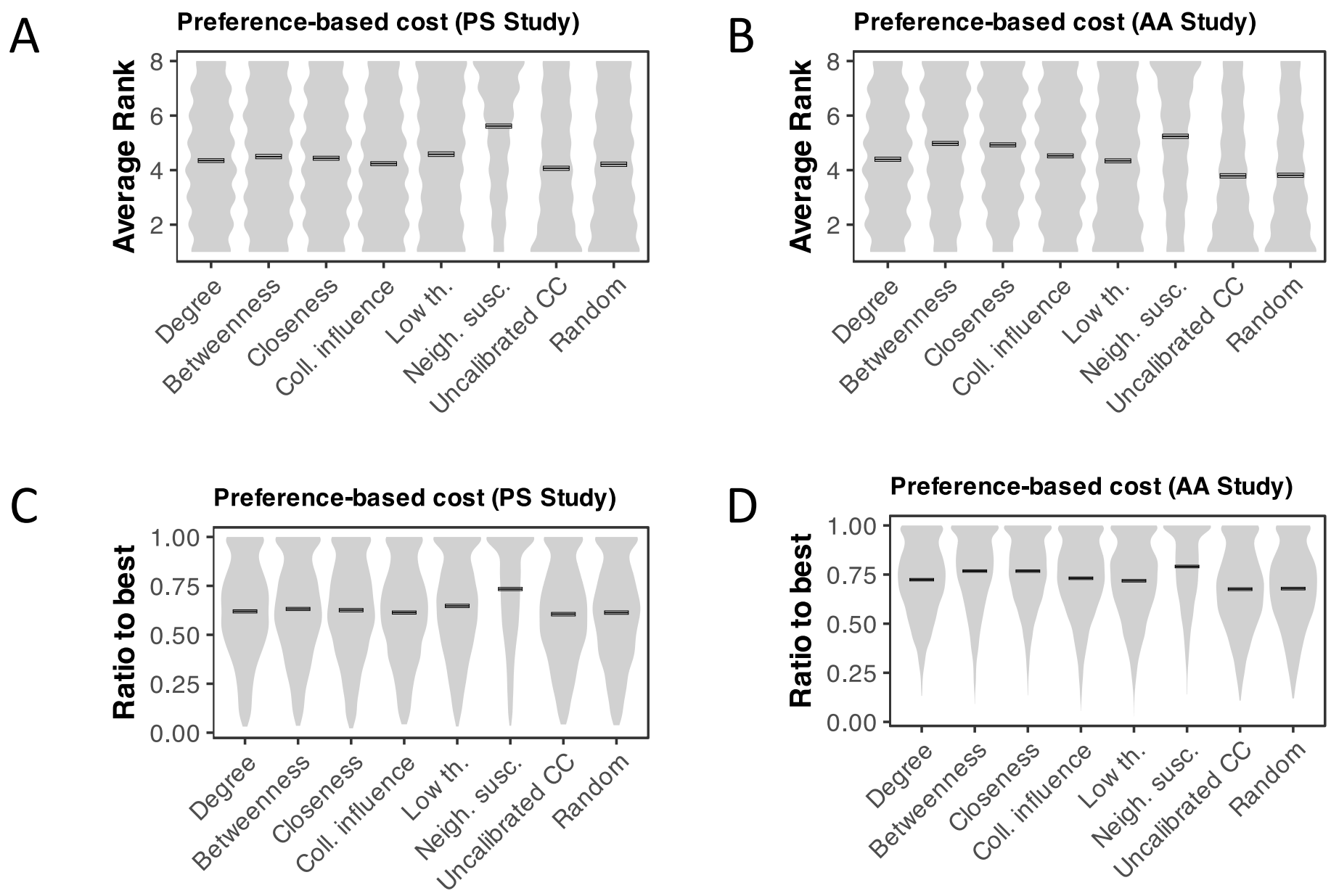

Fig. S17. **Relative performance of seeding policies when 90% of the nodes are surveyed.** Relative performance of seeding policies under a preference–based cost structure, measured through the mean rank metric used in main text **(A, B)** and through the ratio-to-best metric defined above **(C, D)** for the policy support experiment and the app adoption experiment, respectively. The neighborhood susceptibility policy based on the estimated thresholds outperforms the other policies (one-tailed Wilcoxon signed-rank test, all p-values are smaller than $p = 0.001$, the smallest effect size is $r = 0.07$ in the AA Study in the comparison against the betweenness centrality for the average rank metric). In all the panels, crossbars represent the mean together with the 95% confidence intervals over $3,240$ runs.

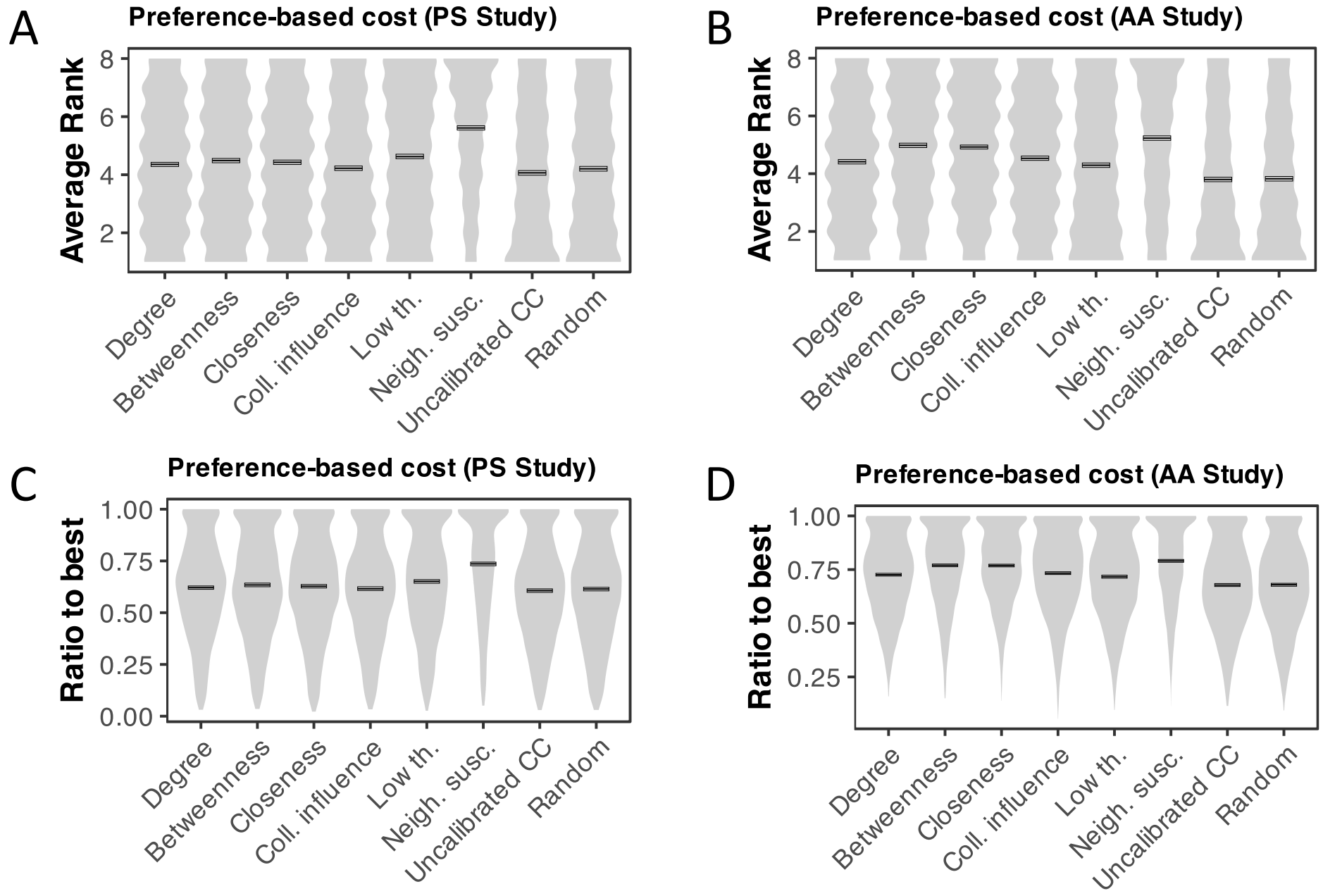

Fig. S18. **Relative performance of seeding policies when** 75% **of the nodes are surveyed.** Relative performance of seeding policies under a preference–based cost structure, measured through the mean rank metric used in main text **(A, B)** and through the ratio-to-best metric defined above **(C, D)** for the policy support experiment and the app adoption experiment, respectively. The neighborhood susceptibility policy based on the estimated thresholds outperforms the other policies (one-tailed Wilcoxon signed-rank test, all p-values are smaller than $p = 0.001$, the smallest effect size is $r = 0.07$ in the AA Study in the comparison against the betweenness centrality for the average rank metric). In all the panels, crossbars represent the mean together with the 95% confidence intervals over 3, 240 runs.

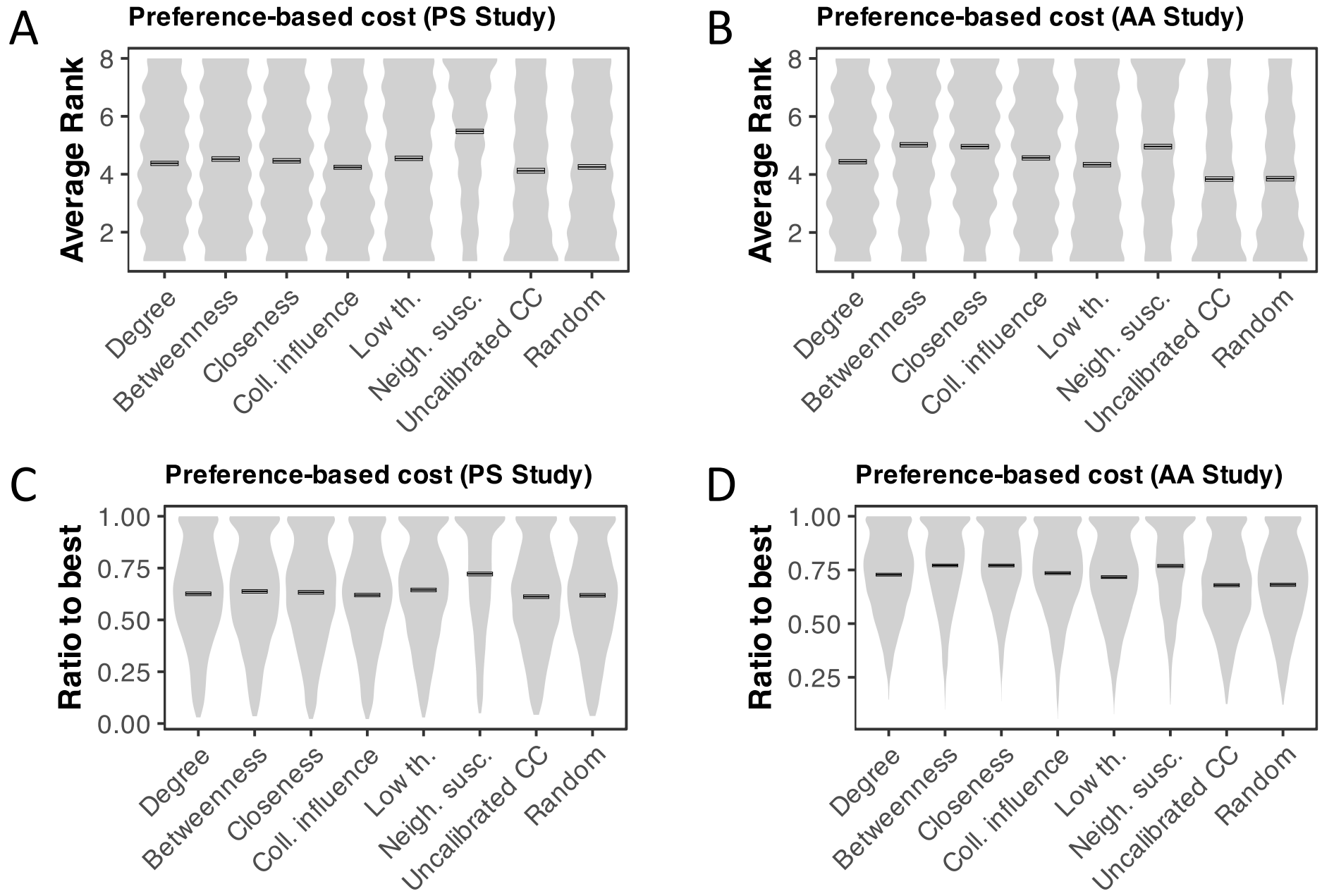

Fig. S19. **Relative performance of seeding policies when** 50% **of the nodes are surveyed.** Relative performance of seeding policies under a preference–based cost structure, measured through the mean rank metric used in main text **(A, B)** and through the ratio-to-best metric defined above **(C, D)** for the policy support experiment and the app adoption experiment, respectively. In the simulations calibrated with the AA experiment, the neighborhood susceptibility policy based on the estimated thresholds no longer outpeforms the betweenness and closeness centrality (one-tailed Wilcoxon signed-rank test, all p-values are larger than $p = 0.48$, the largest effect size is $r = 0.0007$ in the comparison against the closeness centrality for the average rank metric). In all the panels, crossbars represent the mean together with the 95% confidence intervals over 3,240 runs.

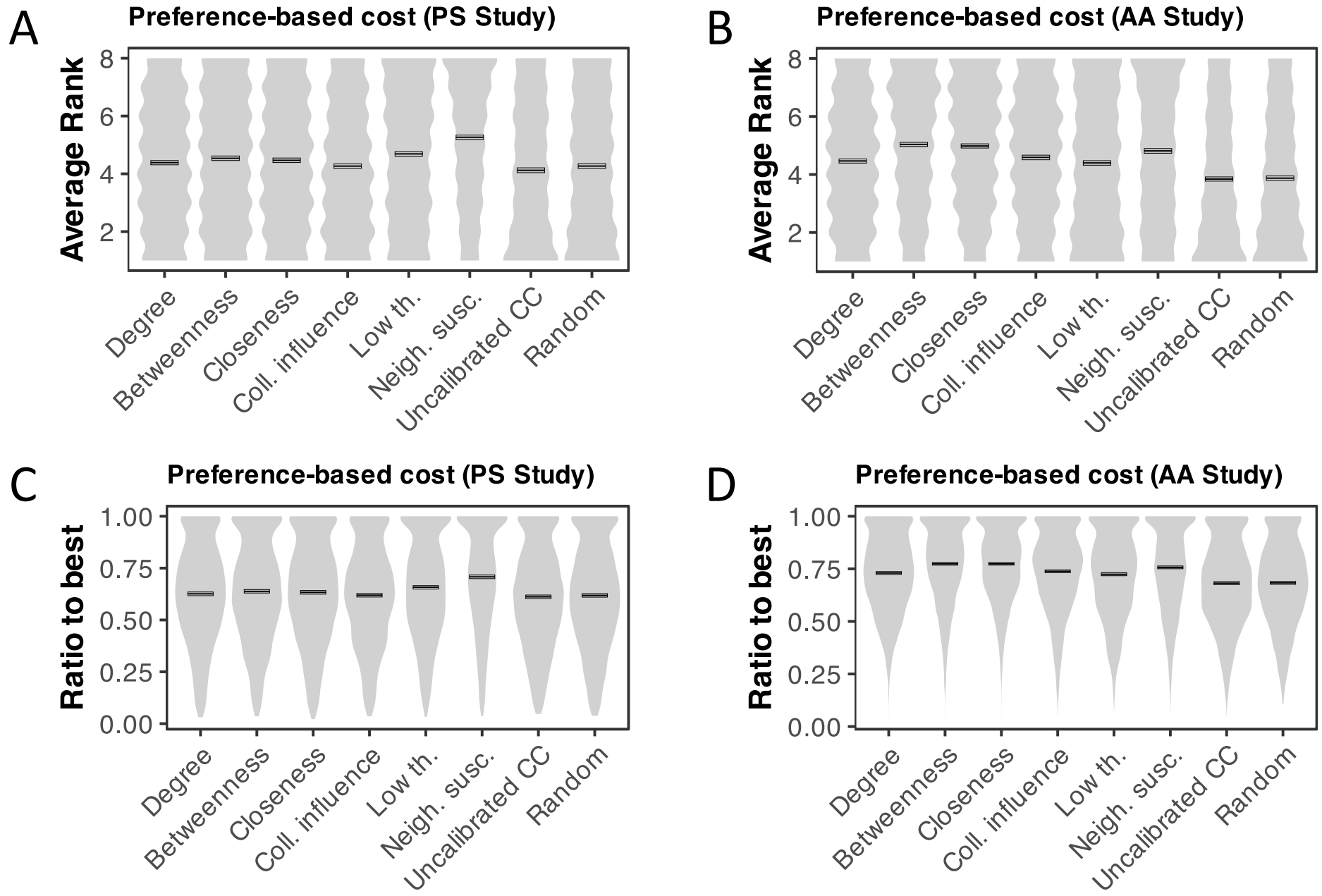

Fig. S20. **Relative performance of seeding policies when** 25% **of the nodes are surveyed.** Relative performance of seeding policies under a preference–based cost structure, measured through the mean rank metric used in main text **(A, B)** and through the ratio-to-best metric defined above **(C, D)** for the policy support experiment and the app adoption experiment, respectively. In the simulations calibrated with the AA experiment, the neighborhood susceptibility policy based on the estimated thresholds no longer outperforms betweenness and closeness centrality (one-tailed Wilcoxon signed-rank test, all p-values are larger than $p = 0.997$, the largest effect size is $r = 0.07$ in the comparison against the betwenness centrality for the ratio-to-best metric). In all the panels, crossbars represent the mean together with the 95% confidence intervals over 3,240 runs.

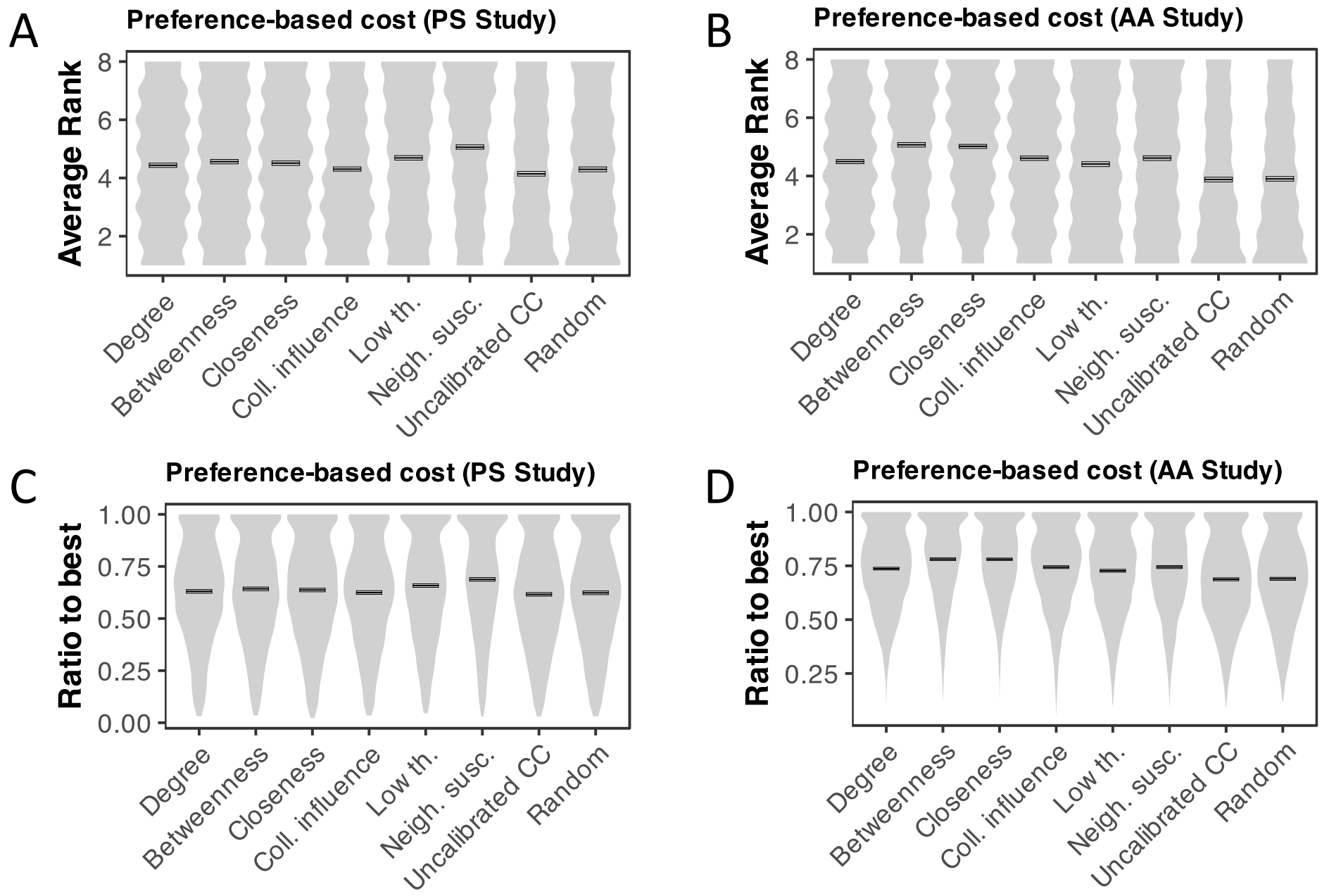

Fig. S21. **Relative performance of seeding policies when** 10% **of the nodes are surveyed.** Relative performance of seeding policies under a preference–based cost structure, measured through the mean rank metric used in main text **(A, B)** and through the ratio-to-best metric defined above **(C, D)** for the policy support experiment and the app adoption experiment, respectively. In the simulations calibrated with the AA experiment, the neighborhood susceptibility policy based on the estimated thresholds no longer outperforms betweeness centrality, closeness centrality and collective influence (one-tailed Wilcoxon signed-rank test, all p-values are larger than $p = 0.47$, the largest effect size is $r = 0.14$ in the comparison against the betweenness centrality for the ratio-to-best metric). In all the panels, crossbars represent the mean together with the 95% confidence intervals over $3,240$ runs.

D. Effect of prior specification on the identification of effective seeds

In Supplementary Note S3 C we showed the estimated thresholds are robust to the priors chosen in the HB estimation (as long as priors are sensible). We now examine if the small detected differences in the estimated thresholds across different priors might still lead to substantial differences in the performance of seeding strategies informed by the different estimated thresholds. To this end, we replicate the simulation procedure described in the Methods section of the main paper, using the adoption thresholds estimated under the four prior specifications, based on the dataset with 15 choice tasks per respondent (see Supplementary Note S3 C). The results are shown in Figs. S22 (PS Study) and S23 (AA Study).

For both studies, across all prior specifications, the results are in qualitative agreement with those in Fig. 2 in the main text. In the preference-based cost scenario, the neighborhood susceptibility remains the most effective seeding strategy in all cases except for the uninformative prior in the AA Study (one-tailed Wilcoxon signed-rank test, all p-values are smaller than $p = 0.02$, the smallest effect size is $r = 0.03$ in the comparison with betweenness centrality in the AA Study for the loose prior). In the centrality-based cost, the calibrated complex centrality performs best (one-tailed Wilcoxon signed-rank test, all p-values are smaller than $p = 0.001$, the smallest effect size is $r = 0.33$ in the comparison with the uncalibrated complex centrality in the AA Study for the uninformative prior). This consistency can be attributed to the relatively small differences in threshold estimation accuracy (Fig. S9B,E) and the high correlation between true and estimated thresholds across all priors (Fig. S9C,F), since the adoption threshold is the only estimated parameter used for the seed identification.

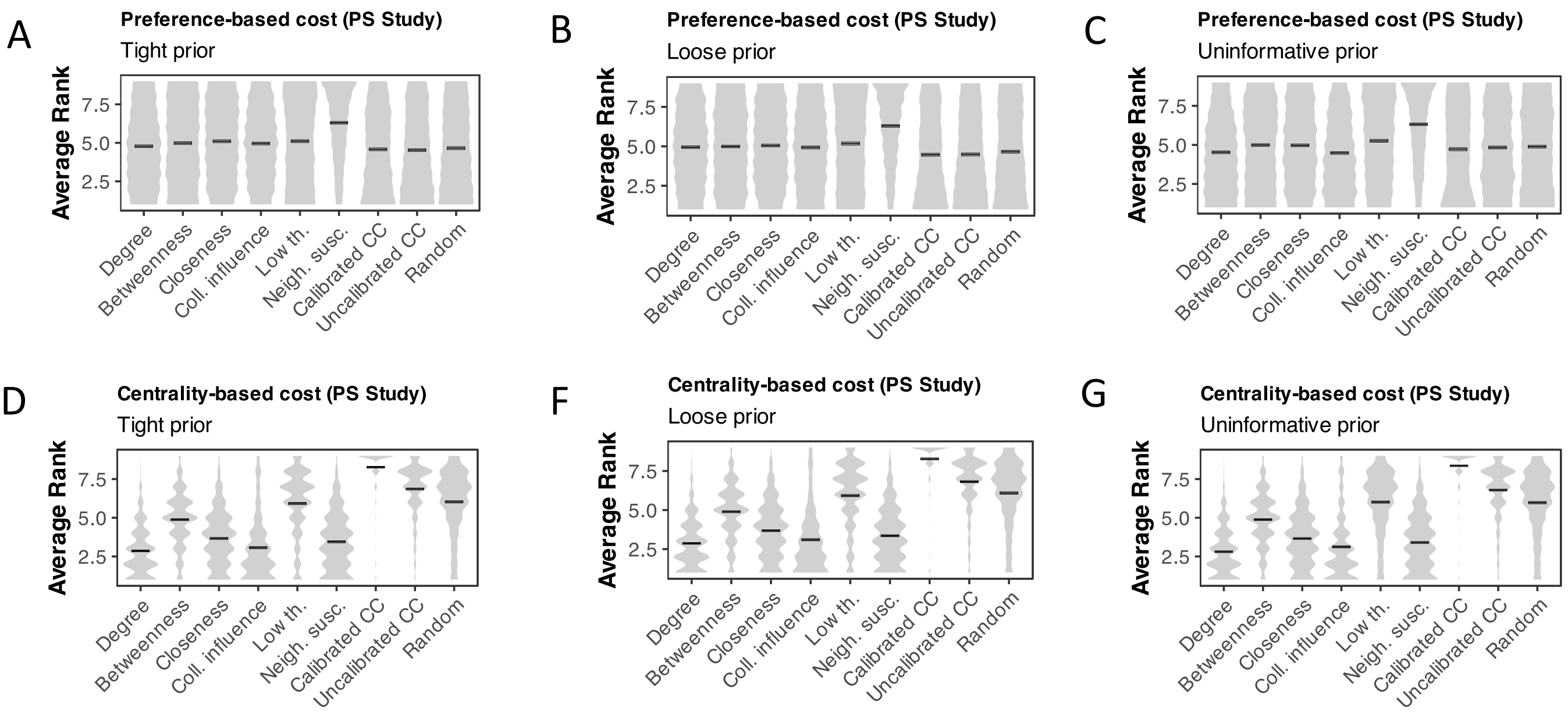

Fig. S22. **Relative performance of seeding policies in the PS Study under different priors and cost structures according to the mean rank metric.** Overall the results are in qualitative agreement with those obtained in Fig. 2 in the main text.

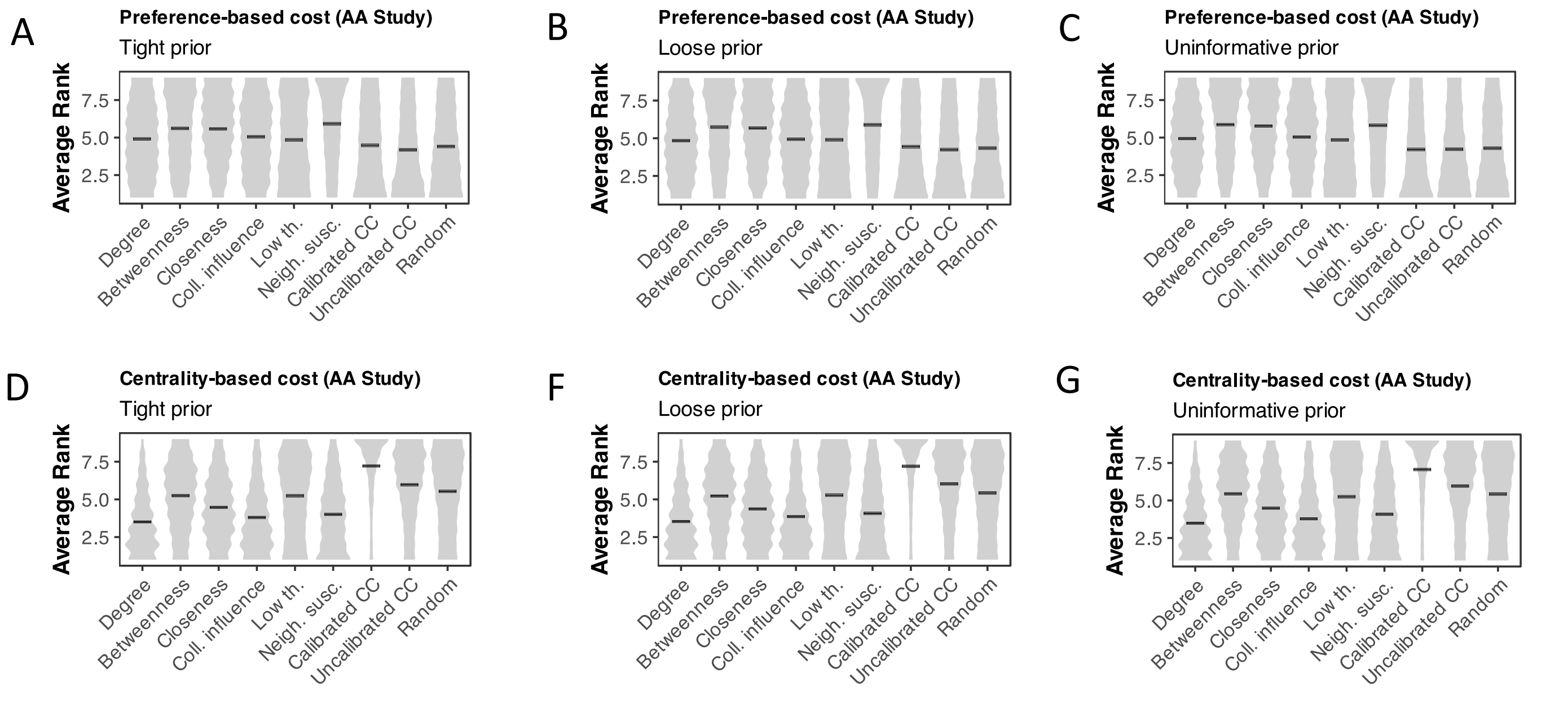

Fig. S23. **Relative performance of seeding policies in the AA Study under different priors and cost structures according to the mean rank metric.** Overall the results are in qualitative agreement with those obtained in Fig. 2 in the main text.

S6. RECOVERING SOCIAL SPREADING MODELS FROM CHOICE THEORY

In main text, we assumed a threshold-based diffusion model, aligned with the social contagion literature [30–32]. Here we show that a utility-based framework under different assumptions that those adopted in main text still recovers interpretable social spreading models – e.g., the traditional Bass diffusion [33] and logistic diffusion models [34] – which could be investigated in future empirical research.

In this section, we move from a deterministic choice model to a stochastic one for binary choices (here the binary choice of interest is adopting vs. not adopting the new product or behavior). Following standard random utility theory [3, 35], a stochastic choice model is achieved by introducing a noise term into the utility functions. The relative importance of the noise compared to the utility's deterministic components is ruled by a clarity parameter: the lower the clarity, the higher the importance of the noise term [34]. In Section S6 B, we show that by making the same assumptions about the distribution of noise as those used for estimation (see Methods), we obtain a logistic diffusion model, which can be interpreted as a noisy threshold-based diffusion model [36]. The threshold model emerges as the high-clarity limit (or zero-temperature limit, in the statistical physics terminology [37]) of the resulting model. Notably, in the low-clarity limit, where noise dominates, the logistic model approximates the widely-used Bass model of new product diffusion [33] (see Supplementary Note S6 C).

A. General framework

In main text, we considered a simple utility-based choice model where an individual adopts product i as soon as the utility from adopting product i, U_{ni}, exceeds the utility from not adopting, $U_n^{(0)}$, which led to a simple formula linking individual threshold and utility coefficients (Eq 1 in main text). Within discrete choice theory, random utility models [3, 35] provide a natural framework for estimating individual utilities from empirical data (which we already leveraged in main text, see Methods) and for modeling noisy choices, which is the focus of this Section.

In general terms, discrete choice theory [3] considers behavioral models $y = h(x, \epsilon)$ that describes how an individual-level choice y is determined by some observable properties x and some unobservable ones, ϵ, where ϵ follows a certain distribution, $\epsilon \sim f(\epsilon)$. The goal is typically to obtain the probability that an individual makes choice y, $\Pi(y|x)$, given the observable properties x. The theory assumes that an individual n chooses among I alternatives $i \in \{1, \dots, I\}$ in such a way to maximize her utility [3]

$$U_{ni} = \lambda\, U^*_{ni} + \epsilon_{ni}, \tag{S1}$$

where U^*_{ni} depends on observable properties of individual n and alternative i; ϵ_{ni} is an error term drawn from a certain distribution f: $\epsilon_{ni} \sim f(\epsilon_{ni})$; λ denotes a scale parameter [3] also referred to as clarity parameter [34]. One can show that if $\epsilon_{i\alpha}$s are i.i.d. variables extracted from an extreme value distribution (Gumbel, $f(\epsilon) = \exp(-\epsilon)\exp(-\exp(-\epsilon))$, then the probability that individual n chooses alternative i is simply [3]

$$\Pi_{ni} = \frac{\exp(\lambda\, U^*_{ni})}{\sum_{j=1}^{I} \exp(\lambda\, U^*_{nj})}. \tag{S2}$$

Eq. (S2) can be also derived via statistical physics methods, by assuming that an individual maximizes her expected utility plus the variety of her available options, represented by the entropy of the choice probability distribution [37]. For a *binary choice* between not adopting and adopting a given product i, we simplify the notation and denote by Π_{ni} and $\bar{\Pi}_{ni} = 1 - \Pi_{ni}$ the probabilities that n adopts and does not adopt product i, respectively. We have:

$$\Pi_{ni} = \frac{1}{1 + \exp(\lambda\, (\bar{U}^*_{ni} - U^*_{ni}))}, \tag{S3}$$

where we denoted with U^*_{ni} and $\bar{U}^*_{ni}$ the systematic utilities from adopting and not adopting, respectively. In the following, we shall use these results to derive collective adoption models. Clarity parameter λ rules the relative importance of the systematic and stochastic components; in the statistical physics language, it can be referred to as inverse temperature [37]. We can consider two regimes: high clarity ($\lambda \gg 1$) and low clarity ($\lambda \ll 1$). In the binary case, in the high-clarity regime,

$$\Pi_{ni} = \frac{1}{1 + \exp(\lambda(\bar{U}^*_{ni} - U^*_{ni}))} \simeq \Theta(U^*_{ni} - \bar{U}^*_{ni}), \tag{S4}$$

where Θ is the Heaviside function. This represents the threshold rule that has been used in main text to reinterpret the threshold-based diffusion model. In the low-clarity regime, we use the property that for $\epsilon \sim 0$, one has $(1+\exp\epsilon)^{-1} \simeq 1/2 - \epsilon/4$. Therefore,

$$\Pi_{ni} \simeq \frac{1}{2} + \frac{\lambda}{4}(U^*_{ni} - \bar{U}^*_{ni}) + \mathcal{O}(\lambda^2). \tag{S5}$$

The threshold-like probability obtained in the high-clarity regime and the linear probability obtained in the low-clarity regime can be linked to the adoption probabilities for a complex and a simple social contagion model, respectively, as shown below.

B. High clarity regime: Threshold model

We discuss here we show how the threshold model analyzed in the main text emerges naturally as the high-clarity limit of the random utility model introduced above. We further demonstrate how the utility-based framework provides intuitive expressions for the cost associated with successfully targeting a given node. Finally, we examine the influence of two-way inter-attribute interactions on the analytical form of the adoption threshold.

Linking partworth utilities and the adoption threshold. Consider a scenario where an individual makes a binary choice about whether to adopt product i. As in the main text, suppose that the systematic component of her utility from adopting product i is:

$$\bar{U}^*_{ni} = \lambda \left(\sum_a \beta_{na}\, x_{ia} + \gamma_n\, s_{ni} \right) = \lambda\, (U^{(A)}_{ni} + \gamma_n\, s_{ni}). \tag{S6}$$

Suppose that the utility from not adopting is fixed, $\bar{U}^*_n = U^{(0)}_n$, where $U^{(0)}_n$ could represent the price, the risk from adopting, or some product-specific cost of adopting. From Eq. (S4), it follows that

$$\Pi_{ni} = \frac{1}{1 + \exp\left(\lambda\, (U^{(0)}_n - U^{(A)}_{ni} - \gamma_n\, s_{ni})\right)}. \tag{S7}$$

For $U^{(A)}_{ni} = 0$, only social signals affect individuals' adoption decisions, and we recover the logistic model referred to as burden of social proof model [34]. In the limit of large λ (high clarity), $\Pi_{ni} = 1$ if and only if

$$U^{(0)}_n - U^{(A)}_{ni} - \gamma_n\, s_{ni} > 0, \tag{S8}$$

which can be rewritten as

$$s_{ni} > \gamma_n^{-1}(U^{(0)}_n - U^{(A)}_{ni}) = \tau_{ni}, \tag{S9}$$

from which Eq 1 in the main text.

Motivating the cost functions. The proposed interpretation of the threshold model also enables a natural interpretation of the cost or effort needed to persuade an individual to adopt a new product in the absence of social signal ($s_{ni} = 0$), which is the case at the seeding stage, when the diffusion has not started yet. In this case, $U^{(S)} = 0$; one can interpret the cost of targeting as the utility increase needed such that $U_{ni} > \bar{U}_{ni}$ is verified. We add the effort term, $U^{(E)}_{ni}$, to the utility as

$$U^*_{ni}(s_{ni} = 0) = U^{(A)}_{ni} + U^{(E)}_{ni}. \tag{S10}$$

Although efforts to incentivize adoption can take various forms [38], to fix ideas, we interpret $U^{(E)}_{ni}$ as the result of a monetary incentive offered by the practitioner to individual n in order to persuade her to adopt i. For simplicity, we assume $U^{(E)}_{ni} = \mu_n\, c_{ni}$, where c_{ni} denotes the cost of targeting n individual to promote i, and μ_n denotes n's sensitivity to the targeting offer. Under these assumption, in the beginning of the diffusion, n adopts only if $U^{(A)}_{ni} + U^{(E)}_{ni} \geq U^{(0)}_n$; the minimal incentive for this condition to hold is

$$\kappa_{ni} = \mu_n^{-1}(U^{(0)}_n - U^{(A)}_{ni}). \tag{S11}$$

This result is intuitive: the cost to effectively target n for product i is higher the higher the resistance ($R_{ni} = U_n^{(0)} - U_{ni}^{(A)}$) between her status-quo utility and her attribute utility. It aligns with the literature on social norms, which posits that successfully targeting initially-resistant agents requires higher incentive [39–41]. In main text, we set $\mu_n = 1$. Note that Eq. (S12) outputs negative values of κ_{ni} for individuals with $R_{ni} < 0$; we assume that the effective cost of successfully targeting these individuals is zero, which leads to

$$\kappa_{ni} = \max\Big\{U_n^{(0)} - U_{ni}^{(A)}, 0\Big\}. \tag{S12}$$

Finally, we consider a fixed implementation cost per intervention, c_0, which leads to the final expression for the cost in the preference-based scenario:

$$C_i(\mathcal{S}) = c_0 + \sum_{n\in\mathcal{S}} \max\Big\{U_n^{(0)} - U_{ni}^{(A)}, 0\Big\} \tag{S13}$$

which is Eq. 5 in main text. In main text, we also consider a scenario where the cost of targeting depends on the centrality of the prospect target, which better aligns with the literature on influencers in social media [42, 43]. In the centrality-based cost scenario, the seeding cost is defined as

$$C_i(\mathcal{S}) = c_0 + \sum_{n\in\mathcal{S}} d_n, \tag{S14}$$

where d_n denotes n's degree, which is Eq. 6 in main text, and c_0 denotes again a fixed implementation cost per intervention. In this scenario, the cost is independent of n's utility.

Adoption threshold in presence of interactions. In main text, Eq. (S6) for the systematic utility assumes both additivity and separability of the partworths. One can formally relax these assumptions in various ways. A general model that includes two-way interactions between all partworth utilities is:

$$\bar{U}_{ni}^{*} = \lambda \left(\sum_a \beta_{na}\, x_{ia} + \sum_{a,b} \beta'_{nab}\, x_{ia}\, x_{ib} + \gamma_n\, s_{ni} + \sum_a \gamma'_{na}\, x_{ia}\, s_{ni} \right) \tag{S15}$$

where β'_{nab} captures the interaction between attributes a and b; γ'_{na} captures the interaction between attribute a and the social signal. Eq. S15 can be rewritten as:

$$\bar{U}_{ni}^{*} = \lambda\, (\tilde{U}_{ni}^{(A)} + \tilde{\gamma}_n\, s_{ni}), \tag{S16}$$

where we defined the interacting attribute utility,

$$\tilde{U}_{ni}^{(A)} = \sum_a \beta_{na}\, x_{ia} + \sum_{a,b} \beta'_{nab}\, x_{ia}\, x_{ib}, \tag{S17}$$

and the interacting susceptibility to social influence,

$$\tilde{\gamma}_{ni} = \gamma_n + \sum_a \gamma'_{na}\, x_{ia}. \tag{S18}$$

With these definitions, if $\tilde{\gamma}_n > 0$, one can obtain again an expression for threshold, $\tilde{\tau}_{ni}$ from the condition $U_{ni}^{*} \geq \overline{U}_{ni}^{*}$, which leads to the following expression:

$$\tilde{\tau}_{ni} = \frac{U_n^{(0)} - \tilde{U}_{ni}^{(A)}}{\tilde{\gamma}_{ni}}, \tag{S19}$$

which is similar to Eq. 1 in main text, with the interaction-free attribute utility and susceptibility being replaced by the interacting ones, $\tilde{U}_{ni}^{(A)}$ and $\tilde{\gamma}_{ni}$. We conclude that as long as $\tilde{\gamma}_n > 0$, the threshold is well-defined (in a similar sense as for the model without interactions). We leave for future research explorations of how the thresholds might be estimated in presence of significant interaction effects in specific contexts, which might require tailored designs or estimation methods.

C. Low clarity regime: Bass model

We show here how the random utility model defined above, in the low-clarity limit, leads to the well-studied Bass model of new product diffusion, and we link the coefficients of the Bass model to the utility and clarity parameters. The Bass model assumes that faced with the binary choice of whether to adopt a new product or not, the probability that individual n adopts at a given time t is $\Pi_n(t) = p_n + q_n\, s_n(t)$, where $s_n(t)$ is the social signal received by n at time t about the product, and p, q denote the innovation and innovation coefficient, respectively; for simplicity in this section, we omit the product subscript (i) from the notation. In a fully-connected network, $s_n(t) = s(t)$ denotes n's cumulative number of adopters at time t, which in a homogeneous preference scenario $(p_n = p, q_n = q)$, leads to the original formulation of the Bass model [33]. When the potential adopters are embedded in a non-trivial network topology, $s_n(t)$ can represent the fraction of n's social contacts who already adopted the product [25]. Following Centola and Macy's terminology [31], the Bass model can be interpreted as a *simple contagion model* as individuals have a non-zero probability of adopting even if only one of their social contacts already adopted (even when $p = 0$).

We show that the Bass model can be reinterpreted in terms of individual-level utility functions. Similarly as above, we assume that individual n's utility from adopting the new product at a given time t is $U^*_n(t) = \lambda\,(U^{(A)}_n + \gamma_n\, s_n(t))$, where $U^{(A)}_n$ could be expressed, as in the main text, as a linear combination of the product attributes; again, γ_n denotes the marginal utility of social signals for n. We assume again that the status-quo utility from not adopting is fixed: $\bar{U}^*_n(t) = U^{(0)}_n$, where $U^{(0)}_n$ is a parameter that could represent the price or risk associated with the adoption. In the low-clarity regime Eq. (S5), we obtain that Π_n follows the Bass equation with innovation parameter $p_n = (1-\lambda\,(U^{(0)}_n - U^{(A)}_n))/2 = 1/2 - \lambda\, R_n/4 = 1/2 - R_n/T$ and imitation parameter $q_n = \lambda\,\gamma_n/4 = \gamma_n/T$, where we defined the temperature parameter [35, 37] as $T = 4/\lambda$. The role of temperature parameter T influences the Bass model's parameter: In the infinite-temperature limit, the choice is only determined by the stochastic component of the utility. In this limit, the innovation parameter reflects a coin flip (1/2 probability) and the social signal plays no role (zero imitation parameter). As the temperature is large (necessary to derive the linear adoption probability) but finite, p_n deviates from $1/2$ and $q_n > 0$. The finite-temperature result matches our intuition: the higher the resistance of individual n for the product, the lower the innovation coefficient; the higher n's marginal utility from the social signal, the higher the imitation coefficient.

This simple argument shows that not only complex contagion models, but also simple contagion ones can be reinterpreted in terms of the proposed micro-level framework. Under standard assumption in discrete-choice theory, if the utility from adopting grows as a power-law of the social signal, the two considered complex and simple contagion models emerge naturally in the high- and low-clarity regimes. Taken together, these findings suggest that individual-level models might benefit policies to maximize or prevent social spreading for a broader class of models than the complex contagion one studied in the main text. We leave the derivation of alternative models of simple contagions (e.g., rumor spreading models [44] and empirical diffusion models [45]) for future research.

[1] Vithala R Rao. *Applied conjoint analysis*. Springer Science & Business Media, 2014.

[2] Daniel McFadden. Conditional logit analysis of qualitative choice behavior. In Paul Zarembka, editor, *Frontiers in Econometrics*, pages 105–142. Academic press, New York, 1974.

[3] Kenneth E Train. *Discrete choice methods with simulation*. Cambridge University Press, 2009.

[4] Greg M Allenby and Peter E Rossi. Hierarchical bayes models. *The handbook of marketing research: Uses, misuses, and future advances*, pages 418–440, 2006.

[5] Klaus M Miller, Reto Hofstetter, Harley Krohmer, and Z John Zhang. How should consumers' willingness to pay be measured? an empirical comparison of state-of-the-art approaches. *Journal of Marketing Research*, 48(1):172–184, 2011.

[6] Silvia Pianta, Adrian Rinscheid, and Elke U Weber. Carbon capture and storage in the united states: perceptions, preferences, and lessons for policy. *Energy Policy*, 151:112149, 2021.

[7] Cbc questionnaires and design strategy, . URL https://legacy.sawtoothsoftware.com/help/lighthouse-studio/manual/hid_web_cbc_designs_1.html. Accessed on May 16th, 2024.

[8] Ryan Sermas. *ChoiceModelR: Choice Modeling in R*, 2022. URL https://CRAN.R-project.org/package=ChoiceModelR. R package version 1.3.0.

[9] The basics of interpreting conjoint utilities, . URL https://sawtoothsoftware.com/the-basics-of-interpreting-conjoint-utilities. Accessed on April 19th, 2024.

[10] Douglas Guilbeault and Damon Centola. Topological measures for identifying and predicting the spread of complex contagions. *Nature Communications*, 12(1):1–9, 2021.

[11] Albert-László Barabási. *Network science*. Cambridge University Press, 2016.

[12] Maren Hein, Peter Kurz, and Winfried J Steiner. Analyzing the capabilities of the hb logit model for choice-based conjoint analysis: a simulation study. *Journal of Business Economics*, 90(1):1–36, 2020.

[13] Peter E Rossi, Greg M Allenby, and Sanjog Misra. *Bayesian Statistics and Marketing*. John Wiley & Sons, 2024.

[14] Maren Hein, Peter Kurz, and Winfried J Steiner. On the effect of hb covariance matrix prior settings: A simulation study. *Journal of choice modelling*, 31:51–72, 2019.

[15] Mark Newman. *Networks*. Oxford University Press, 2018.

[16] Jacob Goldenberg, Sangman Han, Donald R Lehmann, and Jae Weon Hong. The role of hubs in the adoption process. *Journal of Marketing*, 73(2):1–13, 2009.

[17] Oliver Hinz, Bernd Skiera, Christian Barrot, and Jan U Becker. Seeding strategies for viral marketing: An empirical comparison. *Journal of Marketing*, 75(6):55–71, 2011.

[18] Barak Libai, Eitan Muller, and Renana Peres. Decomposing the value of word-of-mouth seeding programs: Acceleration versus expansion. *Journal of Marketing Research*, 50(2):161–176, 2013.

[19] Andrea Galeotti and Sanjeev Goyal. Influencing the influencers: a theory of strategic diffusion. *The RAND Journal of Economics*, 40 (3):509–532, 2009.

[20] Duncan J Watts and Peter Sheridan Dodds. Influentials, networks, and public opinion formation. *Journal of Consumer Research*, 34 (4):441–458, 2007.

[21] Pablo Barberá, Ning Wang, Richard Bonneau, John T Jost, Jonathan Nagler, Joshua Tucker, and Sandra González-Bailón. The critical periphery in the growth of social protests. *PLOS ONE*, 10(11):e0143611, 2015.

[22] Fang Zhou, Linyuan Lü, Jianguo Liu, and Manuel Sebastian Mariani. Beyond network centrality: Individual-level behavioral traits for predicting information superspreaders in social media. *National Science Review*, page nwae073, 2024.

[23] Gábor Csárdi, Tamás Nepusz, Vincent Traag, Szabolcs Horvát, Fabio Zanini, Daniel Noom, and Kirill Müller. *igraph: Network Analysis and Visualization in R*, 2024. URL https://CRAN.R-project.org/package=igraph. R package version 2.0.1.1.

[24] Hema Yoganarasimhan. Impact of social network structure on content propagation: A study using youtube data. *Quantitative Marketing and Economics*, 10(1):111–150, 2012.

[25] Gabriel Rossman and Jacob C Fisher. Network hubs cease to be influential in the presence of low levels of advertising. *Proceedings of the National Academy of Sciences*, 118(7):e2013391118, 2021.

[26] Alex Bavelas. Communication patterns in task-oriented groups. *The Journal of the Acoustical Society of America*, 22(6):725–730, 1950.

[27] Fang Zhou, Linyuan Lü, and Manuel Sebastian Mariani. Fast influencers in complex networks. *Communications in Nonlinear Science and Numerical Simulation*, 74:69–83, 2019.

[28] Flaviano Morone and Hernán A Makse. Influence maximization in complex networks through optimal percolation. *Nature*, 524(7563): 65–68, 2015.

[29] DJ Watts. A simple model of global cascades on random networks. *Proceedings of the National Academy of Sciences of the United States of America*, 99(9):5766–5771, 2002.

[30] Thomas W Valente. Social network thresholds in the diffusion of innovations. *Social networks*, 18(1):69–89, 1996.

[31] Damon Centola and Michael Macy. Complex contagions and the weakness of long ties. *American journal of Sociology*, 113(3):702–734, 2007.

[32] Douglas Guilbeault, Joshua Becker, and Damon Centola. Complex contagions: A decade in review. *Complex Spreading Phenomena in Social Systems*, pages 3–25, 2018.

[33] Frank M Bass. A new product growth for model consumer durables. *Management science*, 15(5):215–227, 1969.

[34] Robert J MacCoun. The burden of social proof: Shared thresholds and social influence. *Psychological Review*, 119(2):345, 2012.

[35] Jean-Philippe Bouchaud. Crises and collective socio-economic phenomena: simple models and challenges. *Journal of Statistical Physics*, 151:567–606, 2013.

[36] Dean Eckles, Elchanan Mossel, M Amin Rahimian, and Subhabrata Sen. Long ties accelerate noisy threshold-based contagions. *Nature Human Behaviour*, pages 1–8, 2024.

[37] Matteo Marsili. On the multinomial logit model. *Physica A: Statistical Mechanics and its Applications*, 269(1):9–15, 1999.

[38] Graeme Blair, Rebecca Littman, and Elizabeth Levy Paluck. Motivating the adoption of new community-minded behaviors: An empirical test in nigeria. *Science Advances*, 5(3):eaau5175, 2019.

[39] Sonja Vogt, Nadia Ahmed Mohmmed Zaid, Hilal El Fadil Ahmed, Ernst Fehr, and Charles Efferson. Changing cultural attitudes towards female genital cutting. *Nature*, 538(7626):506–509, 2016.

[40] Charles Efferson, Sonja Vogt, and Ernst Fehr. The promise and the peril of using social influence to reverse harmful traditions. *Nature Human Behaviour*, 4(1):55–68, 2020.

[41] Sara M Constantino, Gregg Sparkman, Gordon T Kraft-Todd, Cristina Bicchieri, Damon Centola, Bettina Shell-Duncan, Sonja Vogt, and Elke U Weber. Scaling up change: A critical review and practical guide to harnessing social norms for climate action. *Psychological Science in the Public Interest*, 23(2):50–97, 2022.

[42] Andreas Lanz, Jacob Goldenberg, Daniel Shapira, and Florian Stahl. Climb or jump: Status-based seeding in user-generated content networks. *Journal of Marketing Research*, 56(3):361–378, 2019.

[43] Fine F Leung, Flora F Gu, Yiwei Li, Jonathan Z Zhang, and Robert W Palmatier. Influencer marketing effectiveness. *Journal of Marketing*, 86(6):93–115, 2022.

[44] Javier Borge-Holthoefer and Yamir Moreno. Absence of influential spreaders in rumor dynamics. *Physical Review E*, 85(2):026116, 2012.

[45] Sinan Aral and Paramveer S Dhillon. Social influence maximization under empirical influence models. *Nature Human Behaviour*, 2(6): 375–382, 2018.
