# Supplementary material for: Integrating behavioural experimental findings into dynamical models to inform social change interventions
Source: Nat Hum Behav. 2026 Mar 16;10(5):873–83. doi: 10.1038/s41562-026-02417-4 (PMC13661244; doi:10.1038/s41562-026-02417-4)
Supplement: Supplementary file 3 [file 41562_2026_2417_MOESM3_ESM.pdf]

nature portfolio

Peer Review File

Integrating behavioral experimental findings into dynamical models to inform social change interventions

Corresponding Author: Dr Manuel Sebastian Mariani

Version 0:

Decision Letter:

15th October 2024

Dear Dr Mariani,

Thank you for submitting your manuscript, entitled "Integrating behavioral experimental findings into dynamical models to inform social change interventions".

I'm pleased to say that we are interested in sending your manuscript out to review at Nature Human Behaviour. However, we have noticed that there is no mention of any research ethics (IRB) approval in your manuscript (including in the Supplementary Information). Our policy is that all human behavioural research must have either received approval from an appropriate review committee, or have been declared exempt from review by such a committee.

We ask that you resubmit your manuscript including details of the IRB approval or exemption (this should be in the main text Methods). Please do not hesitate to contact me if you have any questions or would like to discuss this further.

Please use the link below to submit your revised manuscript and related files:

Link Redacted

Note: This URL links to your confidential home page and associated information about manuscripts you may have submitted, or that you are reviewing for us. If you wish to forward this email to co-authors, please delete the link to your homepage.

We look forward to seeing the revised manuscript and thank you for the opportunity to review your work.

Sincerely,
[redacted]

[redacted]
Nature Human Behaviour

Version 1:

Decision Letter:

8th January 2025

Dear Dr Mariani,

Thank you once again for your manuscript, entitled "Integrating behavioral experimental findings into dynamical models to inform social change interventions", and for your patience during the peer review process.

Your Article has now been evaluated by 3 referees. You will see from their comments copied below that, although they find your work of considerable potential interest, they have raised quite substantial concerns. In light of these comments, we cannot accept the manuscript for publication, but would be interested in considering a revised version if you are willing and

able to fully address reviewer and editorial concerns.

We hope you will find the referees' comments useful as you decide how to proceed. If you wish to submit a substantially revised manuscript, please bear in mind that we will be reluctant to approach the referees again in the absence of major revisions. We are committed to providing a fair and constructive peer-review process. Do not hesitate to contact us if there are specific requests from the reviewers that you believe are technically impossible or unlikely to yield a meaningful outcome.

In particular, it will be important to address Reviewer #2's concerns by establishing the validity of the method for inference of individual thresholds. Also, the theoretical background of the current work must be better discussed, as outlined by Reviewer #1 and #3.

Finally, your revised manuscript must comply fully with our editorial policies and formatting requirements. Failure to do so will result in your manuscript being returned to you, which will delay its consideration. To assist you in this process, I have attached a checklist that lists all of our requirements. If you have any questions about any of our policies or formatting, please don't hesitate to contact me.

If you wish to submit a suitably revised manuscript, we would hope to receive it within 4 months. I would be grateful if you could contact us as soon as possible if you foresee difficulties with meeting this target resubmission date.

With your revision, please:

• Include a "Response to the editors and reviewers" document detailing, point-by-point, how you addressed each editor and referee comment. If no action was taken to address a point, you must provide a compelling argument. When formatting this document, please respond to each reviewer comment individually, including the full text of the reviewer comment verbatim followed by your response to the individual point. This response will be used by the editors to evaluate your revision and sent back to the reviewers along with the revised manuscript.

• Highlight all changes made to your manuscript or provide us with a version that tracks changes.

Please use the link below to submit your revised manuscript and related files:

Link Redacted

Note: This URL links to your confidential home page and associated information about manuscripts you may have submitted, or that you are reviewing for us. If you wish to forward this email to co-authors, please delete the link to your homepage.

Thank you for the opportunity to review your work. Please do not hesitate to contact me if you have any questions or would like to discuss the required revisions further.

Sincerely,
[redacted]

[redacted]
Nature Human Behaviour

REVIEWER COMMENTS:

Reviewer #1 (Remarks to the Author):

This MS presents a methodology for estimating network thresholds without the need to collect network data. This is an ambitious and worthwhile endeavor. The authors do so by running fixed choice experiments common in consumer product adoption research. The authors do so by basically postulating the resistance to adoption (threshold level) is the difference from the utility they would derive by adopting based solely the perceived attributes of the innovation (in diffusion terms that is). The overall results suggest that threshold distributions vary by product and that influence maximization can be achieved by considering threshold distributions in the selection of change agents.

A few comments that will hopefully improve the MS:

1) complex contagion vs threshold models. The authors need not invoke "complex" contagion as it is unclear whether there exist simple contagions. I've never witnessed one and the epidemiologists I know do not believe there is on. It seems the focus is primarily on estimating the threshold distribution and the literature on complex contagions always assumes a uniform threshold which seems unlikely.

2) it seems the main thrust of the paper is to find a way to estimate population-level or at least community-level thresholds

absent the ability to collect network data. I think the authors should state this clearly.

3) Page 1: “Complex contagion models link individual-level and collective dynamics by assuming that choices are determined by an individual-level behavioral trait – her threshold level. Yet surprisingly, there is little evidence on the empirical individual-level threshold levels [20, 42], and a scarcity of studies attempting to link empirical thresholds with the collective dynamics of new product and behavior adoption processes.”

It would help to cite the empirical work on network thresholds starting with Valente’s 1996 “Social network thresholds in the diffusion of innovations, Social Networks, vol. 18 which is the origin of this idea; or see Valente 1995 Network Models of the Diffusion of Innovations. Valente & Saba on how thresholds enable estimation of the two-step flow model, etc.

4) Page 3: “For individuals with $\hat{R}_{ni} < 0$, Eq. 1 delivers $\hat{\tau}_{ni} < 0$; these individuals correspond to the innovators in Rogers’ innovation diffusion theory [50]. “

Just to set the record straight, it is not Rogers’ theory but was developed before he entered the field. Lionberger’s 1960 “Adoption of New Ideas & Practices”; Coleman Katz & Menzel study of physicians, etc.

Also note, non-innovators can have zero thresholds as has been demonstrated in the work cited above.

5) Page 4: “For both experiments, we collect choice data from Prolific participants … “
What are Prolific participants?

6) The authors discuss seeding strategies but seem to ignore much of the literature on this topic as well such as Valente’s 2012 Network Interventions, Science; Borgatti’s 2006 Key Players; Ott et al., 2020 “Strategic Players for Identifying Optimal Social Network Intervention Subjects, Social Networks; Robins et al., 2023, Multi-level Network Interventions: Goals, Actions, and Outcomes, Social Networks.
Also see Valente & Vega Yon, 2020, Diffusion/Contagion Processes on Social Networks, Health Education & Behavior which documents the importance of the threshold distribution for diffusion rates and prevalence.

7) I do think the authors should mention that in a perfect world they would be able estimate the threshold distribution through the proposed methodology and compare to a real-world network diffusion process.

Reviewer #2 (Remarks to the Author):

Apologies for the delayed review.

My comments will be limited primarily to the choice modeling component as it is my understanding that this is why I've been selected as a reviewer. I have no comments on the computational modeling component of the paper as it is outside my area of expertise and will defer to other reviewers who may be better equipped to evaluate the contribution.

The paper aims to connect models of "social contagion" in behavior - where individuals make choices not only based on their own personal utility from consuming/choosing an alternative but also based on their beliefs about how many other individuals in their social neighborhood make the same choice.

The empirical component of this paper involves two conjoint choice experiments - one on attitudes towards carbon capture policy (PS) and another on instant messaging app adoption (AA). The paper approaches these using the standard utility modeling framework commonly used in market research. While I am relatively familiar with these methods, I think it is worth noting (to contextualize my expertise) that the tradition in my particular field is to treat these experiments not as preference elicitation tasks but rather as factorial experiments where the target parameter of interest is some sort of "average treatment effect". This is not to say that the paper's approach is inappropriate - in fact, it is arguably more clearly legible with respect to the target audience and I think generates the correct quantities of interest.

My main concern is the extent to which it is possible to reliably recover individual-level parameters in any conjoint setting and whether the results are sensitive to particular modelling choices inherent in the Hierarchical Bayes' approach. The paper relies pretty heavily on the ability to obtain individual-level utility parameters in order to calculate the implied "threshold" at which social influence induces choice behavior for each unit in the study. Each study has roughly 300 participants and each participant evaluates only 15 choice tasks. Since it is impossible to recover individual-level partworths from only 15 choice tasks (as there are 3,840 unique possible profiles in the PS study), conjoint estimation relies on some degree of partial pooling across individuals when estimating partworth coefficients by way of the hierarchical bayesian model (and, by extension, the additivity assumption in the utility function). This makes me somewhat skeptical of then attempting to recover and interpret individual-specific estimates as these are inherently driven by the degree of partial pooling induced by the prior specification in the hierarchical model. Would a more diffuse prior induce a different distribution of "thresholds"? Obviously a very strong prior that induced no pooling would imply no variation in thresholds for units in the sample.

In practice that I'm familiar with for hierarchical models such as this one, the interest is usually in allowing the hierarchical structure to model some sort of intercept shift while the main theoretical interest is in some sort of "pooled" parameter like a slope coefficient (here, a partworth). Otherwise, the goal is pure prediction of some outcome rather than interpretation of the

underlying parameter estimates. Very rarely, in my experience, does one attempt to interpret the *individual-level* estimates given that we know that they are biased (which is the entire point of the hierarchical model) and heavily dependent on the imposed parametric structure (one sees this in Figure E in particular).

I would also find it very useful to see estimates of the 5 different "social endorsement" levels treated as categorical variables rather than assuming a linear functional form a-priori, at least to validate the plausibility of this linearity assumption in the overall approach.

Aside from these points, I will defer to the other reviewers as this paper is very much far outside of my field and expertise otherwise.

Reviewer #3 (Remarks to the Author):

The manuscript addresses an important and timely topic in behavioral and computational social science, focusing on the feasibility of integration of individual preferences into collective behavior models. The work is particularly valuable for its empirical approach, as it tests the feasibility of deriving threshold distributions empirically. It does so in two distinct domains: one with high social influence and one with lower social influence, revealing its generality. The study goes further by generating new theoretical hypothesis for effective interventions on these studied systems. It contributes significantly to the literature on social tipping points and behavior dynamics, especially in the integration of theoretical and empirical methods. In general, both the text and the supplementary material are thorough and well-structured, enhancing transparency and reproducibility.
However, the authors' claim to have developed a novel framework mapping individual preferences to collective behaviors through utility functions is overstated. While their empirical application is innovative and significant, the theoretical groundwork for such frameworks already exists in prior work. The authors should clarify this distinction and appropriately cite the relevant foundational literature.
I recommend a revision of the manuscript for better contextualization and clarity on the contribution as well as clarity on the role of some of the sections. Some more detailed comments below.

The manuscript is methodologically sound.
As I mentioned, my most prominent concern is that the authors claim developing the framework that maps individual preferences to collective behavior through building these utility functions that result in complex contagion thresholds and that is just not correct. The authors do merit the empirical composition of these utilities to be applied in a (complex) contagion setting, testing of feasibility of deriving these threshold empirically, and they show it is generalizable by doing it across two different topics, specifically one with high social influence and one with lower. What they have already merits publication in the journal, even without the novelty of the framework.
The theoretical works by Sergey Gavrilets and others more recently already do this very closely. In "The dynamics of injunctive social norms", from 2020, there are already formulations of behavioral change based on utilities derived from different norms. In Gavrilets' review "Modelling social norms: an integration of the norm-utility approach with beliefs dynamics" (2024) they describe combinations of norm-utility approaches with beliefs dynamics in modeling the emergence, persistence and evolution of social norms. Even closer to the framework here presented is the work by Yang et al. "Sociocultural determinants of global mask-wearing behavior" (2022) that uses a game-theoretic model derive threshold distributions and explore the socio-cultural factors influencing mask-wearing during the COVID-19 pandemic. Similarly, the new work by Mittal, Constantino, and Vasconcelos, "Anti-conformists catalyze societal transitions and facilitate the expression of evolving preferences" (2024) derived multiple threshold responses in reversible systems, including for conformity (one threshold in one direction), anti-conformity (one threshold in the opposite direction), and Multithreshold responses. Some of these works even have the expression of the threshold as a function of the game theoretic utility parameters, just like presented here. An interesting aspect is that the authors find signs of a very small minority of potential anti-conformists (negative thresholds), which is a very nice connection and has never been tested.

The paper represents an evidence-based advance, primarily due to its empirical application of utility functions in a complex contagion setting. The rigorous testing of thresholds across two domains provides valuable insights into the role of social influence in behavioral dynamics. Although the theoretical novelty is limited, the methodological and applied aspects of the work ensure its significance for the field. The results will likely be of immediate interest to scholars in behavioral science, computational modeling, and interdisciplinary studies on social tipping points.

The methodology is robust, and the data are well-presented. The reporting of data and methodology is sufficiently detailed to enable reproducibility. The authors effectively use conjoint experiments to derive utility functions and thresholds, but a minor point regards the limitations regarding the reduced social aspects that were tested in the experiments, which should be explicitly acknowledged. For instance, the reliance on "percent friends" as a measure of social influence leaves questions about whether responses are a function of percentage or absolute number; other aspects like the size of individuals' networks, and their roles within their communities could also be tested in the future to bring together the theoretical hypothesis that exist in the literature.

Other points:
Explicit expressions for the total costs given the product utilities in the different scenarios would be useful.
In figure 2, are the bars very small? How many simulations are done and do any share initial conditions (e.g., on who is

where in the network)? How does it vary across those identical systems (i.e., by fixing the seed or initial distribution of agents and running it with all the interventions, as opposed to resampling it?)
Overall, I would recommend trimming down section S6 to focus on the processes actually studied. For instance, the connection to the Bass model is a weak derivation since at first relies on weak selection---or what the authors call low clarity---to have a constant probability of imitation/innovation of ½ and then to have a probability different from ½ requires reintroducing the frequency dependence, which does not necessarily affect p_n. The Bass model is one where we mostly have a regime where innovation is zero or close to zero and the dominant term is imitation. This occurs mostly when individuals' thresholds are 1 and interactions are pairwise. In any case, I think this is beyond the point of the paper and really just a detour that confuses more than it adds.
The preferential attachment section is also a strange detour that I didn't fully understand what it was trying to convey, since splitting the utility into product properties and social influence is the point of the experiments. A major rewrite of this section is required to connect it to the main text and a precise indication in the main text what to look for in this section that is relevant to the paper is also needed.

Version 2:

Decision Letter:

31st July 2025

Dear Dr Mariani,

Thank you once again for your revised manuscript, entitled "Integrating behavioral experimental findings into dynamical models to inform social change interventions," and for your patience during the re-review process.

Your manuscript has now been evaluated by the original three referees. You will see that Referee #1 and #3 are happy to recommend publication, but Referee #2 still has some important outstanding concerns, and requests additional robustness/sensitivity checks. We remain interested in the possibility of publishing your study in Nature Human Behaviour, but would like to consider your response to these outstanding concerns in the form of a revised manuscript before we make a decision on publication.

In sum, we invite you to revise your manuscript taking into account all reviewer and editor comments. We are committed to providing a fair and constructive peer-review process. Do not hesitate to contact us if there are specific requests from the reviewers that you believe are technically impossible or unlikely to yield a meaningful outcome.

We hope to receive your revised manuscript within 4-8 weeks. I would be grateful if you could contact us as soon as possible if you foresee difficulties with meeting this target resubmission date.

With your revision, please:

• Include a "Response to the editors and reviewers" document detailing, point-by-point, how you addressed each editor and referee comment. If no action was taken to address a point, you must provide a compelling argument. This response will be used by the editors and reviewers to evaluate your revision.

• Highlight all changes made to your manuscript or provide us with a version that tracks changes.

Please use the link below to submit your revised manuscript and related files:

Link Redacted

Note: This URL links to your confidential home page and associated information about manuscripts you may have submitted, or that you are reviewing for us. If you wish to forward this email to co-authors, please delete the link to your homepage.

We look forward to seeing the revised manuscript and thank you for the opportunity to review your work. Please do not hesitate to contact me if you have any questions or would like to discuss these revisions further.

Sincerely,
[redacted]

[redacted]
Nature Human Behaviour

REVIEWER COMMENTS:

Reviewer #1 (Remarks to the Author):

The authors have diligently responded to the prior round of reviews, and I have no further comments.

Reviewer #2 (Remarks to the Author):

First, apologies to the editors and reviewers for the delayed review.

I appreciate the extensive efforts from the authors to revise the draft in light of my somewhat outside-of-the-discipline concerns. Overall, I'm happy with the edits and they have largely satisfied my original concerns. In particular, I appreciate the explicit mention that the individual-level thresholds are identified by way of the parametric assumptions of the model - namely that our prior belief is that the distribution of individual level has a particular parametric form. While I understand that the marketing literature considers these assumptions relatively unproblematic, I don't think this is as widely shared - especially in my corner of the social science world, so it's good that the paper is clear about the kind of intellectual "gamble" being made.

I think the exercise in S3 is helpful for characterizing sensitivity to the modeling assumptions and I'm glad to see it. I'm unsurprised that the uninformative prior generates significant problems -- some regularization is essential for the kind of task being undertaken here. And it's good to see little variation as the strength of the prior towards homogeneity changes. My only concern is in the way that the ground truth is being generated. It's a little unclear from the explanation what it means that the "ground-truth" utilities were generated from the estimates in the model? What - if any - heterogeneity is there in the unit-level part-worths? If these are the ones obtained from the individual-level HB estimates, then I worry that the simulation study is a bit too "easy" -- the heterogeneity will follow the nice normal structure imposed by the simulation. What I think would help push the prior sensitivity a bit more is to consider more "problematic" distributions -- particularly multi-modal ones. Maybe consider a simulation with two latent groups - those for whom the partwise coefficient is positive and another for whom its negative? I would like to know how much the *normality* in the prior structure bites in addition to the prior variance (where, with sufficient data, the likelihood should overcome all but the strongest prior spec).

Otherwise, the justification of the linearity assumption for the adoption attribute seems feasible - at a minimum, the linear approximation doesn't appear to lose all that much (Except maybe at the top end where you see minimal difference between 76 and 98). I'm not too troubled by the interpolation here.

So overall, I'd push towards a conditional accept with a bit more clarity on the nature of *exactly* how the individual partworths were generated from the recovered applied parameters (my understanding is that the study is just using the estimated \beta_n, so there is heterogeneity, but of a very particular type) and maybe an additional variation on the simulation that breaks the single-mode structure of the individual partworths (assuming the above is correct re: how they're estimated). If it does seem to perform poorly, I don't think this is a *problem* for the final results - just a useful caveat on how we should think about the value of adoption. Something I didn't raise in the original review (so I don't think it's *critical* here) is to possibly consider interactions between the partworths as well? How much does the additivity and separability in the utility model "bite" as an assumption? Given my experience with the marketing literature, this tends to be an important assumption (otherwise, the parameter space gets unwieldy quite fast), but I do wonder how salient it is. Possibly a sentence or two remarking on this alongside the other model-sensitivity remarks would help.

Reviewer #3 (Remarks to the Author):

I thank the authors for taking the time to read the additional literature and for thoroughly addressing not only my points but also those of the other reviewers. The additional sensitivity analysis makes the results even more convincing.
The paper is now well contextualized and provides an accurate account of its contribution, showing how it clearly moves the field forward.
I still think the connection to Bass diffusion is a bit of an extra bit connecting complex to simple contagion. This is, however, more of a personal taste in paper style and not a judgment of the quality. If you really want to keep the bit, then I would fully explain that, when \epsilon_{ni} is iid and Gumbel(0, sigma), Pi_ni=exp(Uni*/sigma)/Sum(exp(Unj*/sigma), i.e., the exponentials are divided by sigma (which means that your clarity parameter c=1/sigma). I think that is cleaner than the abuse of notation "It is useful to consider a clarity parameter [34] and, with a slight abuse of notation, rewrite the utility from alternative i in the form". Alternatively, maybe even simpler, just define U_ni = U_ni* + 1/c epsilon_ni, where epsilon is the Gumber(0,1) you define and the higher c, the clarity, the lower the noise. And everything follows without abuse of notation.

I am in strong support of publication.

Reviewer #3 (Remarks on code availability):

The code and data are not yet available, but the authors say they will make them available upon publication.

Version 3:

Decision Letter:

Our ref: NATHUMBEHAV-24103958C

5th November 2025

Dear Dr. Mariani,

Thank you for submitting your revised manuscript "Integrating behavioral experimental findings into dynamical models to inform social change interventions" (NATHUMBEHAV-24103958C).

Having considered the changes you've made, we will be happy in principle to publish this manuscript in Nature Human Behaviour, pending minor revisions to comply with our editorial and formatting guidelines.

We are now performing detailed checks on your paper and will send you a checklist detailing our editorial and formatting requirements within two weeks. Please do not upload the final materials and make any revisions until you receive this additional information from us.

Please do not hesitate to contact me if you have any questions.

Sincerely,
[redacted]

[redacted]
Nature Human Behaviour

Open Access This Peer Review File is licensed under a Creative Commons Attribution 4.0 International License, which permits use, sharing, adaptation, distribution and reproduction in any medium or format, as long as you give appropriate credit to the original author(s) and the source, provide a link to the Creative Commons license, and indicate if changes were

made.
In cases where reviewers are anonymous, credit should be given to 'Anonymous Referee' and the source.
The images or other third party material in this Peer Review File are included in the article's Creative Commons license, unless indicated otherwise in a credit line to the material. If material is not included in the article's Creative Commons license and your intended use is not permitted by statutory regulation or exceeds the permitted use, you will need to obtain permission directly from the copyright holder.
To view a copy of this license, visit https://creativecommons.org/licenses/by/4.0/

“Integrating behavioral experimental findings into dynamical models to inform social change interventions”: Reply Letter

Dear Editor,

We are very pleased to submit the revised version of the article “Integrating behavioral experimental findings into dynamical models to inform social change interventions" to *Nature Human Behaviour*. We would like to thank you and the three Reviewers for all the suggestions, which helped us to substantially improve the positioning of the paper in the literature and to demonstrate the robustness of our findings with respect to choice model specification.

We are grateful that overall, the contribution of our work has been appreciated by the Reviewers. In particular, they highlighted the following main contributions:

- Novel methodology for estimating individual-level network thresholds without collecting social-network data, and systematic validation of those thresholds across two distinct empirical domains.
- Empirical linkage between individual-level utility models and collective-level contagion dynamics.
- Using the estimated thresholds to generate new theoretical hypotheses for effective social-network interventions, guiding the selection of change agents to maximize diffusion under realistic cost scenarios.

In the rest of this document, we address all the points the Reviewers raised, which has resulted in a major revision of the manuscript. Before addressing each point in detail, we wish to provide you with a summary of the main changes:

- We have extended the literature by integrating early works on social network thresholds as well as key papers on seeding strategies suggested by Reviewer 1.
- As required by Reviewer 2, we have demonstrated the robustness of our estimation results at both the individual and collective levels with respect to choices of the prior distributions in the Bayesian estimation.
- Following Reviewer 3’s advice, we have clarified that our main contribution to the threshold estimation is mostly empirical and not theoretical. In particular, we covered more extensively the literature on utility-based social norm models, and emphasized how our work advances the literature by demonstrating the feasibility of threshold estimation and its implications for seeding policies.
- Following Reviewer 3’s advice, we have ensured that Supplementary Note S6 focuses on social contagion processes, and removed the part on preferential attachment models.
- To comply with the Revision checklist for Nature Human Behaviour manuscripts, we have shortened the “Implications for seeding policies" sections to comply with 5000-word limit, added Supplementary Figures S3, S7, S22, S23, and provided details on the test statistics beyond the p-values.

We hope that the changes we made satisfactorily address the Reviewers’ concerns, and we are very much looking forward to your feedback.

Best regards,
The authors

Response to Reviewer 1

> *This MS presents a methodology for estimating network thresholds without the need to collect network data. This is an ambitious and worthwhile endeavor. The authors do so by running fixed choice experiments common in consumer product adoption research. The authors do so by basically postulating the resistance to adoption (threshold level) is the difference from the utility they would derive by adopting based solely the perceived attributes of the innovation (in diffusion terms that is). The overall results suggest that threshold distributions vary by product and that influence maximization can be achieved by considering threshold distributions in the selection of change agents.*

We are thankful for the Reviewer's concise and accurate summary of our review, their recognition of the ambition and worthiness of our work, and all their critical comments. We have performed a major revision to modify the article based on the Reviewer's comments, and substantially improved the article as a result.

> *1) complex contagion vs threshold models. The authors need not invoke "complex" contagion" as it is unclear whether there exist simple contagions. I've never witnessed one and the epidemiologists I know do not believe there is on. It seems the focus is primarily on estimating the threshold distribution and the literature on complex contagions always assumes a uniform threshold which seems unlikely.*

We appreciate the Reviewer's feedback on the distinction between complex contagion and threshold-based models. We agree with the Reviewer that our focus in this study is primarily on estimating the threshold distribution, whereas the literature on complex contagions often relies on uncalibrated assumptions on the threshold distribution (e.g., uniform distribution). The "complex contagion" terminology comes from the literature that has discussed the differences between simple and complex contagion across diverse disciplines (e.g., [10–12, 25, 35, 42]). At the same time, we believe that the distinction between simple and complex contagions (and to which extent simple contagions exist) is not central to our main contribution. For this reason, in the spirit of the Reviewer's advice, we avoided emphasizing the distinction between simple vs. complex contagions. As a consequence, in the revised manuscript, we refer to the diffusion model as the threshold-based model, whereas we still acknowledge the literature on complex contagion as a theoretical background for our work.

> *2) it seems the main thrust of the paper is to find a way to estimate population-level or at least community-level thresholds absent the ability to collect network data. I think the authors should state this clearly.*

We are thankful for the Reviewer's advice. We emphasized this point in the introduction:

"We develop here a method to estimate individual-level thresholds without requiring historical adoption nor social network data, we validate it across two distinct empirical domains, and we demonstrate its usefulness to inform network interventions for social change. Specifically, . . . "

> *3) Page 1: "Complex contagion models link individual-level and collective dynamics by assuming that choices are determined by an individual-level behavioral trait – her threshold level. Yet surprisingly, there is little evidence on the empirical individual-level threshold levels [20, 42], and a scarcity of studies attempting to link empirical thresholds with the collective dynamics of new product and behavior adoption processes."*
>
> *It would help to cite the empirical work on network thresholds starting with Valente's 1996 "Social network thresholds in the diffusion of innovations, Social Networks, vol. 18 which is the origin of this idea; or see Valente 1995 Network Models of the Diffusion of Innovations. Valente & Saba on how thresholds enable estimation of the two-step flow model, etc.*

We have incorporated the suggested references, and we are thankful for the Reviewer's advice. These works are indeed very relevant. We integrated them in the Section "From individual-level preferences to thresholds" as follows:

"Pioneering empirical efforts [44, 45, 49] calculated individual thresholds from historical adoption data by relying on simple heuristics such as the percentage of adopters in one's personal network at adoption time. While intuitive and practical, these heuristics are difficult to refute in practice, as observed in ref. [46]. To date, there is a lack of empirically validated methods to estimate individual thresholds and integrate them into social network interventions [21, 22, 37]."

We also mentioned some of these seminal works in the "Implications for seeding policies" section:

"The underlying hypothesis is that as low-threshold individuals are willing to adopt a new idea earlier than their peers, they should be engaged early on to create sufficient early momentum and form a critical mass for the desired behavioral change [45, 47]."

> *4) Page 3: "For individuals with $R_{ni} < 0$, Eq. 1 delivers $\tau_{ni} < 0$; these individuals correspond to the innovators in Rogers' innovation diffusion theory [50]."*
>
> *Just to set the record straight, it is not Rogers' theory but was developed before he entered the field. Lionberger's 1960 "Adoption of New Ideas & Practices"; Coleman Katz & Menzel study of physicians, etc.*
>
> *Also note, non-innovators can have zero thresholds as has been demonstrated in the work cited above.*

We agree with the Reviewer that these studies laid the empirical and conceptual foundations of the innovation diffusion theory. We avoided referring to the innovation diffusion theory as “Rogers’ theory". Prior works discussed indeed the various factors that can prevent zero-threshold individuals from being innovators, and the fundamental distinction between innovators with respect to one’s personal network and the whole social system. We integrated all these considerations in the following paragraph:

“Using the terminology of the innovation diffusion theory [13, 27, 39, 41], these individuals act as innovators with respect to their personal network [45], as they adopt as soon as they become aware of product i regardless of the social signal’s strength; for practical purposes, their estimated threshold can be set to zero. Note that they may not necessarily be innovators with respect to the whole social system, as they may still adopt late if they are not exposed early on to the product [45]."

> *5) Page 4: “For both experiments, we collect choice data from Prolific participants . . . “ What are Prolific participants?*

We have added an explanation to the main text:

“For both experiments, we collect choice data from participants recruited through Prolific, an online platform commonly used for sourcing high-quality respondents for research studies [15]"

> *6) The authors discuss seeding strategies but seem to ignore much of the literature on this topic as well such as Valente’s 2012 Network Interventions, Science; Borgatti’s 2006 Key Players; Ott et al., 2020 “Strategic Players for Identifying Optimal Social Network Intervention Subjects, Social Networks; Robins et al., 2023, Multi-level Network Interventions: Goals, Actions, and Outcomes, Social Networks. Also see Valente & Vega Yon, 2020, Diffusion/Contagion Processes on Social Networks, Health Education & Behavior which documents the importance of the threshold distribution for diffusion rates and prevalence.*

We appreciate the suggestions of the Reviewer regarding the expansion of the seeding literature. In the revised version, we have integrated all the contributions recommended by the Reviewer. The revised paragraph in the Section “Implications for seeding policies" reads:

“We consider here the seeding problem where a social change practitioner [14] (e.g., a government or an organization) seeks to determine which nodes in a social network to incentivize first to promote the large-scale adoption of a new product or behavior (hereafter, product) [4, 48, 50]. This problem represents a specific type of network intervention, which refers to purposeful actions taken by a change practitioner to leverage the structure of social networks in achieving a predefined objective [38, 47]. Structure-based seeding policies are agnostic to a potential target’s behavioral characteristics, and they select a set of seeds according to the nodes’ centrality in the relevant social network [4, 48] or to structural properties of the set [7, 29, 33, 34, 36]. On the other hand, behavior-based policies (i.e., policies that take into account individual behavioral characteristics such as the individual thresholds) assume that some individuals are more amenable to change and, therefore, willing to initiate the adoption process for a new product or behavior [9, 17, 45, 49]. "

Additionally, in the Section “From individual-level preferences to thresholds", we have incorporated Valente & Vega Yon (2020) among the references that demonstrate the importance of threshold distributions in diffusion processes:

“The relevance of the threshold distribution for diffusion outcomes [20, 45, 50, 51] seems to directly call for efforts to measure individual-level thresholds from empirical data to calibrate threshold-based simulations."

> *7) I do think the authors should mention that in a perfect world they would be able estimate the threshold distribution through the proposed methodology and compare to a real-world network diffusion process.*

We added the suggested remark to the Discussion section:

“At the collective level, estimating the threshold distribution through the proposed methodology and comparing the resulting predictions against real-world network diffusion processes is an important direction for future research."

RESPONSE TO REVIEWER 2

> *My comments will be limited primarily to the choice modeling component as it is my understanding that this is why I've been selected as a Reviewer. I have no comments on the computational modeling component of the paper as it is outside my area of expertise and will defer to other Reviewers who may be better equipped to evaluate the contribution.*
>
> *The paper aims to connect models of "social contagion" in behavior - where individuals make choices not only based on their own personal utility from consuming/choosing an alternative but also based on their beliefs about how many other individuals in their social neighborhood make the same choice.*
>
> *The empirical component of this paper involves two conjoint choice experiments - one on attitudes towards carbon capture policy (PS) and another on instant messaging app adoption (AA). The paper approaches these using the standard utility modeling framework commonly used in market research. While I am relatively familiar with these methods, I think it is worth noting (to contextualize my expertise) that the tradition in my particular field is to treat these experiments not as preference elicitation tasks but rather as factorial experiments where the target parameter of interest is some sort of "average treatment effect". This is not to say that the paper's approach is inappropriate - in fact, it is arguably more clearly legible with respect to the target audience and I think generates the correct quantities of interest.*

We thank the Reviewer for the clear and thoughtful summary of our paper's aims and methodology, and for highlighting that our use of conjoint choice experiments as a preference-elicitation framework yields the correct quantities of interest. We appreciate the Reviewer's contextualization of their expertise, which helps us better understand the origin of their comments. In the following, we address their comments both conceptually and through new results.

> *My main concern is the extent to which it is possible to reliably recover individual-level parameters in any conjoint setting and whether the results are sensitive to particular modelling choices inherent in the Hierarchical Bayes' approach. The paper relies pretty heavily on the ability to obtain individual-level utility parameters in order to calculate the implied "threshold" at which social influence induces choice behavior for each unit in the study. Each study has roughly 300 participants and each participant evaluates only 15 choice tasks. Since it is impossible to recover individual-level partworths from only 15 choice tasks (as there are 3,840 unique possible profiles in the PS study), conjoint estimation relies on some degree of partial pooling across individuals when estimating partworth coefficients by way of the hierarchical bayesian model (and, by extension, the additivity assumption in the utility function). This makes me somewhat skeptical of then attempting to recover and interpret individual-specific estimates as these are inherently driven by the degree of partial pooling induced by the prior specification in the hierarchical model. Would a more diffuse prior induce a different distribution of "thresholds"? Obviously a very strong prior that induced no pooling would imply no variation in thresholds for units in the sample.*

The Reviewer raises two primary concerns. First, can we reliably recover and interpret individual-level parameters – such as the adoption threshold – when each respondent provides only 15 choice tasks and the HB model necessarily imposes partial pooling? Second, to what extent do our results depend on the specific HB model setup, especially the parameterization of the priors?

Before presenting our robustness checks, it is important to emphasize that, while the adoption threshold is a well-defined theoretical construct in complex contagion [12], any empirical estimate will inevitably present some degree of dependence on the specifics of the estimation method. Thus, as suggested by the Reviewer, assessing the sensitivity of the threshold estimates to alternative model specifications is both appropriate and necessary to ensure the findings are not an artifact of a particular modeling choice.

We address the Reviewer's concern by: (1) mentioning prior results in the marketing and consumer behavior literature providing evidence for the accuracy of individual estimates produced by HB models; (2) presenting the results of additional robustness checks we performed, following the Reviewer's advice, on individual-level estimates; (3) presenting the results of additional simulations that show the robustness of the derived implications for seeding policies with respect to the HB model specification. We are thankful to the Reviewer for their insightful comment which helped us clarify the impact of model specification on threshold estimation and show the robustness of the results at both the individual and collective level.

1. Prior literature in marketing and consumer behavior

First, we note that modeling individual-level heterogeneity is a central pillar of research in marketing and consumer behavior [1, 40]. Bayesian models in general, and Hierarchical Bayes models in particular, have become key workhorses for this purpose [2]. Consequently, their capabilities and performance in recovering individual-level parameters (especially from few data points at individual level, which is typical in marketing applications) and predicting out of sample have been well documented in the literature – see e.g., [3, 23, 24] for results related to conjoint studies.

These studies typically rely on simulation scenarios where ground-truth values of the partworth utilities are known, and one can measure the difference and/or correlation between the true and estimated partworth utilities [3, 24]. Following this procedure, it has been shown that HB models have a good performance in estimating individual-level parameters from conjoint studies [3, 24]. While the performance is influenced by the specifications of the priors, it generally remains high – except in specific cases, such as when there is a substantial mismatch between the prior variance and the true heterogeneity in the data [23]. This motivated our decision to use HB for the threshold estimation.

2. Robustness at the individual level

Prior specification is an active area of research and providing specific recommendations on optimal priors falls outside the scope of our study. However, the Reviewer's comment motivated us to examine the sensitivity of the estimated adoption threshold to different prior specifications. To this end, following a similar but simplified setup as prior works [3, 23, 24], we performed a simulation study (see Supplementary Note S3 C). In the simulations, the *ground-truth* partworth utilities are the utilities empirically estimated from the two conjoint studies. From these ground-truth utilities, we generated synthetic datasets with 30 and 100 choice tasks per respondent (each task consisting of two alternatives and a status-quo option) together with the corresponding choices, following the procedure in Supplementary Note S3 A. These two datasets complement the original simulated dataset of 15 choice tasks per participant used in the main analysis. The prior specification used in main text follows the default setting in the `ChoiceModelR` [43] package. We additionally tested three HB models that differ from the default model used in the main text in the tightness of the priors, as the tightness (together with the data) determines how much individual estimates are shrinked towards the group mean [23]. Specifically, we test a model with tighter priors and two models with looser priors, obtained by altering the parameters of the inverted Wishart distribution characterizing the covariance of the partworth utility distribution – see Supplementary Note S3 C.

We find that all but the uninformative prior produce similarly accurate partworth estimation, and all methods' mean absolute error decrease as the data size increases (see Figs. 1A,D – presented as Fig. S9 in Supplementary Material). The differences between the models' accuracy is even smaller for the threshold estimation task (see Figs. 1B,E). Importantly, the thresholds estimated through the different models are highly correlated with the ground-truth thresholds (see Figs. 1C,F). For all priors, average Pearson correlations are larger than 0.56 and 0.87 in the PS and AA study, respectively. Taken together, these findings indicate that as long as the choice of priors is sensible, the estimated thresholds are robust with respect to prior specification.

3. Robustness at the collective level

While the results above relate to the robustness of individual-level estimates, we additionally asked whether the results at the collective level are also robust. Using the thresholds estimated under each prior specification, we reran the simulations to identify effective seeding strategies in both cost scenarios (see Supplementary Note S5 D). For all prior specifications, our main results (see Figs. S20 and S21) are in qualitative agreement with those obtained in the main analysis. In the preference-based cost setting, the neighborhood-susceptibility rule consistently outperformed the other strategies regardless of the prior specification. In the centrality-cost scenario, the calibrated complex-contagion centrality remained superior to all alternatives. In other words, even when individual thresholds vary slightly under different priors, our main conclusions on the relative performance of seeding strategies remain unchanged.

Revision

We revised the main text by adding the following considerations to the Methods section:

"Inevitably, the estimation results are to some extent model-dependent, as they reflect the assumptions embedded in the HB model. This dependence diminishes as the number of choice tasks per participant increases, since a broad range of priors will result in similar inferences in large datasets [40]. However, when the number of choice tasks per individual is small relative to the number of parameters to be estimated, the choice of priors might have an impact. Supplementary Notes S3 C–S5 D show that our main results are robust across four model specifications that differ in the assumptions on the prior distributions made in the HB model. With the exception of the extreme case of an uninformative prior, the choice of the prior distribution has a marginal impact on the accuracy of the partworth utility estimates (Fig. 1A,D). Further, except for the uninformative prior, the accuracy of adoption threshold estimates and their correlation with the true thresholds remain high and consistent across

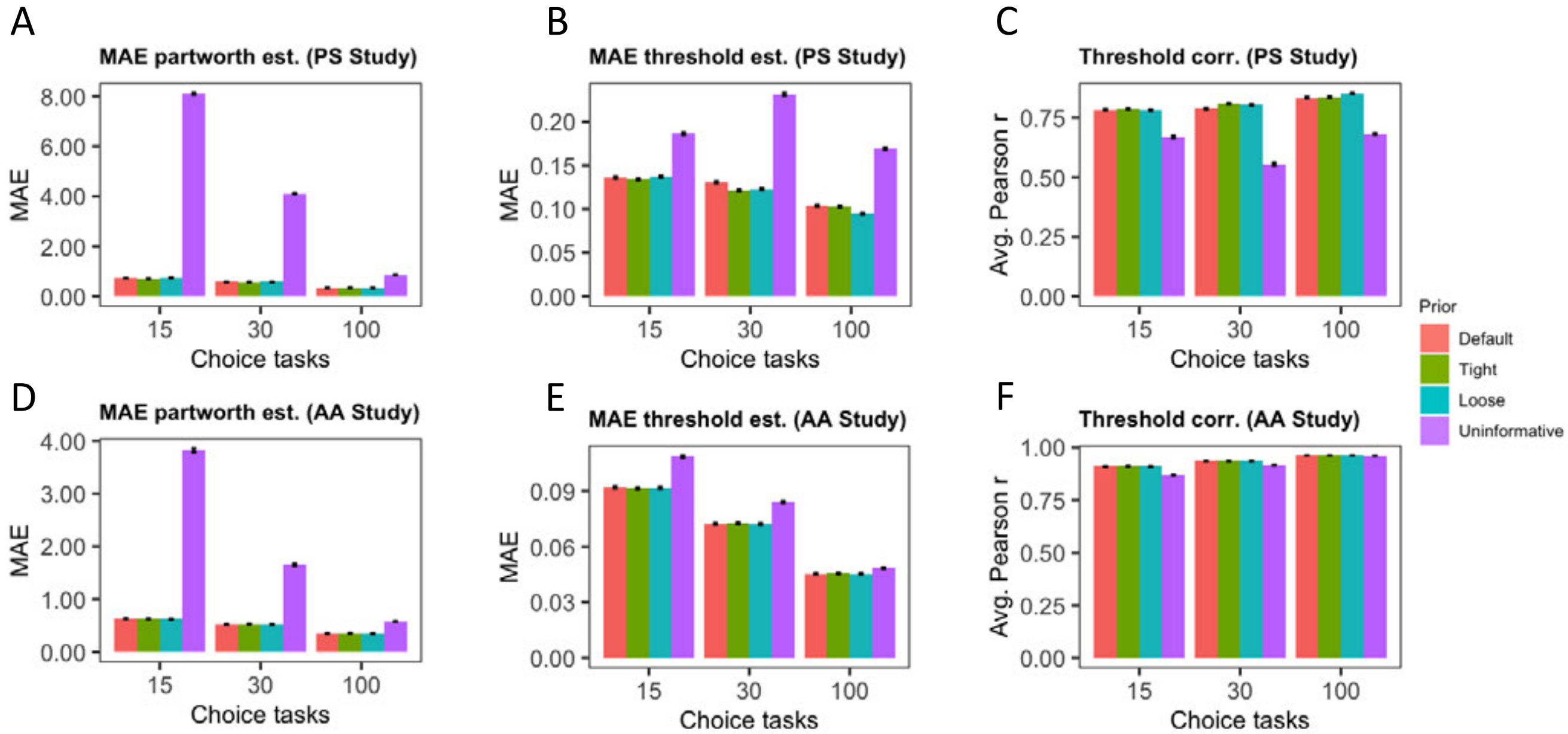

Figure 1: **Estimation accuracy under different prior specification for the PS Study (top row) and the AA Study (bottom row).** All but the very weak prior specification result in similar accuracy of recovering the partworth utilities **(A, D)** and the adoption thresholds **(B, E)** and similar correlations between the true and the estimated thresholds **(C, F)**.

prior specifications (Fig. 1B–C;E–F). As a result, our conclusions on the relative performance of behavior-based seeding strategies is unaffected by prior choice (Figs. S20–S21). Therefore, while the estimated adoption thresholds should not be interpreted as model-free estimates, our sensitivity analysis shows they remain robust across specifications and can reliably inform the design of social-change interventions."

> *In practice that I'm familiar with for hierarchical models such as this one, the interest is usually in allowing the hierarchical structure to model some sort of intercept shift while the main theoretical interest is in some sort of "pooled" parameter like a slope coefficient (here, a partworth). Otherwise, the goal is pure prediction of some outcome rather than interpretation of the underlying parameter estimates. Very rarely, in my experience, does one attempt to interpret the *individual-level* estimates given that we know that they are biased (which is the entire point of the hierarchical model) and heavily dependent on the imposed parametric structure (one sees this in Figure E in particular).*

We thank the Reviewer for clarifying the source of their concern. While individual-level estimates from HB models are indeed subject to shrinkage, they have been shown to be accurate and reliable [3, 24]. As our results suggest (and in line with Ref. [23]), they are also robust to different specification of the priors, as long as priors are sensible. However, we agree that a caveat regarding their interpretation is necessary: these estimates should not be viewed as model-free representations, but rather as reliable approximations that are effective for identifying seeding strategies. This consideration is now reflected in the paragraph we added in the main text (see above). We note our approach aligns with the broader practice in marketing science of using HB models to recover individual-level parameters [1, 40], a practice that has been successfully applied in conjoint studies (for example to estimate consumers' willingness to pay [31]) Our work extends this tradition by using the empirically-measured utilities to derive adoption thresholds that can be used to identify effective seeds for social change interventions.

> *I would also find it very useful to see estimates of the 5 different "social endorsement" levels treated as categorical variables rather than assuming a linear functional form a-priori, at least to validate the plausibility of this linearity assumption in the overall approach.*

We are thankful for the Reviewer's comment, which helps demonstrate the plausibility of the linearity assumption. Following their advice, we have re-estimated the model by treating the five levels as categorical dummies (Section *Linearity of the social signal* in Supplementary Notes S1 B and S1 C. We find a largely monotonic increase in estimated utilities (Supplementary Figs. S4 and S8) and a significant linear correlation between the choice probability and the level of social signal (Pearson's r = 0.77, $p < 0.001$ (PS Study) and Pearson's r = 0.93, $p < 0.001$ (AA Study)). This finding provides empirical support for the plausibility of the linear specification.

> *Aside from these points, I will defer to the other Reviewers as this paper is very much far outside of my field and expertise otherwise.*

We thank the Reviewer again for their thoughtful comments, which have substantially strengthened our results on both individual thresholds and seeding policies by proving the robustness with respect to the prior specification. We look forward to their feedback.

RESPONSE TO REVIEWER 3

> *The manuscript addresses an important and timely topic in behavioral and computational social science, focusing on the feasibility of integration of individual preferences into collective behavior models. The work is particularly valuable for its empirical approach, as it tests the feasibility of deriving threshold distributions empirically. It does so in two distinct domains: one with high social influence and one with lower social influence, revealing its generality. The study goes further by generating new theoretical hypothesis for effective interventions on these studied systems. It contributes significantly to the literature on social tipping points and behavior dynamics, especially in the integration of theoretical and empirical methods.*

We are thankful to the Reviewer for recognizing the importance and timeliness of the topic, and for the accurate summary of our work's contribution.

> *In general, both the text and the supplementary material are thorough and well-structured, enhancing transparency and reproducibility. However, the authors' claim to have developed a novel framework mapping individual preferences to collective behaviors through utility functions is overstated. While their empirical application is innovative and significant, the theoretical groundwork for such frameworks already exists in prior work. The authors should clarify this distinction and appropriately cite the relevant foundational literature. I recommend a revision of the manuscript for better contextualization and clarity on the contribution as well as clarity on the role of some of the sections.*

We are thankful for the Reviewer's acknowledgment of the innovativeness and significance of the empirical applications. As suggested by the Reviewer, we have revised the manuscript to clarify our contribution, and to better contextualize it within the prior works that mapped theoretically individual preferences to collective behaviors. Our revised manuscript acknowledges the theoretical groundwork provided by the papers recommended by the Reviewer, which broadened our coverage of the literature beyond the utility-based models cited in the original submission (e.g., [16, 19]). As detailed in our responses to the subsequent points, we have also expanded the discussion and citations accordingly to ensure proper positioning of our work.

> *The manuscript is methodologically sound. As I mentioned, my most prominent concern is that the authors claim developing the framework that maps individual preferences to collective behavior through building these utility functions that result in complex contagion thresholds and that is just not correct. The authors do merit the empirical composition of these utilities to be applied in a (complex) contagion setting, testing of feasibility of deriving these threshold empirically, and they show it is generalizable by doing it across two different topics, specifically one with high social influence and one with lower. What they have already merits publication in the journal, even without the novelty of the framework.*

We agree with the Reviewer that utility-based decision-making models under social influence have been theoretically examined in prior works [18]. To clarify the positioning of our work, we explicitly state that we "adopted" or "leveraged" a utility-based framework. This ensures clarity in positioning our work, emphasizing that our contribution lies in the empirical application of utility-based model in a complex contagion setting for the estimation of individual thresholds, demonstrating its feasibility and generalizability across domains as well as its implications for social influence maximization.

> *The theoretical works by Sergey Gavrilets and others more recently already do this very closely. In "The dynamics of injunctive social norms", from 2020, there are already formulations of behavioral change based on utilities derived from different norms. In Gavrilets' review "Modelling social norms: an integration of the norm-utility approach with beliefs dynamics" (2024) they describe combinations of norm-utility approaches with beliefs dynamics in modeling the emergence, persistence and evolution of social norms. Even closer to the framework here presented is the work by Yang et al. "Sociocultural determinants of global mask-wearing behavior" (2022) that uses a game-theoretic model derive threshold distributions and explore the socio-cultural factors influencing mask-wearing during the COVID-19 pandemic. Similarly, the new work by Mittal, Constantino, and Vasconcelos, "Anti-conformists catalyze societal transitions and facilitate the expression of evolving preferences" (2024) derived multiple threshold responses in reversible systems, including for conformity (one threshold in one direction), anti-conformity (one threshold in the opposite direction), and Multithreshold responses. Some of these works even have the expression of the threshold as a function of the game theoretic utility parameters, just like presented here. An interesting aspect is that the authors find signs of a very small minority of potential anti-conformists (negative thresholds), which is a very nice connection and has never been tested.*

This is indeed a relevant stream of literature, and we agree with the Reviewer on the importance of citing these works. In light of these works and the above Reviewer's comments, we added the following paragraph to the Section "From individual-level preferences to thresholds":

"The utility-based approach to modeling behavioral change assumes that by adopting a certain new behavior, individuals can gain or lose utility depending on whether the behavior conforms with others' behavior or expectations – see [18] for a review. By translating this assumption into a mathematical decision-making model, assuming

that conforming with others increases one's utility from adopting, one can analytically derive the individual-level threshold to behavioral change as a function of the individual utility parameters in various scenarios [19, 26, 52]. These models can be generalized along multiple dimensions. For example, one can derive downward thresholds (an agent abandons a given alternative when a threshold fraction of her social contacts already adopted it) and even multi-threshold responses by including negative externalities [28] or anticonformist agents [32]. Motivated by this stream of studies, we adopt here a simple utility-based formulation that enables convenient estimation from choice data [19]."

> *The paper represents an evidence-based advance, primarily due to its empirical application of utility functions in a complex contagion setting. The rigorous testing of thresholds across two domains provides valuable insights into the role of social influence in behavioral dynamics. Although the theoretical novelty is limited, the methodological and applied aspects of the work ensure its significance for the field. The results will likely be of immediate interest to scholars in behavioral science, computational modeling, and interdisciplinary studies on social tipping points.*

We are thankful to the Reviewer for recognizing the methodological and empirical contribution of our work as well as its immediate interest to scholars in diverse fields.

> *The methodology is robust, and the data are well-presented. The reporting of data and methodology is sufficiently detailed to enable reproducibility. The authors effectively use conjoint experiments to derive utility functions and thresholds, but a minor point regards the limitations regarding the reduced social aspects that were tested in the experiments, which should be explicitly acknowledged. For instance, the reliance on "percent friends" as a measure of social influence leaves questions about whether responses are a function of percentage or absolute number; other aspects like the size of individuals' networks, and their roles within their communities could also be tested in the future to bring together the theoretical hypothesis that exist in the literature.*

We thank the Reviewer for the positive feedback on our methodology and data presentation. We agree that the reliance on "percentage of friends" could mask effects related to absolute numbers, network size, and individuals' roles. We now explicitly acknowledge these limitations in the revised Discussion section, and suggest these aspects as avenues for future research. The revised paragraph reads:

"Future empirical works could leverage the proposed method for more refined tests of social contagion theories. In our work, all participants were exposed to the percentage of friends who selected each alternative, which necessarily leaves out the variety of social cues encountered in field applications [8]. This suggests two natural directions for generalizing our findings, related to the characteristics of the decision maker and the received social cues, respectively. At the decision-maker level, future studies could examine how the size of an individual's personal network and their individual role within a community affect their thresholds. As for the social cues, future studies could examine whether different types of social cues – e.g., coming from more similar individuals or individuals with different roles in the community – affect individual responses. Addressing these factors in the field might require more sophisticated conjoint designs potentially using vignettes [6]. We envision that these efforts could systematically identify which influence mechanisms and contexts cause social signals to play (or not play) a critical role in shaping individual choices, and what the best policy response could be."

> *Other points: Explicit expressions for the total costs given the product utilities in the different scenarios would be useful.*

Explicit expressions for the total cost in the two scenarios are provided in Methods (Eqs. 5–6).

> *In figure 2, are the bars very small? How many simulations are done and do any share initial conditions (e.g., on who is where in the network)? How does it vary across those identical systems (i.e., by fixing the seed or initial distribution of agents and running it with all the interventions, as opposed to resampling it?)*

As mentioned in the revised Methods section, each simulation run is characterized by a configuration $\mathcal{C}$, which is identified by:

$$\mathcal{C} = \{\text{network, product, survey realization and node assignment}\}.$$

For each configuration, we run all the interventions, which ensures that the interventions' performance can be directly compared to each other and ranked (i.e., it ensures that potential performance differences are not due to differences in the underlying configuration). In the revised Methods section, we specify the number of simulations per seeding policy:

"For a given seeding policy, considering 18 *empirical networks,* 36 *products, and* 5 *realizations of the simulated conjoint survey and random node assignment for the simulated participants (see Supplementary Notes S2–S3 A), we run* $3,240$ *independent simulations."*

The relatively small error bars (standard errors of the mean) reflect that each mean rank is estimated from many runs, which leads to low uncertainty on the mean.

> *Overall, I would recommend trimming down section S6 to focus on the processes actually studied. For instance, the connection to the Bass model is a weak derivation since at first relies on weak selection—or what the authors call low clarity—to have a constant probability of imitation/innovation of* $1/2$ *and then to have a probability different from* $1/2$ *requires reintroducing the frequency dependence, which does not necessarily affect* p_n*. The Bass model is one where we mostly have a regime where innovation is zero or close to zero and the dominant term is imitation. This occurs mostly when individuals' thresholds are 1 and interactions are pairwise. In any case, I think this is beyond the point of the paper and really just a detour that confuses more than it adds.*

We thank the Reviewer for the thoughtful feedback. We agree that the original connection to the Bass model in Section S6 is an approximate derivation, and it is not part of the empirical analysis performed in main text. In response to the Reviewer's comment, we have removed the Section "Alternative spreading theories" from the main text, and now limit the discussion of the Bass model to a brief sentence in the Discussion section, which reads:

"The boundaries to the effectiveness of the utility-based framework adopted here deserve as well to be further investigated in future research. While we assumed a threshold-based diffusion model, aligned with the main paradigm in the social contagion literature [12, 21, 45], we show in Supplementary Note S6 that a utility-based framework under different assumptions that those adopted above still recovers interpretable social spreading models – e.g., the traditional Bass diffusion [5] and logistic diffusion models [30] – which could be investigated in future empirical research."

In the revised sentence, we direct the interested readers to the Supplementary Note S6 for further details. Our aim in retaining this material in the Supplementary Note is to illustrate how the spreading model changes when we relax the assumption of strictly deterministic adoption thresholds and instead allow for stochastic decision-making. This shift leads naturally to a logistic diffusion, as presented in Supplementary Note S6. In this probabilistic setting, we find it interesting to report that the threshold-based and Bass models emerge as approximations of the underlying logistic diffusion model in the high- and low-clarity regimes. We have revised the Supplementary Note to clarify that this is an approximate connection and not a formal derivation.

We believe that this conceptual link still offers value to the reader by helping bridge complex and simple contagion within a unified framework, albeit in an approximate way. However, if the Reviewer feels that its inclusion – even in the Supplementary Material – detracts from the clarity or focus of the paper, we are open to removing it entirely in the final version.

> *The preferential attachment section is also a strange detour that I didn't fully understand what it was trying to convey, since splitting the utility into product properties and social influence is the point of the experiments. A major rewrite of this section is required to connect it to the main text and a precise indication in the main text what to look for in this section that is relevant to the paper is also needed.*

We thank the Reviewer for their careful reading of the Supplementary Note. Our original intention was to offer an interpretation of preferential attachment (PA) within a random utility framework, potentially inspiring future studies on model calibration even in scenarios with high degrees of node preference heterogeneity. However, in line with the Reviewer's comment, we acknowledge that the reinterpretation of PA models within the random utility framework is not directly tied to the empirical focus of the main paper. In particular, we felt that the conceptual link between preferential attachment mechanisms and the network-based diffusion processes we studied in main text is not sufficiently strong to justify its inclusion. To avoid diverting attention from the central contributions of the work, we have decided to remove the discussion of preferential attachment from Supplementary Note S6.

REFERENCES

[1] Allenby, G.M., Rossi, P.E., 1998. Marketing models of consumer heterogeneity. Journal of Econometrics 89, 57–78.

[2] Allenby, G.M., Rossi, P.E., 2006. Hierarchical bayes models. The handbook of marketing research: Uses, misuses, and future advances , 418–440.

[3] Andrews, R.L., Ansari, A., Currim, I.S., 2002. Hierarchical bayes versus finite mixture conjoint analysis models: A comparison of fit, prediction, and partworth recovery. Journal of Marketing Research 39, 87–98.

[4] Banerjee, A., Chandrasekhar, A.G., Duflo, E., Jackson, M.O., 2013. The diffusion of microfinance. Science 341, 1236498.

[5] Bass, F.M., 1969. A new product growth for model consumer durables. Management science 15, 215–227.

[6] Bicchieri, C., 2016. Norms in the wild: How to diagnose, measure, and change social norms. Oxford University Press.

[7] Borgatti, S.P., 2006. Identifying sets of key players in a social network. Computational & Mathematical Organization Theory 12, 21–34.

[8] Borgatti, S.P., Mehra, A., Brass, D.J., Labianca, G., 2009. Network analysis in the social sciences. Science 323, 892–895.

[9] Catalini, C., Tucker, C., 2017. When early adopters don't adopt. Science 357, 135–136.

[10] Cencetti, G., Contreras, D.A., Mancastroppa, M., Barrat, A., 2023. Distinguishing simple and complex contagion processes on networks. Physical Review Letters 130, 247401.

[11] Centola, D., 2018. How behavior spreads: The science of complex contagions. volume 3. Princeton University Press Princeton, NJ.

[12] Centola, D., Macy, M., 2007. Complex contagions and the weakness of long ties. American journal of Sociology 113, 702–734.

[13] Coleman, J., Katz, E., Menzel, H., 1957. The diffusion of an innovation among physicians. Sociometry 20, 253–270.

[14] Constantino, S.M., Sparkman, G., Kraft-Todd, G.T., Bicchieri, C., Centola, D., Shell-Duncan, B., Vogt, S., Weber, E.U., 2022. Scaling up change: A critical review and practical guide to harnessing social norms for climate action. Psychological Science in the Public Interest 23, 50–97.

[15] Douglas, B.D., Ewell, P.J., Brauer, M., 2023. Data quality in online human-subjects research: Comparisons between MTurk, Prolific, CloudResearch, Qualtrics, and SONA. PLOS ONE 18, e0279720.

[16] Durlauf, S.N., 1999. How can statistical mechanics contribute to social science? Proceedings of the national academy of sciences 96, 10582–10584.

[17] Efferson, C., Vogt, S., Fehr, E., 2020. The promise and the peril of using social influence to reverse harmful traditions. Nature Human Behaviour 4, 55–68.

[18] Gavrilets, S., Tverskoi, D., Sánchez, A., 2024. Modelling social norms: an integration of the norm-utility approach with beliefs dynamics. Philosophical Transactions of the Royal Society B 379, 20230027.

[19] Goldenberg, J., Libai, B., Muller, E., 2010. The chilling effects of network externalities. International Journal of Research in Marketing 27, 4–15.

[20] Granovetter, M., 1978. Threshold models of collective behavior. American Journal of Sociology 83, 1420–1443.

[21] Guilbeault, D., Becker, J., Centola, D., 2018. Complex contagions: A decade in review. Complex Spreading Phenomena in Social Systems , 3–25.

[22] Guilbeault, D., Centola, D., 2021. Topological measures for identifying and predicting the spread of complex contagions. Nature Communications 12, 1–9.

[23] Hein, M., Kurz, P., Steiner, W.J., 2019. On the effect of hb covariance matrix prior settings: A simulation study. Journal of choice modelling 31, 51–72.

[24] Hein, M., Kurz, P., Steiner, W.J., 2020. Analyzing the capabilities of the hb logit model for choice-based conjoint analysis: a simulation study. Journal of Business Economics 90, 1–36.

[25] Horsevad, N., Mateo, D., Kooij, R.E., Barrat, A., Bouffanais, R., 2022. Transition from simple to complex contagion in collective decision-making. Nature Communications 13, 1442.

[26] Kuran, T., 1989. Sparks and prairie fires: A theory of unanticipated political revolution. Public Choice 61, 41–74.

[27] Lionberger, H.F., 1960. Adoption of new ideas and practices. A summary of the research dealing with the acceptance of technological change in agriculture, with implications for action in facilitating such change. Iowa State University Press.

[28] Lopez-Pintado, D., Watts, D.J., 2008. Social influence, binary decisions and collective dynamics. Rationality and Society 20, 399–443.

[29] Lü, L., Chen, D., Ren, X.L., Zhang, Q.M., Zhang, Y.C., Zhou, T., 2016. Vital nodes identification in complex networks. Physics Reports 650, 1–63.

[30] MacCoun, R.J., 2012. The burden of social proof: Shared thresholds and social influence. Psychological Review 119, 345.

[31] Miller, K.M., Hofstetter, R., Krohmer, H., Zhang, Z.J., 2011. How should consumers' willingness to pay be measured? an empirical comparison of state-of-the-art approaches. Journal of Marketing Research 48, 172–184.

[32] Mittal, D., Constantino, S.M., Vasconcelos, V.V., 2024. Anticonformists catalyze societal transitions and facilitate the expression of evolving preferences. PNAS Nexus 3, pgae302.

[33] Morone, F., Makse, H.A., 2015. Influence maximization in complex networks through optimal percolation. Nature 524, 65–68.

[34] Muller, E., Peres, R., 2019. The effect of social networks structure on innovation performance: A review and directions for research. International Journal of Research in Marketing 36, 3–19.

[35] Notarmuzi, D., Castellano, C., Flammini, A., Mazzilli, D., Radicchi, F., 2022. Universality, criticality and complexity of information propagation in social media. Nature Communications 13, 1308.

[36] Ott, M.Q., Light, J.M., Clark, M.A., Barnett, N.P., 2018. Strategic players for identifying optimal social network intervention subjects. Social Networks 55, 97–103.

[37] Peres, R., Muller, E., Mahajan, V., 2010. Innovation diffusion and new product growth models: A critical review and research directions. International Journal of Research in Marketing 27, 91–106.

[38] Robins, G., Lusher, D., Broccatelli, C., Bright, D., Gallagher, C., Karkavandi, M.A., Matous, P., Coutinho, J., Wang, P., Koskinen, J., et al., 2023. Multilevel network interventions: Goals, actions, and outcomes. Social Networks 72, 108–120.

[39] Rogers, E.M., 2010. Diffusion of Innovations. Simon and Schuster.

[40] Rossi, P.E., Allenby, G.M., Misra, S., 2024. Bayesian Statistics and Marketing. John Wiley & Sons.

[41] Ryan, B., Gross, N., 1950. Acceptance and diffusion of hybrid seed corn in two iowa communities. Iowa Agricultural Experiment Station Research Bulletin 372.

[42] Sassine, J.G., Rahmandad, H., 2024. How does network structure impact socially reinforced diffusion? Organization Science 35, 52–70.

[43] Sermas, R., 2022. ChoiceModelR: Choice Modeling in R. URL: https://CRAN.R-project.org/package=ChoiceModelR. r package version 1.3.0.

[44] Valente, T.W., 1995. Network models of the diffusion of innovations. Hampton Press.

[45] Valente, T.W., 1996. Social network thresholds in the diffusion of innovations. Social networks 18, 69–89.

[46] Valente, T.W., 2010. Social networks and health: Models, methods, and applications. Oxford University Press.

[47] Valente, T.W., 2012. Network interventions. Science 337, 49–53.

[48] Valente, T.W., Davis, R.L., 1999. Accelerating the diffusion of innovations using opinion leaders. The Annals of the American Academy of Political and Social Science 566, 55–67.

[49] Valente, T.W., Saba, W.P., 1998. Mass media and interpersonal influence in a reproductive health communication campaign in bolivia. Communication Research 25, 96–124.

[50] Valente, T.W., Vega Yon, G.G., 2020. Diffusion/contagion processes on social networks. Health Education & Behavior 47, 235–248.

[51] Watts, D., 2002. A simple model of global cascades on random networks. Proceedings of the National Academy of Sciences of the United States of America 99, 5766–5771.

[52] Yang, L., Constantino, S.M., Grenfell, B.T., Weber, E.U., Levin, S.A., Vasconcelos, V.V., 2022. Sociocultural determinants of global mask-wearing behavior. Proceedings of the National Academy of Sciences 119, e2213525119.

“Integrating behavioral experimental findings into dynamical models to inform social change interventions”: Reply Letter

Dear Editor,

We are very pleased to submit the revised version of the article “Integrating behavioral experimental findings into dynamical models to inform social change interventions" to *Nature Human Behaviour*. We would like to thank you and the Reviewers for all the suggestions, which helped us to further expand the robustness tests for the choice modeling results as well as improve the clarity of the notation.

In the rest of this document, we address all the points the Reviewers raised, which has resulted in a revision of the manuscript. Before addressing each point in detail, we wish to provide you with a summary of the main changes:

- As required by Reviewer 2, we have clarified the description of how the individual partworths were generated (Supplementary Note S3 A); extended the sensitivity analysis to scenarios where the distribution of the ground-truth parameters is multimodal (Supplementary Note S3 D); added remarks on the potential role of interactions between the partworths to the revised Methods section.
- Following Reviewer 3’s advice, we have adapted the mathematical notation in Supplementary Note S6, to ensure notation consistency throughout the entire Section.

In addition to the changes made in response to the reviewers’ comments, we have refactored the code to make it easier for interested readers to replicate our results and conduct their own analyses. In doing so, we made a few minor updates, which we list at the end of this letter. These updates did not alter our conclusions.

We hope that the changes we made satisfactorily address the Reviewers’ concerns, and we are very much looking forward to your feedback.

Best regards,
The authors

RESPONSE TO REVIEWER 2

> *First, apologies to the editors and reviewers for the delayed review.*
> *I appreciate the extensive efforts from the authors to revise the draft in light of my somewhat outside-of-the-discipline concerns. Overall, I'm happy with the edits and they have largely satisfied my original concerns. In particular, I appreciate the explicit mention that the individual-level thresholds are identified by way of the parametric assumptions of the model – namely that our prior belief is that the distribution of individual level has a particular parametric form. While I understand that the marketing literature considers these assumptions relatively unproblematic, I don't think this is as widely shared - especially in my corner of the social science world, so it's good that the paper is clear about the kind of intellectual "gamble" being made.*

We thank the Reviewer for their positive evaluation of our efforts, as well as their constructive comments regarding the prior beliefs embedded in the model estimation. The Reviewer's feedback has helped us substantially strengthen the robustness of our results and the discussion of the assumptions.

> *I think the exercise in S3 is helpful for characterizing sensitivity to the modeling assumptions and I'm glad to see it. I'm unsurprised that the uninformative prior generates significant problems – some regularization is essential for the kind of task being undertaken here. And it's good to see little variation as the strength of the prior towards homogeneity changes. My only concern is in the way that the ground truth is being generated. It's a little unclear from the explanation what it means that the "ground-truth" utilities were generated from the estimates in the model? What – if any – heterogeneity is there in the unit-level part-worths? If these are the ones obtained from the individual-level HB estimates, then I worry that the simulation study is a bit too "easy" – the heterogeneity will follow the nice normal structure imposed by the simulation.*

The Reviewer correctly understood that we set the ground-truth partworth utilities to the HB estimates from the conjoint studies (i.e., the means of the posterior distributions). We made this clearer by adding the following statement to Supplementary Note S3 A, where we describe the survey simulation:

"To calibrate the choice simulations with the empirical data from the conjoint experiments, we endow each agent with a ground-truth partworth utility vector equal to the estimated partworth utilities of a participant selected at random from the pool of real-world participants without replacement. The resulting distributions of partworth utilities across agents are shown in Figs. S9−S10"

> *What I think would help push the prior sensitivity a bit more is to consider more "problematic" distributions – particularly multi-modal ones. Maybe consider a simulation with two latent groups - those for whom the partworth coefficient is positive and another for whom its negative? I would like to know how much the *normality* in the prior structure bites in addition to the prior variance (where, with sufficient data, the likelihood should overcome all but the strongest prior spec).*

We agree with the Reviewer that our previous analysis may have not fully captured the method's sensitivity in scenarios where the underlying partworth distributions are more complex or irregular than those of the HB estimates we used as ground-truth. Following the Reviewer's advice, in novel Supplementary Note S3 D, we expanded the sensitivity analysis to scenarios in which the ground-truth partworth distributions are not the direct result of the HB estimation, but instead follow multimodal distributions.

To construct the multimodal distributions, we considered two scenarios with increasing separation between the distributional peaks. Starting from the calibrated distributions (*i.e.,* the distributions estimated through HB, shown in new Figs. S9 and S10), for each partworth utility, we randomly selected 50% of the agents and shifted their utilities by an amount equal to the range of the calibrated distribution multiplied by a shift factor $d \in \{1, 2\}$. The resulting distributions of selected ground-truth partworth utilities for different values of d are illustrated in novel Fig. S12. Based on the obtained multimodal distributions, we performed new choice simulations and conducted the same individual-level sensitivity analysis we performed for the calibrated distributions.

For the informative priors, we found that the results support our initial conclusion: the partworth estimation accuracy, threshold estimation accuracy, and the correlation between true and estimated thresholds remain similar across prior specifications. Interestingly, in the multimodal scenario, we no longer observe a disadvantage of the uninformative prior for the threshold estimation. Furthermore, the uninformative prior outperforms the tighter ones in partworth recovery. Arguably, this occurs because when the true distribution of partworths is multimodal, a tighter normal prior induces stronger shrinkage of individual estimates toward the global mean, thereby masking the underlying multimodality and increasing bias [6].

Overall, these results show that under more challenging, multimodal preference structures, informative normal priors do not necessarily outperform the uninformative prior. As expected, the correlation between estimated and ground-truth thresholds decreases relative to the calibrated simulations. Nevertheless, the correlations remain high across all scenarios [larger than r = 0.74 (r = 0.79) in the PS (AA) Study], pointing to a reasonable robustness of the estimated thresholds to different preference structures. We leave improvements in threshold estimation for

multimodal scenarios to future research and note that, when multimodality is expected, extensions of the HB model (such as those using mixtures of normals [6]) might improve estimation accuracy.

> *Otherwise, the justification of the linearity assumption for the adoption attribute seems feasible - at a minimum, the linear approximation doesn't appear to lose all that much (Except maybe at the top end where you see minimal difference between 76 and 98). I'm not too troubled by the interpolation here.*
>
> *So overall, I'd push towards a conditional accept with a bit more clarity on the nature of *exactly* how the individual partworths were generated from the recovered applied parameters (my understanding is that the study is just using the estimated β_n, so there is heterogeneity, but of a very particular type) and maybe an additional variation on the simulation that breaks the single-mode structure of the individual partworths (assuming the above is correct re: how they're estimated). If it does seem to perform poorly, I don't think this is a *problem* for the final results - just a useful caveat on how we should think about the value of adoption.*

We thank the Reviewer for the positive recommendation. Following their advice, we have clarified the description of how the individual partworths were generated (Supplementary Note S3 A), and extended the sensitivity analysis to cases where the distribution of the ground-truth parameters is multimodal (Supplementary Note S3 D).

> *Something I didn't raise in the original review (so I don't think it's *critical* here) is to possibly consider interactions between the partworths as well? How much does the additivity and separability in the utility model "bite" as an assumption? Given my experience with the marketing literature, this tends to be an important assumption (otherwise, the parameter space gets unwieldy quite fast), but I do wonder how salient it is. Possibly a sentence or two remarking on this alongside the other model-sensitivity remarks would help.*

We agree with the Reviewer that the additivity and separability assumption can be restrictive in applications where interactions play a substantial role. Conceptually, the adoption threshold can still be analytically derived from the partworth utilities even in the presence of interactions, as demonstrated in revised Supplementary Note S6 B. At the same time, as noted by the Reviewer, the estimation of models with many interaction terms can be challenging in practice. In response, we added a remark in the revised Methods section acknowledging this limitation, while noting that interaction effects may still be incorporated by restricting attention to a small set of theoretically motivated terms and by adapting the conjoint design accordingly.

"An additive and separable model rules out interaction effects, which can be important in some real-world applications. Although interaction models can still yield a simple analytic threshold (see Supplementary Note S6 B), estimating models with a large number of interaction terms can pose substantial challenges due to model complexity [2]. In applications where interactions are essential, this concern may be partly alleviated by restricting interactions to a small set of theory-motivated effects [5], and by adopting conjoint designs and estimation techniques tailored to identify them [1, 5]."

RESPONSE TO REVIEWER 3

> *I thank the authors for taking the time to read the additional literature and for thoroughly addressing not only my points but also those of the other reviewers. The additional sensitivity analysis makes the results even more convincing. The paper is now well contextualized and provides an accurate account of its contribution, showing how it clearly moves the field forward. I still think the connection to Bass diffusion is a bit of an extra bit connecting complex to simple contagion. This is, however, more of a personal taste in paper style and not a judgment of the quality. If you really want to keep the bit, then I would fully explain that, when ϵ_{ni} is iid and Gumbel$(0, \sigma)$, $\pi_{ni} = \exp(U^*_{ni}/\sigma)/\sum \exp(U^*_{nj}/\sigma)$, i.e., the exponentials are divided by sigma (which means that your clarity parameter $c = 1/\sigma$). I think that is cleaner than the abuse of notation "It is useful to consider a clarity parameter [34] and, with a slight abuse of notation, rewrite the utility from alternative i in the form". Alternatively, maybe even simpler, just define $U_{ni} = U^*_{ni} + (1/c)\epsilon_{ni}$, where ϵ is the Gumbel$(0, 1)$ you define and the higher c, the clarity, the lower the noise. And everything follows without abuse of notation.*

We thank the Reviewer for their constructive feedback which helped us better contextualize our research, and for their remaining comment which helps us improve the notation. We agree with the Reviewer's suggestion to specify the clarity parameter of the choice model from the very beginning of Supplementary Note S6, without altering the notation at a later stage. To implement this, we adopted a variant of the Reviewer's second suggestion. In particular, in the revised Supplementary Note S6, we follow the notation in ref. [7], which defines a choice model with a scale parameter λ (equivalent to the clarity parameter in our SM). With this notation, the utility from adopting is $U_{ni} = \lambda\, U^*_{ni} + \epsilon_{ni}$, from which it follows that

$$\pi_{ni} = \frac{\exp\left(\lambda\, U^*_{ni}\right)}{\sum_{j=1}^{I} \exp\left(\lambda\, U^*_{nj}\right)}, \tag{1}$$

where λ is equivalent to the clarity parameter in our original manuscript and can be interpreted as an inverse temperature in statistical physics terms [4].

Technical changes

During the code refactoring we made minor adjustments to ensure full consistency between the code and the main text. To ensure the results presented in the manuscript match the updated implementation, we repeated the analysis and updated the manuscript accordingly. Below we summarize the main changes, none of which led to qualitatively different insights.

- We have corrected an inconsistency in the complex centrality calculation, for which we adapted the code accompanying Ref. [3]. In this implementation, to calculate a given node *i*'s score, one runs a threshold-based diffusion process where *i* and a number of nodes in its neighborhood are activated.

  In our setup, each diffusion process is initialized with a fixed number of seeds $S = \lfloor z\,N \rfloor$. For those nodes whose second-order neighborhood contained less than S nodes, the original code only activated $d_i^{(2)} < S$ nodes (where $d_i^{(2)}$ denotes the total number of unique nodes within the second-order neighborhood of focal node *i*), and did not recursively search beyond that neighborhood for additional eligible seeds. We have addressed this by implementing a recursive search of the seeds into higher-order neighborhoods until S seeds are found and activated. Through the new implementation, the complex centrality score is now based on simulations starting from S seed nodes for all the nodes (including those with $d^{(2)} < S$).

  Our results did not qualitatively change overall. The only exception is that, in the updated results, the uncalibrated complex centrality (UCC) now outperforms random seeding in the centrality-based cost scenario of the AA Study. This does not alter our previous conclusion that the uncalibrated complex centrality (UCC) is a suboptimal seed selection method compared to the calibrated complex centrality (CCC), as UCC remains substantially worse than CCC across all scenarios, and it still performs worse than random seeding when a smaller seed-set size is used (Figs. S15-S16).

- In the previous version of the manuscript, there was a minor discrepancy between the formulas for the cost reported in the Methods and the actual implementation. To remove this discrepancy, in the preference-based cost, we brought the offset cost c_0 outside of the sum, which represents a fixed cost-per-intervention (Eq. 5) and matches our implementation. An offset cost per intervention was also implemented in the centrality-based cost, which we added in the revised manuscript (Eq. 6).

- In the previous version of the manuscript, we stated that individuals with negative utility for the social signal (5 participants in the PS Study and 1 participant in the AA Study, see Supplementary Notes S1 B−S1 C) were excluded, but this exclusion was mistakenly not implemented in the code. We have now corrected the code to remove these individuals from the analysis. There was no significant change in the updated results compared to the previous ones.

REFERENCES

[1] Chrzan, K., Orme, B., 2000. An overview and comparison of design strategies for choice-based conjoint analysis. Sawtooth Software Research Paper Series 98382, 161–178.

[2] Green, P.E., Srinivasan, V., 1990. Conjoint analysis in marketing: New developments with implications for research and practice. Journal of Marketing 54, 3–19.

[3] Guilbeault, D., Centola, D., 2021. Topological measures for identifying and predicting the spread of complex contagions. Nature Communications 12, 1–9.

[4] Marsili, M., 1999. On the multinomial logit model. Physica A: Statistical Mechanics and its Applications 269, 9–15.

[5] Rao, V.R., 2014. Applied conjoint analysis. Springer Science & Business Media.

[6] Rossi, P.E., Allenby, G.M., Misra, S., 2024. Bayesian Statistics and Marketing. John Wiley & Sons.

[7] Train, K.E., 2009. Discrete choice methods with simulation. Cambridge University Press.
